# Advances in Chitosan Derivatives: Preparation, Properties and Applications in Pharmacy and Medicine

**DOI:** 10.3390/gels10110701

**Published:** 2024-10-29

**Authors:** Dominika Žigrayová, Veronika Mikušová, Peter Mikuš

**Affiliations:** 1 Department of Galenic Pharmacy, Faculty of Pharmacy, Comenius University Bratislava, Odbojárov 10, 83232 Bratislava, Slovakia; zigrayova1@uniba.sk (D.Ž.); mikusova@fpharm.uniba.sk (V.M.); 2 Department of Pharmaceutical Analysis and Nuclear Pharmacy, Faculty of Pharmacy, Comenius University Bratislava, Odbojárov 10, 83232 Bratislava, Slovakia; 3 Faculty of Pharmacy, Toxicological and Antidoping Center, Comenius University Bratislava, Odbojárov 10, 83232 Bratislava, Slovakia

**Keywords:** chitosan derivatives, derivatization schemes, wound healing, tissue engineering, drug delivery

## Abstract

Chitosan (CS) derivatives have been extensively investigated to enhance the physicochemical and biological properties of CS, such as its solubility, biocompatibility, and bioactivity, which are required in various areas of pharmacy and medicine. The present work emphasizes the ongoing research and development in this field, suggesting that the further exploration of CS derivatives could lead to innovative solutions that benefit society. The physicochemical properties, biological activities, methods of preparation, advantages, limitations, intended application areas, and realized practical implementations of particular CS derivatives are summarized and discussed herein. Despite the numerous promising attributes of CS derivatives as reported in this paper, however, challenges like target selectivity, standardization (purity, chitosan structural variability), and cost-effectiveness still need addressing for widespread implementation, especially in drug delivery. Therefore, basic research studies still prevail in CS drug delivery systems. However, for specific applications such as wound healing and tissue engineering, implementations of CS derivatives in practice are found to be more frequent. To obtain a more complex view of the topic, information from the scientific papers reviewed is supplemented with information from actual patents and clinical studies. Both basic research advances and the most successful and important medical implementations of CS derivatives are discussed concerning further challenges and future perspectives.

## 1. Introduction

**Figure 1:**
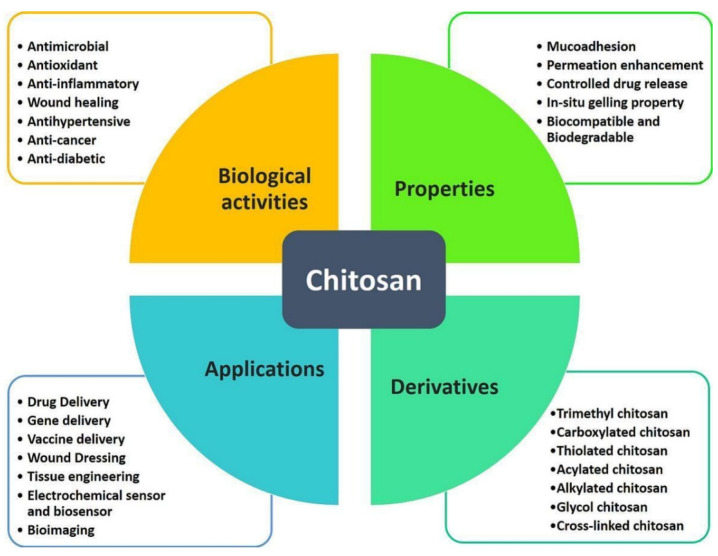
Properties, biological activity, and applications of chitosan and its derivatives [2].

Chitosan (CS) is a biopolymer derived from chitin, the structural component found in the exoskeletons of crustaceans like shrimp and crabs. It is known for its biocompatibility, biodegradability, and non-toxicity, making it widely applicable in biomedicine, agriculture, and the food industry [1]. To extend its range of applications, CS can be chemically or physically modified, resulting in derivatives with enhanced properties, such as improved solubility, physicochemical stability, and bioactivity [2]. Figure 1 provides an overview of CS’s key properties, biological activity, and applications and its derivatives.

**Figure 2:**
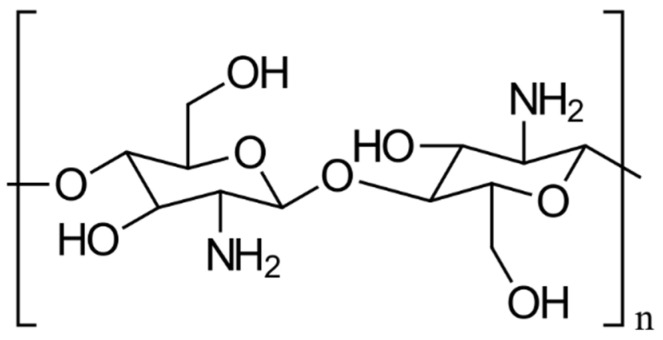
Chemical structure of CS.

CS consists of repeating units of β-(1→4)-linked *D*-glucosamine and *N*-acetyl-*D*-glucosamine, as depicted in Figure 2 [3]. Its structure is analogous to that of cellulose but features an additional amino group at the C-2 position of the glucosamine units, which imparts distinctive properties to CS, facilitating chemical modifications and interactions with various molecules. The glycosidic bonds in CS, specifically β-(1→4) linkages, connect the anomeric carbon of one glucose unit to the fourth carbon of the adjacent glucose unit, contributing to its linear structure, which balances stability and flexibility. The amino group (NH_2_) at the C-2 position is the key functional group in CS, capable of undergoing modifications like acetylation, phosphorylation, and quaternization to adjust CS’s properties for specific pharmaceutical uses. For example, acetylation alters the amino groups by introducing acetyl groups (CH_3_CO-), thereby modifying CS’s solubility, charge density, and interaction capabilities. CS’s versatility is further enhanced through chemical modifications, such as grafting carboxyl, sulfate, or hydroxyl groups onto its backbone, enabling the development of CS derivatives with specific, tailored properties [3].

The primary reactive groups in chitosan (CS) are the amino (-NH_2_) and hydroxyl (-OH) groups, which can participate in reactions with acids, bases, and crosslinking agents. This reactivity allows for the customization of CS’s properties for specific applications. In the field of pharmacy, common CS derivatives include chitosan hydrochloride (CS HCl), known for its improved water solubility; *N*-trimethyl chitosan (TMCS), which offers enhanced solubility and bioavailability; and carboxymethyl chitosan (CMC), which is suitable for drug delivery systems and wound healing. Low-molecular-weight chitosan, such as chitosan oligosaccharides (COS), has potential therapeutic applications in wound healing and anti-inflammatory treatments. These derivatives provide enhanced bioactivity, targeted drug delivery, controlled release, increased solubility and bioavailability, and better compatibility with other pharmaceutical ingredients [3,4]. Ongoing research continues to explore various other CS modifications, as extensively documented in the literature.

**Figure 3:**
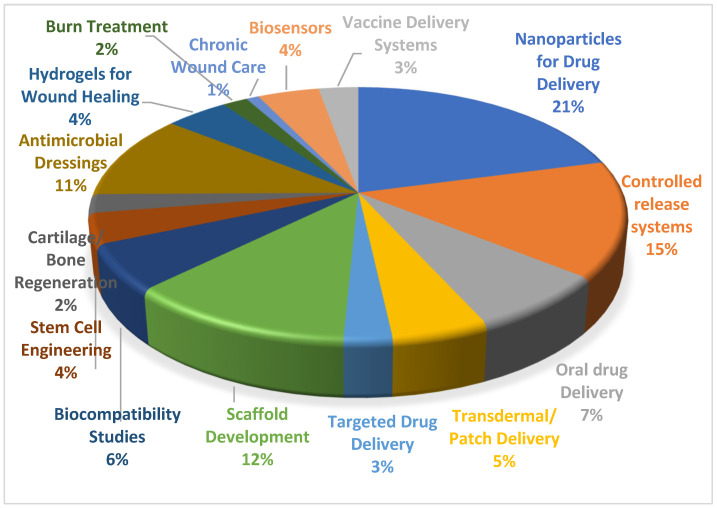
Biomedical applications of chitosan derivatives in research articles according to WoS in the period of 2000–2024.

In recent years, the number of publications on chitosan derivatives has significantly increased, reflecting the growing interest in this area. According to the current data, gathered in the Web of Science database in the period of 2000–2024, there are over 5000 research articles and reviews published on the topic of chitosan and its derivatives. Out of these, a large proportion focuses on their applications in drug delivery, especially nanoparticulate drug delivery, tissue engineering, and wound healing. Figure 3 illustrates the application fields of chitosan derivatives in these articles. For researchers looking to develop new derivatives, these statistics underscore the importance of understanding the current advancements and identifying unexplored areas for innovation.

Recent reviews have extensively discussed various chitosan (CS) derivatives and their applications. Mohite et al. [5] provided a comprehensive overview of advances in the grafting of chitosan and chito-oligosaccharides (CS/COS), highlighting their use in drug delivery systems for small drug molecules, proteins, and RNA interference therapeutics. Wang et al. [6] discussed how modifications of chitosan improve its properties and then generally discussed the applications of chitosan derivatives in biomedical fields. Abourehab et al. [7] described the properties of chitosan, its methods of preparation and its modifications, and the most common applications of chitosan. Shrestha et al. [8] discussed the methods of preparing chitosan derivatives and their use specifically in antimicrobial drug delivery and agricultural applications. Petroni et al. [9] discussed chitosan and its functionalization and potential applications in material science.

Many reviews have concentrated on specific types of CS derivatives or their applications. For instance, Pathak et al. [10] reviewed quaternized CS-based delivery systems, discussing their synthesis, properties, biomedical applications, and challenges, particularly in tissue engineering, wound healing, and gene and vaccine delivery. Other reviews have focused on CS derivatives in particular therapeutic areas. For example, Rout et al. [11] summarized the key biological properties of CS and its derivatives, such as quaternized and thiolated CS, which are effective in treating oral mucositis. Xia et al. [12] explored the structures and biological characteristics of CS and its derivatives, including *N*-acylated and quaternized CS, designed for the treatment of skin and soft tissue diseases. This review also discussed recent advances and future trends in the use of CS derivatives for therapeutic purposes, such as anti-infection, wound healing, tissue regeneration, and anticancer effects in soft tissue diseases. Confederat et al. [13] examined the physicochemical and pharmacological properties of various CS derivatives, including CS trimethylate, CS thiolate, hydroxypropyl CS, alkylated CS, and phosphorylated CS, with a focus on their antimicrobial potential, their mechanisms of action, the factors influencing their antimicrobial activity, and their effectiveness against resistant strains.

This work summarizes and evaluates the latest (until September 2024) knowledge in CS derivatives in a wider context, i.e., including all of the most important and frequently used CS derivatives in various application areas. It discusses recent advances in CS’s structural modifications, reaction schemes, and preparation procedures, highlighting the improved properties of the resulting CS derivatives compared with native CS and their possible applications in pharmacy, biomedicine, and bioengineering, as well as the extent of their practical implementation in these fields. Unlike most previous studies, which focus on specific applications of specific derivatives/groups of derivatives, this review emphasizes improvements in and practical applications of chitosan derivatives across various fields, including drug delivery, tissue engineering, and wound healing as their main application areas. Although the vast majority of the published papers deal with basic research on CS derivatives, this review also discusses the extent of the practical implementation of CS derivatives resulting from the patent literature and ongoing clinical studies. Such an approach should contribute to a better understanding of the potential of chitosan derivatives and their future practical possibilities in modern medicine. This review could be very beneficial for researchers who are interested in chitosan modifications, aiming to develop new derivatives for specific applications, and need a wider view of the topic, along with recognizing areas of basic research and real implementations.

## 2. Classification of CS Derivatives

CS derivatives can be classified according to various criteria, such as the structural characteristics and properties, preparation approaches, or application areas. In this work, we classify CS derivatives in the following sections, discussing their preparation approaches (Section 3) and their structural characteristics, properties, and resulting application potential and practical implementations (Section 4). All physicochemical properties, biological properties, methods of preparation, advantages, limitations, intended application areas, and realized practical implementations of particular CS derivatives extracted from recently published papers and critically evaluated in Section 3 and Section 4 are summarized in Tables within the manuscript.

## 3. Preparation Approaches for CS Derivatives

**Figure 4:**
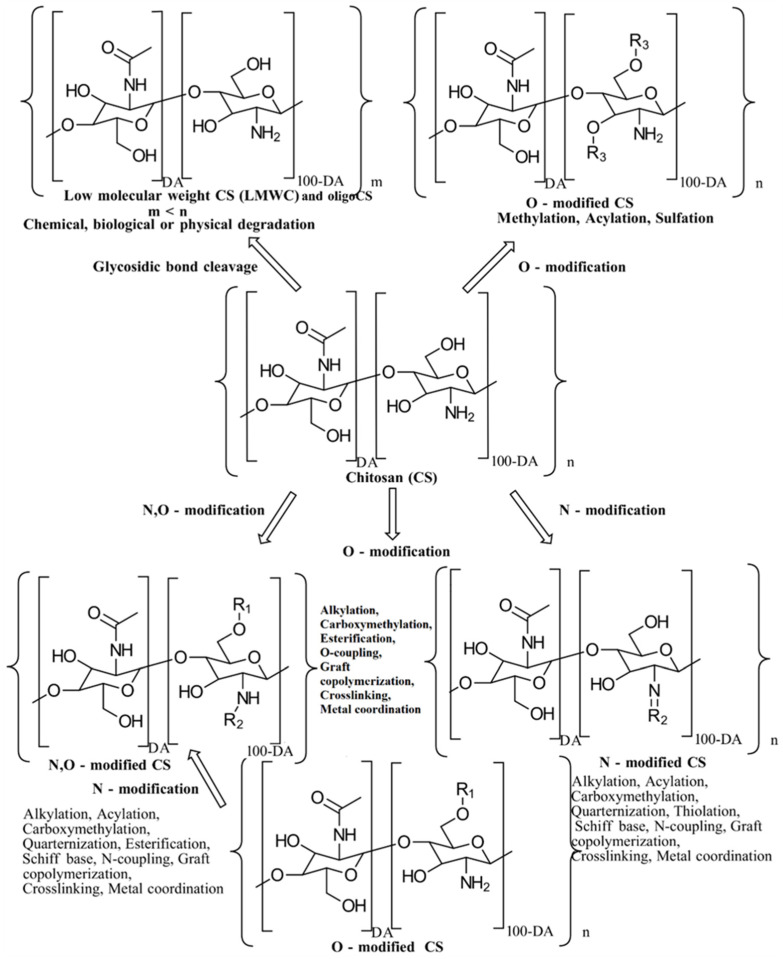
The active sites on the chitosan surface that are structurally modifiable, as well as the numerous techniques for their alteration that have been comprehensively studied [4].

CS derivatives are classified and discussed here according to their preparation approaches, including alkylation (Section 3.1), acylation (Section 3.2), other covalent-based synthetic approaches (Section 3.3), and physical-based preparation approaches (Section 3.4). Conventional and innovative preparation procedures for CS derivatives are briefly evaluated. The possibilities for the chemical modification of CS are illustrated in Figure 4 [4].

### 3.1. Alkylation

**Figure 5:**
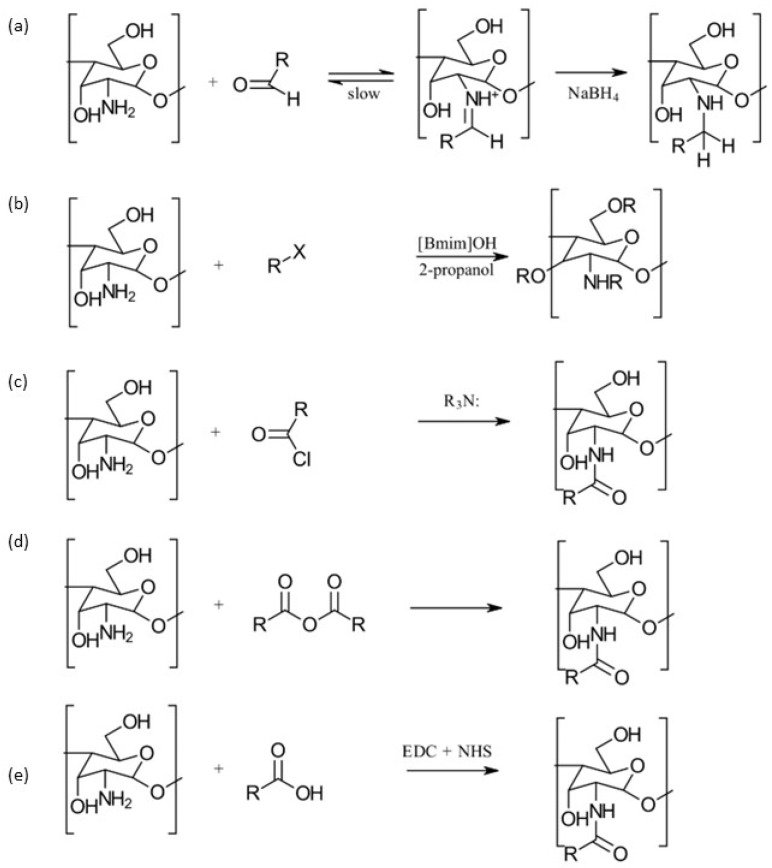
A scheme depicting alkylated chitosan obtained through different chemical reactions, including (**a**) reductive alkylation, where an aldehyde or ketone reacts with the amino group of chitosan; (**b**) alkylation with haloalkanes, targeting the amino group; (**c**) alkylation with acyl chlorides, affecting the hydroxyl or amino groups; (**d**) alkylation with anhydrides, introducing alkyl groups to the amino or hydroxyl groups; and (**e**) alkylation with fatty acids, adding long-chain alkyl groups through esterification [14].

An alkylation reaction involves introducing an alkyl group to the C2-NH_2_, C6-OH, or C3-OH groups of chitosan (CS), forming an alkyl-group-containing CS derivative. Specifically, *N*-alkylation occurs when the alkyl group is added to the C2-NH_2_ group, while *O*-alkylation involves the C6-OH or C3-OH groups [14,15]. Although both the amino group (-NH_2_) and the hydroxyl groups (C3, C6-OH) can participate in CS alkylation reactions, the amino group reacts more rapidly and effectively, providing better protection for other functional groups. This is because the C2-NH_2_ group has a strong nucleophilic lone pair of electrons, causing CS alkylation to predominantly occur through the amino group, resulting in *N*-alkylated CS derivatives [14]. *N*-alkylated CS can be synthesized using a halogenated alkane [6], as well as higher fatty aldehydes and long-chain fatty acyl groups [16]. Chen [16] successfully synthesized a series of *N*-alkylated CS molecules. Figure 5 [14] depicts the various chemical reactions commonly employed for CS alkylation.

#### 3.1.1. Carboxymethylation

**Figure 6:**
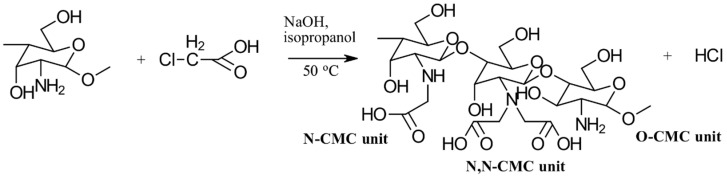
Synthesis of carboxymethyl CS derivatives.

Carboxymethyl CS is the most thoroughly investigated derivative of CS. When subjected to controlled reaction conditions, it can produce various products with different selectivity and degrees of substitution. The primary carboxymethylation products of CS are *N*-carboxymethyl CS (NCMC) and *O*-carboxymethyl CS (OCMC), along with their combinations, such as *N*,*O*-carboxymethyl CS (NOCMC) and *N*,*N*-carboxymethyl CS (NNCMC). Figure 6 provides examples of the chemical structures of these carboxymethyl CS derivatives [17].

Carboxymethyl CS is synthesized through carboxymethylation, a process in which some of the -OH groups of CS are replaced with -CH_2_COOH groups. Despite this modification, the reactive -COOH and -NH_2_ groups remain available for further chemical alterations to enhance their physical properties, particularly for applications in metal chelation and dye binding. These additional modifications can be achieved through (i) reductive alkylation or (ii) direct alkylation.

(i) In reductive alkylation, the -NH_2_ group of the CS unit reacts with the carbonyl group of aldehyde-glyoxylic acid, followed by hydrogenation with NaBH_4_ or NaCNBH_3_ to form *N*-carboxymethyl CS. This method specifically introduces the carboxymethyl substituent onto the *N*-atom without affecting the *O*-atom. The ratio of mono- to di-carboxymethylated glucosamine units in CS is influenced by the ratio of amine (CS) to the reagent and the reaction conditions. Muzzarelli et al. [18], who initially reported this technique, created a range of products from various types of CS with differing molecular sizes, molecular weight distributions, and degrees of deacetylation by using different quantities of glyoxylic acid.
(ii) The direct alkylation method involves using monohalocarboxylic acids, such as monochloroacetic acid, to create *N*-carboxyalkyl and *O*-carboxyalkyl CS derivatives under various reaction conditions. The choice of conditions affects the selectivity for *N*- versus *O*-carboxyalkylation and the degree of substitution. Initially, CS is activated by soaking it in an alkaline solution, such as water/isopropyl alcohol. When carboxymethylation is carried out with monochloroacetic acid in a mildly alkaline medium (pH 8–8.5), only the amino groups are activated, leading to *N*-substitution exclusively. The CS will precipitate at this pH but will gradually dissolve as the reaction progresses, resulting in mono- or di-*N*-substitution. In contrast, when using higher concentrations of alkali (over 25% aqueous NaOH), the reaction with monochloroacetic acid produces a mixture of *N*- and *O*-alkyl CS derivatives, with substitutions occurring at the C6 and C3-OH groups, as well as the C2-NH_2_ groups [18].

#### 3.1.2. Quaternization

**Figure 7:**
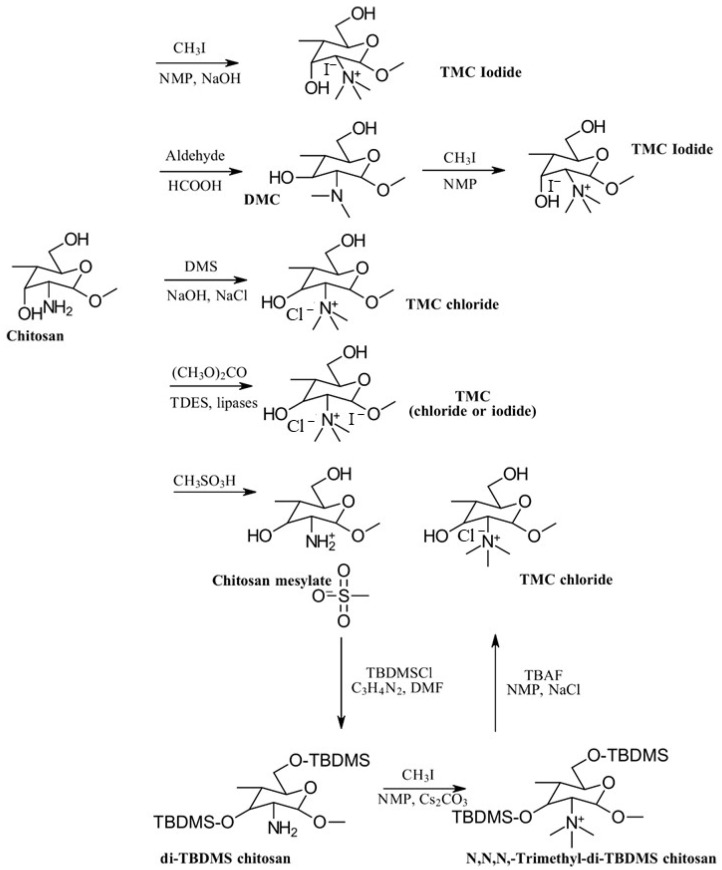
Synthesis of quaternized trimethyl CS (TMC) [1].

The most traditional and widely used quaternized chitosan derivatives include *N*,*N*,*N*-trimethyl chitosan (TMC) and *N*-[(2-hydroxy-3-trimethyl ammonium) propyl] chitosan (HTCC). More recently, quaternized chitosan has been produced by introducing pyridinium or phosphonium salts [1,19]. Figure 7 outlines the different methods of preparing TMC derivatives [1].

The simplest method for the synthesis of *N*,*N*,*N*-trimethyl chitosan (TMC) involves a one-step reaction where chitosan (CS) is treated with methyl iodide (CH_3_I) under strongly alkaline conditions, using *N*-methyl-2-pyrrolidone (NMP) as the solvent and sodium iodide (NaI) as the catalyst. This process typically results in a quaternization degree ranging from 10% to 45% [20]. Another approach, developed by Muzzarelli et al. [18], begins with the preparation of *N*,*N*-dimethyl chitosan (DMC) by reacting CS with formaldehyde in an acidic medium, followed by the addition of sodium borohydride. Methyl iodide (CH_3_I) is then introduced to this system to produce TMC.

Although methyl iodide (CH_3_I) is an effective methylation agent, it is costly, volatile, and toxic, and the halide ions that it produces are challenging to separate from the solution. To address these issues, De Britto et al. [21] developed a method for the synthesis of *N*,*N*,*N*-trimethyl chitosan (TMC) using dimethyl sulfate (DMS). This approach offers a more cost-effective, less toxic, and simpler alternative, making it greener. DMS serves both as a methylating agent and a solvent, thereby eliminating the need for *N*-methyl-2-pyrrolidone (NMP).

Methylation agents often lack selectivity, resulting in unintended *O*-methylation. To achieve the selective methylation of CS, a method has been developed that involves protecting less reactive groups through *O*-silylation. Initially, CS is reacted with methanesulfonic acid (CH_3_SO_3_H) to produce CS metasylate. This compound is then treated with tert-butyldimethylsilyl (TBDMS) groups to form 3,6-di-*O*-tert-butyldimethylsilyl-CS. With the hydroxyl groups protected, *O*-methylation is prevented, allowing for selective methylation with methyl iodide (CH_3_I). Finally, the protected hydroxyl groups are deprotected using a tetrabutylammonium fluoride (TBAF) solution in *N*-methyl-2-pyrrolidone (NMP) [22].

The properties, biological activity, and methods of preparation of alkylated CS derivatives, including carboxylated and quaternized derivatives, are illustrated in Table 1.

### 3.2. Acylation

**Figure 8:**
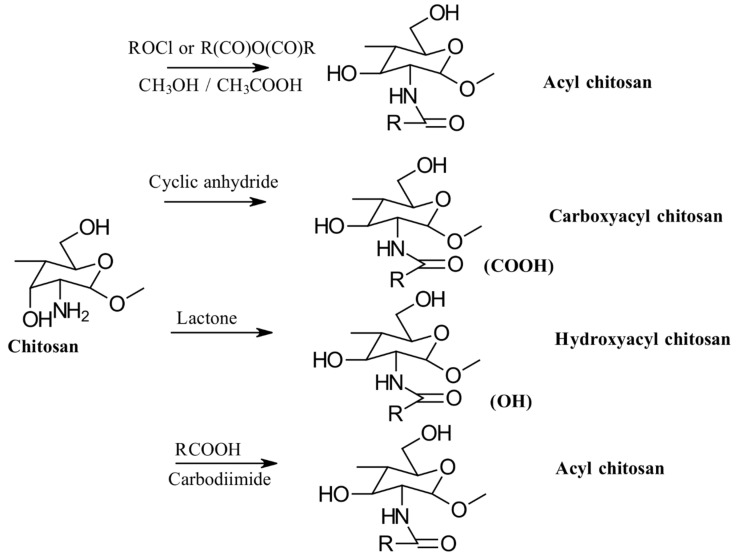
Synthesis route for acylated CS [24].

Acylation is one of the most frequently used modifications of CS. This process involves reacting CS with various organic acids and their derivatives, such as anhydrides and acyl chlorides, to incorporate aliphatic or aromatic acyl groups into the molecular chain. For a visual representation, see Figure 8 [24].

The acylation reaction disrupts both the intramolecular and intermolecular hydrogen bonding in CS, leading to reduced crystallinity and increased water solubility. CS contains two hydroxyl groups on its molecular chain: the primary hydroxyl group at C6-OH and the secondary hydroxyl group at C3-OH [24]. *N*-acylation refers to the acylation reaction at the C2-NH_2_ group, resulting in the formation of an amide [25]. In contrast, *O*-acylation occurs when the hydroxyl group at C6-OH is acylated, provided that a protective functional group is present at C2-NH_2_, leading to the formation of an ester [26].

*N*-acyl derivatives of CS are commonly produced using acyl chlorides and anhydrides. The acylation reactions are typically performed in various media, such as aqueous acetic acid/methanol, pyridine, pyridine/chloroform, trichloroacetic acid/dichloroethane, ethanol/methanol mixtures, methanol/formamide, or DMA-LiCl. Due to the distinct reactivity of the amino group and the two hydroxyl groups on the CS backbone, acylation can be selectively controlled to target either the amino group alone or both hydroxyl groups [26].

Cyclic acid anhydrides are employed for acylation via ring-opening reactions to produce *N*-carboxyacyl chitosan. Examples include succinic, maleic, glutaric, itaconic, phthalic, cis-1,2,3,6-tetrahydrophthalic, 5-norbornyl-endo-2,3-dicarboxylic, cis-1,2-cyclohexyldicarboxylic, trimellitic, (2-octen-1-yl) succinic, citraconic, and pyromellitic anhydrides. Additionally, *N*-acylated CS derivatives with both saturated (e.g., C2–C18) and unsaturated acyl groups (e.g., oleic, linoleic, elaidoic, erucoyl), as well as aromatic acyl groups (e.g., phthaloyl, p-nitrobenzoyl, cinnamoyl), have been successfully synthesized. These modifications result in randomly distributed substituents along the CS chain in a controlled manner.

Several derivatives of CS have been synthesized through *O*-acylation reactions. For instance, *N*,*O*-acyl CS was prepared by using methanesulfonic acid (MeSO_3_H) as the solvent, which has proven effective for this process [27]. Additionally, the synthesis of *O*,*O*-didecanoyl CS and *O*-succinyl CS involves first creating a protected *N*-phthaloyl CS intermediate. This approach requires several steps: initially, the amino group is protected through phthaloylation, followed by *O*-acylation; finally, the removal of the protecting group is performed using hydrazine hydrate [28]. This method is complex and involves multiple stages, making it less efficient. To address these issues, a more streamlined one-pot synthesis method for the *O*-acylation of CS in MeSO_3_H has recently been developed. This new procedure simplifies the process by reducing the number of synthetic steps, thus enhancing the efficiency and making it more practical for large-scale applications [27].

The properties, biological activity, and methods of preparation of acylated CS derivatives are illustrated in Table 2.

#### 3.2.1. Succinylation

**Figure 9:**
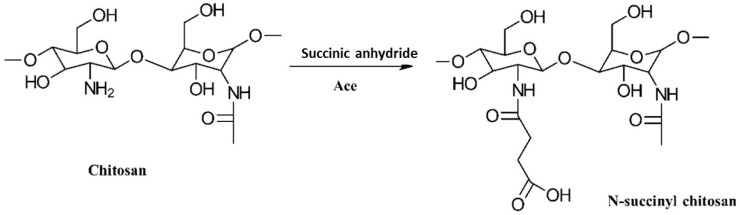
Synthesis of *N*-succinylated CS [29].

The succinylation of CS is a type of *N*-acylation where carboxyacyl groups are introduced by reacting with cyclic anhydrides. Specifically, the reaction of succinic anhydride with CS in dimethyl sulfoxide or acetic acid/methanol yields *N*-succinyl chitosan (NSC) with a controlled degree of succinylation [29]. For instance, Aiping et al. [30] synthesized NSC by first dissolving CS in 1 wt% acetic acid to achieve a 0.5% CS solution. They then dissolved succinic anhydride in acetone to produce a 1% solution, which was added dropwise to the CS solution over 30 min at room temperature. The reaction mixture was then allowed to react for 4 h at 40 °C. The reaction scheme is illustrated in Figure 9 [29].

After cooling the reaction mixture to room temperature, it was precipitated by adding excess acetone. The mixture was then filtered to separate the solvent and washed sequentially with 70%, 80%, and 100% acetone. The final product was dried under a vacuum at 40 °C for 24 h, resulting in the NSC derivative as a white powder [30].

**Figure 10:**
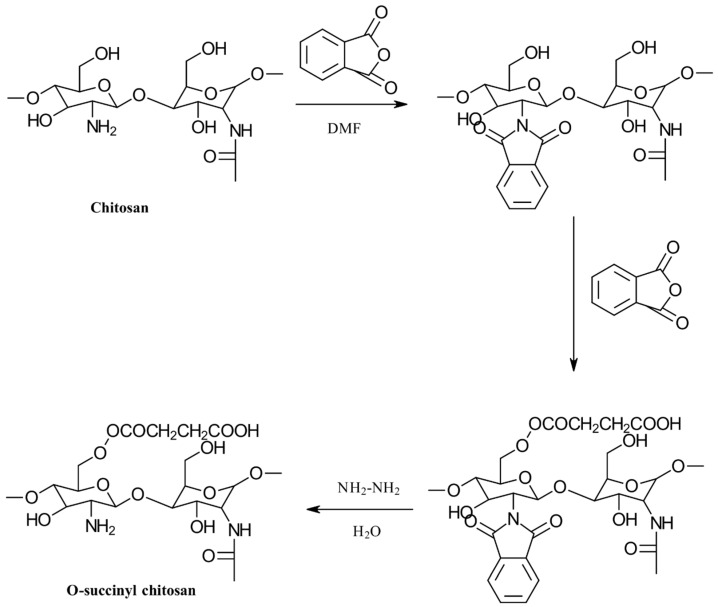
Synthesis of *O*-succinylated CS [31].

*O*-succinyl chitosan (OSC) is another notable succinyl derivative of CS, with properties akin to those of NSC. Since the amino groups in CS are more reactive than the hydroxyl groups, the formation of NSC is generally more common than that of OSC. Zhang et al. developed a water-soluble OSC by first protecting the highly hydrophilic amino groups with phthaloyl groups in DMF as the solvent. Following this, succinyl groups were introduced, and the phthaloyl groups were removed using hydrazine hydrate. The synthesis procedure is depicted in Figure 10 [31].

The properties, biological activity, and methods of preparation of succinylated CS derivatives are illustrated in Table 3.

#### 3.2.2. Benzoylation

**Figure 11:**
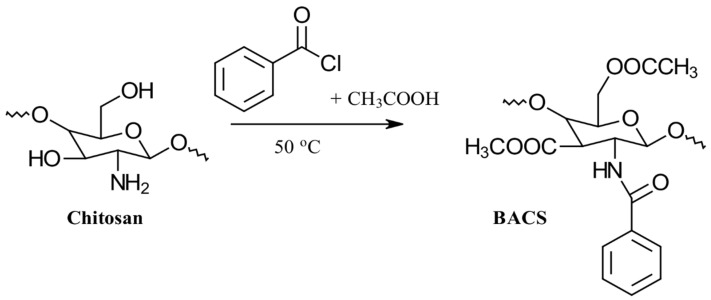
Reaction for preparation of *N*-benzoyl-*O*-acetyl-chitosan (BACS) [35].

Modifying CS with benzoic acid can effectively reduce the amount of benzoic acid needed in preparations, expand the antimicrobial spectrum, and enhance the amphipathic properties of the chitosan derivative. Cai et al. pioneered an ultrasonic-assisted method to synthesize *N*-benzoyl-*O*-acetyl-chitosan (BACS). In this method, the simultaneous catalytic action of benzoyl chloride and acetic acid introduces both benzoyl and acetyl groups into CS, resulting in the BACS derivative. The preparation scheme for benzoyl CS is illustrated in Figure 11 [35,36].

In another study, Sabarudin et al. chemically functionalized CS with a 3-nitro-4-amino benzoic acid moiety. This modified biopolymer was then used for the collection and concentration of trace amounts of molybdenum. The modification process involved attaching the carboxyl group of 3-nitro-4-amino benzoic acid to the -NH_2_ groups of CS through an amide linkage [37].

In a different method, the direct benzoylation of the two hydroxyl groups on CS was achieved using a phosphoryl-mixed anhydride system. This system, which included trifluoroacetic anhydride, benzoic acids, and phosphoric acid, allowed for the reaction to proceed as a one-pot process under mild conditions, eliminating the need for an inert atmosphere or dry solvents [38].

The properties, biological activity, and methods of preparation of benzoylated CS derivatives are illustrated in Table 4.

#### 3.2.3. Phthaloylation

**Figure 12:**
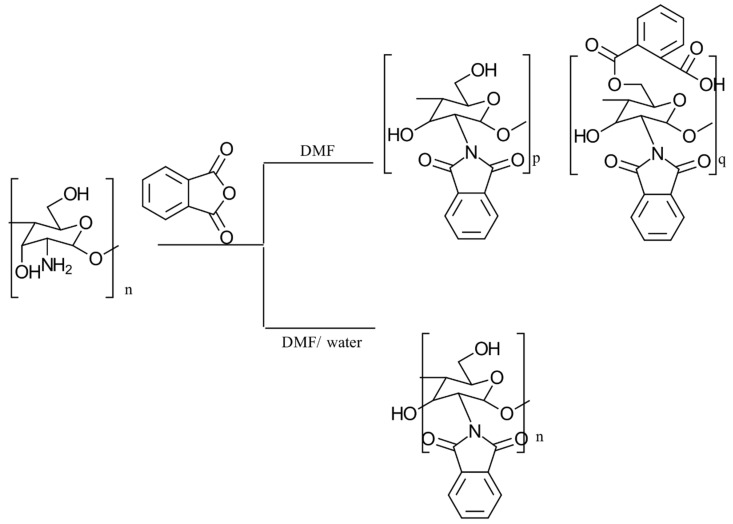
Synthesis of *N*-phthaloylated CS [40].

In the traditional *N*-phthaloylation of CS, the reaction is typically conducted in *N*,*N*-dimethylformamide. Kurita’s work [40] revealed that this reaction also led to the partial phthaloylation of the hydroxyl groups. By adding a small number of hydroxyl-containing compounds as chemoselective agents, *O*-phthaloylation was effectively suppressed. Among the tested compounds, water was particularly effective, allowing for the selective *N*-phthaloylation of CS without significant *O*-phthaloylation. The resulting *N*-phthaloyl-chitosan was crystalline despite the bulky substituent, and solubility tests showed that it had a strong affinity for organic solvents. This synthesis process is depicted in Figure 12.

The properties, biological activity, and methods of preparation of phthaloylated CS derivatives are illustrated in Table 5.

#### 3.2.4. Thiolation

The synthesis of thiolated CS can be broadly categorized into three types based on which functional groups react with the thiol reagent: hydroxyl groups, amino groups, or both. These methods can be further divided into two main approaches: direct substitution or interaction with an SH-containing ligand. For a thorough overview of these synthesis techniques, see the reviews by Alkabli [44] and Federer et al. [45].

Thiolation is a method used to functionalize various polymers, including CS, by introducing thiol groups using thiolating agents. These agents include cysteine, thioglycolic acid (TGA), 2-iminothiolane, 4-thiobutylamidine (TBA), *N*-acetyl cysteine, isopropyl-S-acetylthioacetimidate, and glutathione [46]. This technique, initially pioneered by Bernkop-Schnürch and colleagues [47,48], is aimed at improving the mucoadhesion properties of polymers for use in pharmaceutical and biomedical applications.

**Figure 13:**
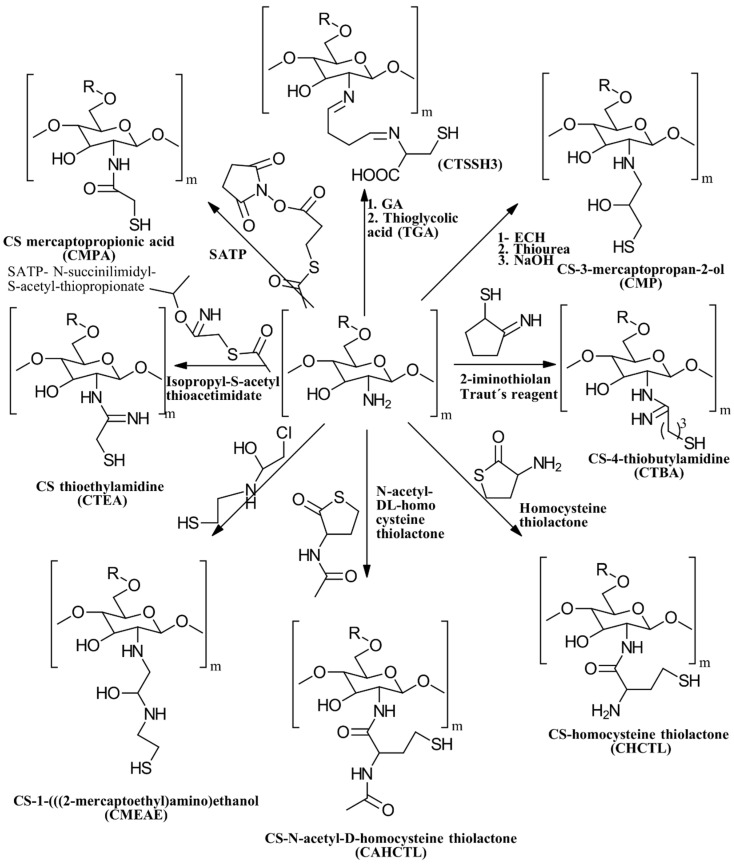
Synthesis of *N*-thiolated CS [44].

**Figure 14:**
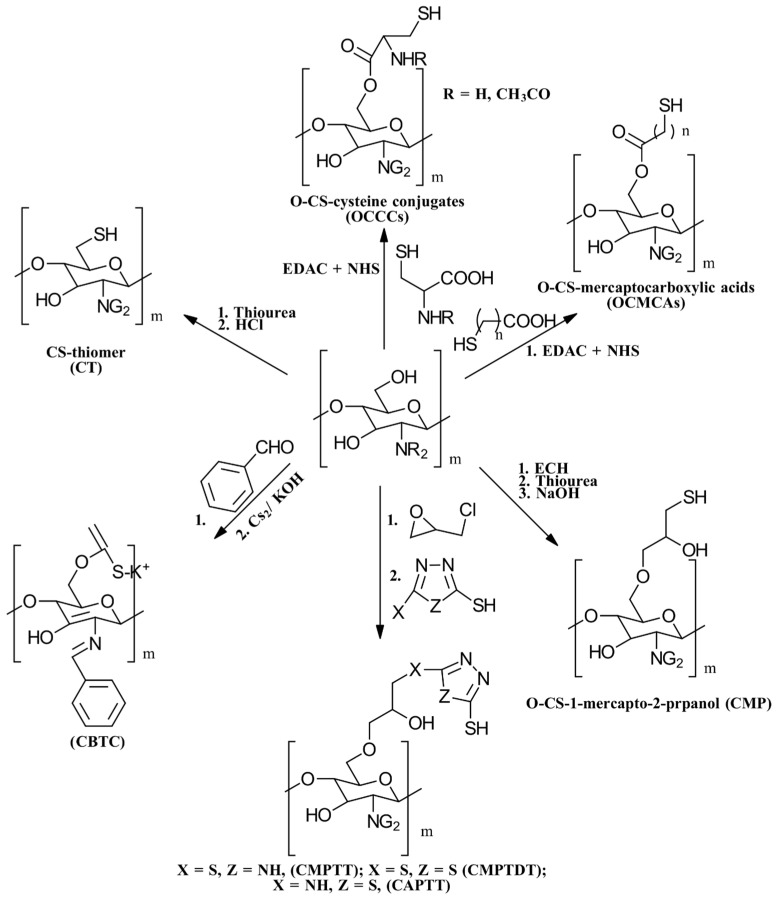
Synthesis of *O*-thiolated CS.

**Figure 15:**
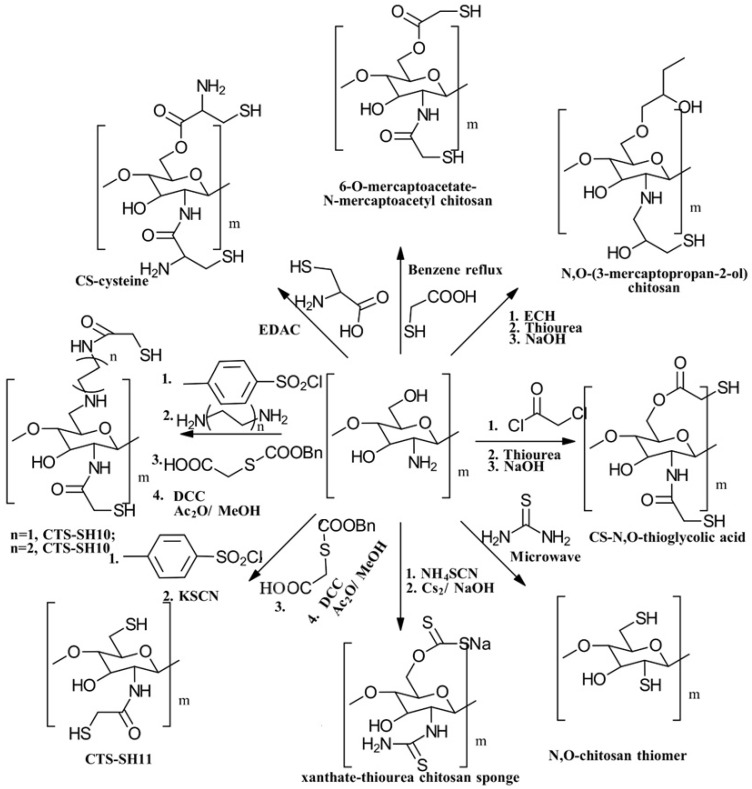
Synthesis of *N*,*O*-thiolated CS [44].

(i) *N*-thiolation, or the thiolation of the NH_2_ groups on the CS backbone, can be achieved through several methods. One common approach involves coupling the NH_2_ groups of CS with the COOH groups of mercaptocarboxylic acid derivatives, a reaction that is catalyzed by a water-soluble carbodiimide such as EDAC to activate the COOH groups for coupling [49]. Another method involves the nucleophilic attack of CS NH_2_ groups by bis-electrophiles like epichlorohydrin or chloroacetyl chloride to produce *N*-chloro-CS derivatives. These derivatives can then be converted into thiol derivatives using a thiourea–NaOH mixture or aminothiol reagents. Additionally, reactive thiol-containing reagents such as 2-iminothiolane (Traut’s reagent), thiolactones, *N*-hydroxysuccinimide esters, and acetimidates can react with the primary amine groups on CS through nucleophilic ring-opening reactions to introduce sulfhydryl groups [50]. Lastly, cysteine and other amino thiol derivatives can be grafted onto the CS backbone via dual Schiff base condensation reactions, using GA as a crosslinker [51]. Figure 13 illustrates the synthesis of *N*-thiolated CS.
(ii) *O*-thiolation involves the addition of thiol or sulfhydryl groups to the primary hydroxyl groups on the CS backbone. To perform *O*-thiolation, the NH_2_ groups must first be protected through alkylation or Schiff base condensation reactions. The various strategies for *O*-thiolation are depicted in Figure 14 [44].
(iii) *N*,*O*-thiolation involves covalently attaching thiol-tagging reagents to CS by forming amide bonds with NH_2_ groups and ester bonds with primary OH groups on the CS backbone. The most common method for *N*,*O*-thiolation uses pre-activated mercaptocarboxylic acid derivatives, such as L-cysteine (Cys) and thioglycolic acid (TGA), coupled with the amino and hydroxyl groups of CS. This process is typically facilitated by an EDAC coupling agent. Figure 15 illustrates the various strategies used to achieve *N*,*O*-thiolation [44,50].

The properties, biological activity, and methods of preparation of thiolated CS derivatives are illustrated in Table 6.

#### 3.2.5. PEGylation

**Figure 16:**
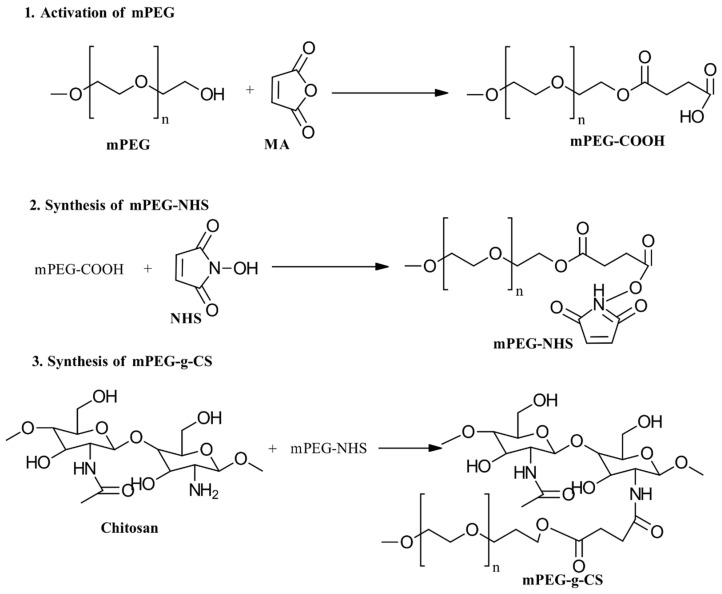
Synthesis of PEGylated CS [53].

To synthesize PEGylated CS, functionalizing PEG with an appropriate end group is crucial, as shown in Figure 16 [53]. One effective approach is chemically modifying commercial PEG. Specifically, modifying the end group of α-monomethoxy ω-hydroxy-PEG creates a reactive PEG intermediate that is suitable for grafting onto CS. Various PEGylated CS products have been synthesized using different chemical modifications and PEG derivatives, such as PEG-aldehyde, PEG-carboxylic acid, PEG-carbonate, PEG-iodide, PEG-epoxide, PEG-acrylate, PEG-NHS ester, and PEG-sulfonate [54]. Most studies have focused on using methoxy-poly(ethylene glycol) (mPEG) rather than poly(ethylene glycol) (PEG) for the PEGylation of CS to prevent polymer crosslinking. In this method, the amino group of CS reacts with the active end of mPEG, while the opposite end, blocked by a methoxy group, prevents further reactions with other amines. Despite these advancements, some PEGylation reactions for PEG-g-CS can be labor-intensive and yield small amounts, which limits their scalability and routine production [55,56].

The properties, biological activity, and methods of preparation of PEGylated CS derivatives are illustrated in Table 7.

#### 3.2.6. Attachment of Steroids

**Figure 17:**
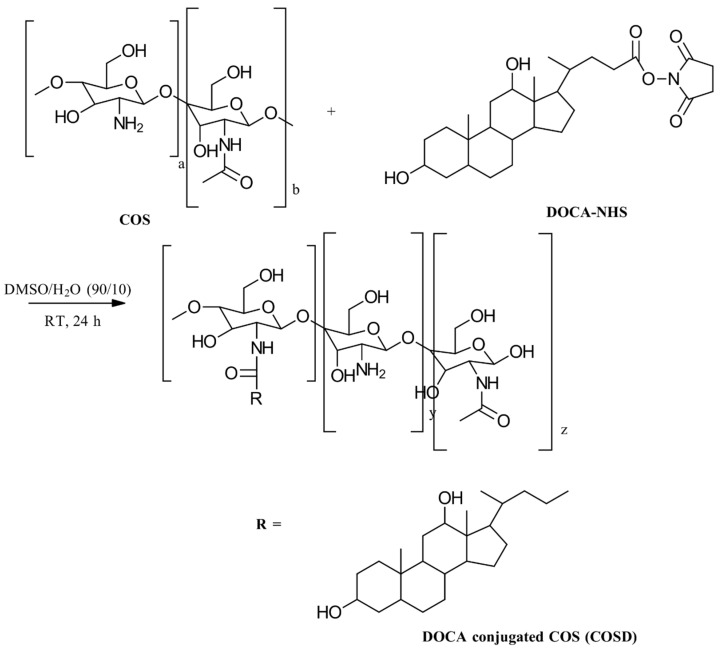
Preparation of CS derivative with deoxycholic acid (COS = chitosan oligosaccharide; DOCA-NHS = deoxycholic acid–*N*-hydroxysuccinimide) [59].

Steroids can endow CS with amphiphilic properties and are commonly used for hydrophobic modification. The key steroids employed in this modification include 5β-cholanic acid, cholic acid, and cholesterol. The synthesis of steroid-modified CS involves activating the carboxylic acid group with *N*-hydroxysuccinimide (NHS) and then grafting it onto the primary amine of glycol CS. This grafting process is facilitated using EDC as a mediator, as illustrated in Figure 17 [59].

Recently, Ding et al. [60] developed a method for the conjugation of cholic acid with CS using a precipitation technique. They began by preparing a 1% solution of CS in a DMSO/H₂O mixture (10:1). To this solution, they added *N*-hydroxysuccinimidoyl cholic ester, synthesized from cholic acid in tetrahydrofuran. The reaction mixture was stirred for 48 h at room temperature. Following this, the solution was precipitated by introducing an excess of acetone/methanol (2:1). The resulting precipitates were collected via centrifugation, washed with acetone, and dried under a vacuum. The solid was then dissolved in distilled water and dialyzed against deionized water using 3000 Da dialysis bags for 72 h. After dialysis, the solution was filtered through a syringe filter with a pore size of 1.2 μm to remove larger aggregates. The final product, cholic-acid-conjugated CS, was obtained by lyophilization as a solid.

#### 3.2.7. Complexation with EDTA

**Figure 18:**
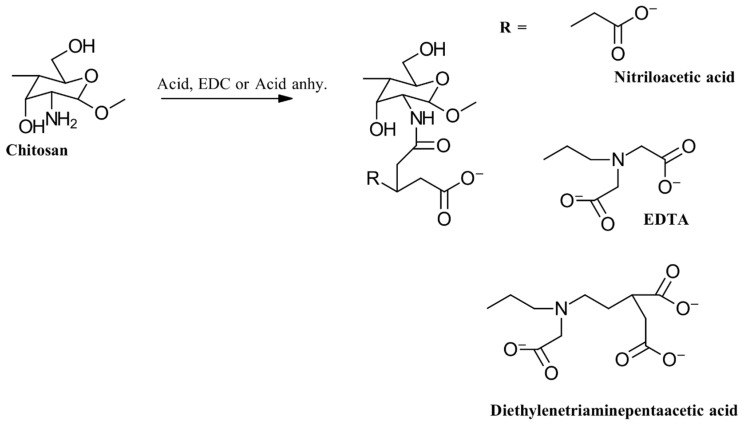
Synthesis of CS–EDTA derivative [61].

The derivatization of CS can improve its ability to complex with metal ions and enhance its metallopeptidase-inhibiting properties. Thus far, chitosan conjugates with nitrilotriacetic acid (NTA) and diethylenetriaminepentaacetic acid (DTPA) have been synthesized using carbodiimide chemistry or by reacting anhydrides of these complexing agents. The synthesis process is illustrated in Figure 18 [61].

The modification of the amino groups in CS with DTPA showed that only about 64% of the amino groups were successfully altered. This limitation might be due to steric hindrance from the previously attached DTPA, which can obstruct the further linkage of additional DTPA molecules to the primary amino groups of the polymer. In contrast, CS–EDTA conjugates, which involve the quantitative modification of all primary amino groups, do not exhibit this issue [62,63].

The CS–EDTA conjugate has been explored for various modifications and applications. Loretz et al. [63] adapted a method from Bernkop-Schnürch et al. [64] to synthesize CS–EDTA conjugates. They began by preparing a 1% CS suspension in demineralized water, adjusting the pH to 3 with 1 M HCl until the polymer dissolved completely. After adding additional demineralized water, the solution was adjusted to a 1% CS concentration. Increasing amounts of EDTA were then introduced into aliquots of this solution, with the pH adjusted to 6.0 using 5 N NaOH. To facilitate the formation of amide bonds between the amino groups of CS and the carboxyl groups of EDTA, 0.1 M 1-ethyl-3-(3-dimethylaminopropyl)carbodiimide hydrochloride (EDAC) was added. The reaction was incubated at room temperature with constant stirring for 12 h. The resulting conjugates were isolated by extensive dialysis against demineralized water and 0.05 N NaOH and then lyophilized and stored at room temperature. Additionally, S-protected thiolated CS–EDTA has been synthesized to leverage the benefits of EDTA, thiolation, and thiol group protection or activation.

The properties, biological activity, and methods of preparation of CS–EDTA derivatives are illustrated in Table 8.

#### 3.2.8. Complexation with Cyclodextrins

Solutions of chitosan-grafted cyclodextrin derivatives (CS-g-CDs) can form complex structures through both intramolecular and intermolecular interactions. These interactions may cause significant increases in viscosity or result in temporary and reversible supramolecular network systems. Effective control over the grafting process is essential in regulating the molecular architecture, which directly impacts the polymer’s behavior and properties. Various chemical modification techniques used for CS can also be applied to graft cyclodextrin. The main procedures used to achieve this are detailed in the following sections.

In 2001, Auzély-Velty and Rinaudo [66] employed a reductive amination technique to graft cyclodextrin (CD) onto CS. They reacted a CS solution in acetic acid/methanol with an aldehyde-modified CD derivative, using sodium cyanoborohydride (NaCNBH_3_) for reduction and EDAC as the coupling agent. This process involved initially forming a Schiff base between the aldehyde group of the CD and the amino groups of CS, followed by reduction to secure the grafting. This method is favored for its simplicity, ease of use, and minimal degradation of the polymer [67,68].

The second major method of grafting cyclodextrin (CD) onto CS is amidation, which involves reacting carboxyl-modified CDs with the amino groups of CS. There are two primary strategies for this process: (i) the traditional condensation reaction [69] and (ii) amidation using coupling activators such as 1-ethyl-3-(3-dimethylamino)propyl carbodiimide/*N*-hydroxysuccinimide (EDC/NHS) [70]. The condensation reaction typically requires high temperatures due to its high activation energy. In contrast, coupling agents like EDC/NHS facilitate the formation of amide bonds under milder and more controlled conditions in aqueous solutions [69].

The third method of grafting cyclodextrins (CDs) onto the CS backbone is nucleophilic substitution, where halides or tosyl groups are replaced by the amino groups of CS [69]. Chen and Wang [71] demonstrated this approach using tosylated β-CD, assessing its potential for the in vivo release of iodine-131 and enhanced solubility.

**Figure 19:**
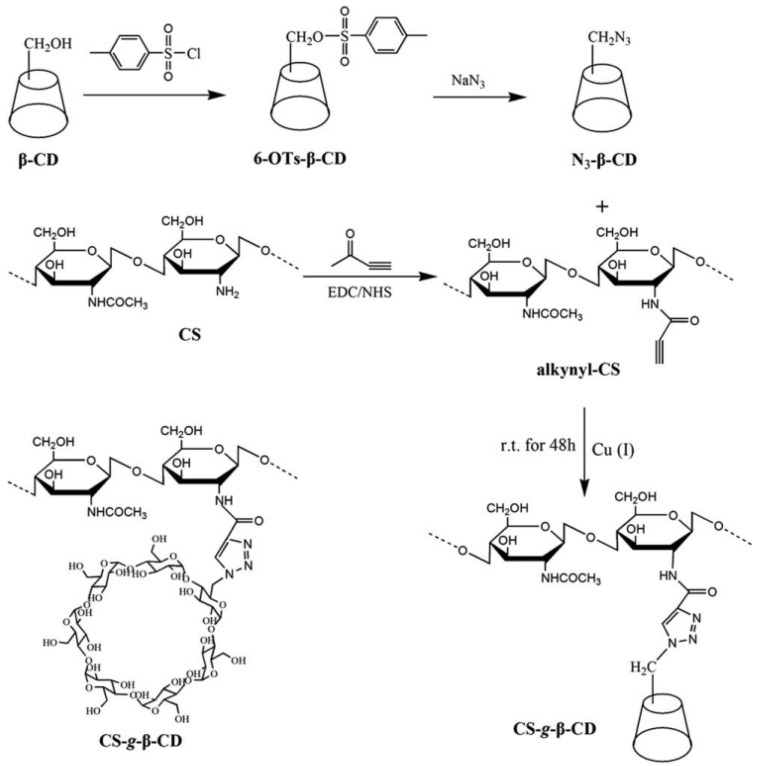
Synthesis route for CS-g-β-CD [70].

The fourth approach for the attachment of β-cyclodextrin (β-CD) to CS utilizes click chemistry, as depicted in Figure 19 [70]. This technique involves the Huisgen cycloaddition reaction, which allows for the precise grafting of β-CD onto the CS backbone. This reaction can target either the amino group (position 2) or the O-6 position on the β-CD [69,71]. Additionally, alternative methods include (i) preparing epoxy-activated CS and reacting it with the hydroxyl groups of β-CD and (ii) using 1,6-hexamethylene diisocyanate to facilitate the attachment of β-CD to CS [69].

The properties, biological activity, and methods of preparation of CS–CD derivatives are illustrated in Table 9.

#### 3.2.9. Crown-Ether-Linked CS

**Figure 20:**
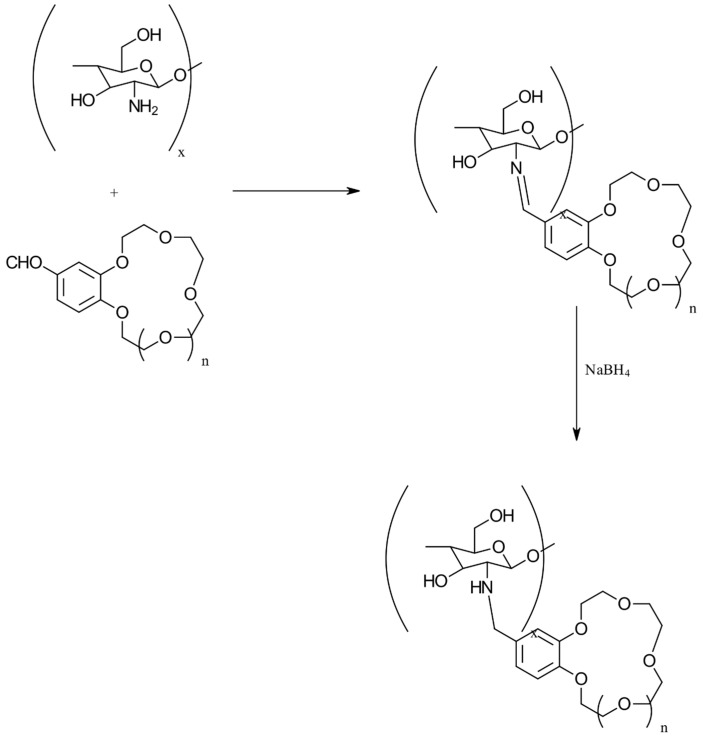
A crown ether–chitosan (CS) derivative is synthesized through a Schiff’s base reaction. In this process, the crown ether is activated and then reacts with the amino groups of CS to form a Schiff base linkage. This reaction enhances the metal-binding properties of the chitosan.

Crown ethers are known for their high selectivity in binding metal ions due to their unique molecular structures. To create crown-ether-functionalized CS, a Schiff base reaction was employed (Figure 20) [73]. This method involves the reaction of a crown ether with CS to form a Schiff base linkage, integrating the crown ether’s metal-binding properties into the CS framework.

*N*-benzylidene chitosan (CNB), a derivative of chitosan, was initially synthesized through a reaction involving chitosan and benzaldehyde by Wan et al. [74]. Building upon this foundation, the same researchers produced chitosan-dibenzo-18-crown-6 derivatives, designated as CNBD and CND, by engaging 4,4-dibromodibenzo-18-crown-6 in reactions with CNB and CTN, respectively. In parallel research, Peng et al. [75] introduced a novel approach by synthesizing Schiff-base-type chitosan–crown ether resins, specifically CTS-B15 and CTS-B18, through the condensation reaction between chitosan’s primary amino groups and benzaldehyde derivatives. Expanding on the concept of chitosan–crown ether composites, the creation of chemically stable and porous aza-crown-ether-crosslinked chitosan films was achieved by reacting chitosan with varying quantities of *N*,*N*-diallyl-7,16-diaza-1,4,10,13-tetraoxa-dibenzo-18-crown-6. Notably, an X-ray diffraction analysis revealed the progressive disruption of the crystalline structure within the chitosan polymer chain as the proportion of grafted aza-crown ether increased.

The properties, biological activity, and methods of preparation of crown ether–CS derivatives are illustrated in Table 10.

### 3.3. Other Covalent-Based Synthetic Approaches

Among the various methods of modifying chitosan, esterification is the most common approach. This process involves the reaction of the hydroxyl groups on chitosan with oxygen-containing mineral acids or their derivatives to produce esterified chitosan. Esterification enhances the adsorption and antibacterial properties of chitosan to varying degrees. Common esterification reactions include sulfation, sulfonation, and phosphorylation. It is important to note that, while often targeting hydroxyl groups, these reactions can also modify chitosan’s amine groups.

#### 3.3.1. Sulfation and Sulfonation

**Figure 21:**
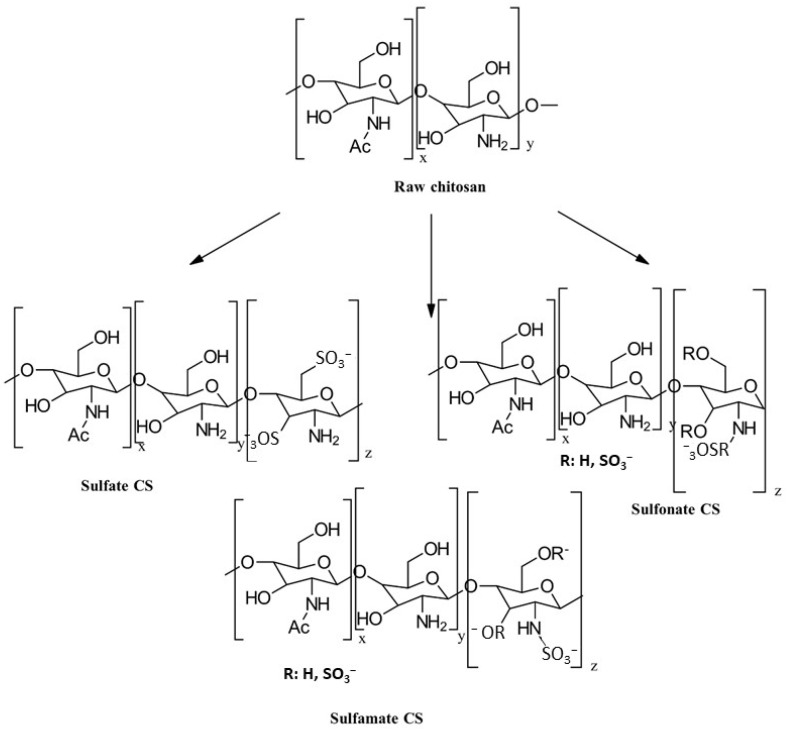
There are four primary strategies for the chemical modification of chitosan (CS) through sulfation and sulfonation: (1) selective *O*-position substitution after *N*-protection/deprotection, (2) non-selective substitution in *N*- and *O*-positions, (3) selective *N*-position substitution, and (4) selective *O*-position substitution without *N*-protection/deprotection [76].

The synthesis of sulfonated CS derivatives has been extensively studied to enhance the biological properties of raw CS. Although amino groups are known to be more nucleophilic than hydroxyl groups, chemical substitution can occur at three key positions within the glucosamine and acetyl glucosamine units: the C-2 amino group, the C-3 secondary hydroxyl group, and the C-6 primary hydroxyl group. These substitution sites are illustrated for sulfation and sulfonation in Figure 21 [76].

The chemical modification of chitosan (CS) through sulfation and sulfonation can yield a variety of derivatives, including those with modifications of the amino groups (*N*-modified), hydroxyl groups (*O*-modified), or both (*N*,*O*-substituted). The specific outcome is determined by the chosen reaction conditions and reagents. This chemical process typically involves one or more steps. Both the amino and hydroxyl functional groups within CS are susceptible to reaction with electrophilic agents, such as alkyl halides, acids, or isothiocyanates. The versatility of these functional groups allows for a wide range of modification possibilities. Several research groups have explored the synthesis of sulfonated CS. For example, Rwei and Tsai [77,78] developed a method to produce sulfonated CS through *N*-substitution using 1,3-propane sultone. In another approach, Zhang et al. [79] synthesized sulfonated CS by treating CS with concentrated sulfuric acid. These methods generally involve dissolving CS in an acidic solution, introducing the sulfonating agent under controlled conditions, precipitating the product, and subsequently purifying it through washing and drying processes.

Chlorosulfonic acid (HClSO_3_) in combination with dimethylformamide (DMF) is a commonly used method for the introduction of sulfonate groups to the hydroxyl groups of CS through *O*-substitution [80]. Han et al. [81] investigated different substitution positions and prepared 6-*O*-sulfated CS using HClSO_3_/H_2_SO_4_, 3,6-*O*-disulfated CS using HClSO_3_/DMF, and 3-*O*-sulfated CS through the desulfation of 3,6-*O*-disulfated CS.

Yang et al. [82] synthesized *N*,*O*-sulfated CS through *N*,*O*-substitution using trimethylsilylated CS and a sulfur trioxide–pyridine complex in anhydrous DMSO. Additionally, Wang et al. [83] prepared *N*-succinyl CS sulfates by reacting *N*-succinyl CS with the sulfating agent N(SO_3_Na)_3_.

The properties, biological activity, and methods of preparation of sulfonated CS derivatives are illustrated in Table 11.

#### 3.3.2. Phosphorylation

**Figure 22:**
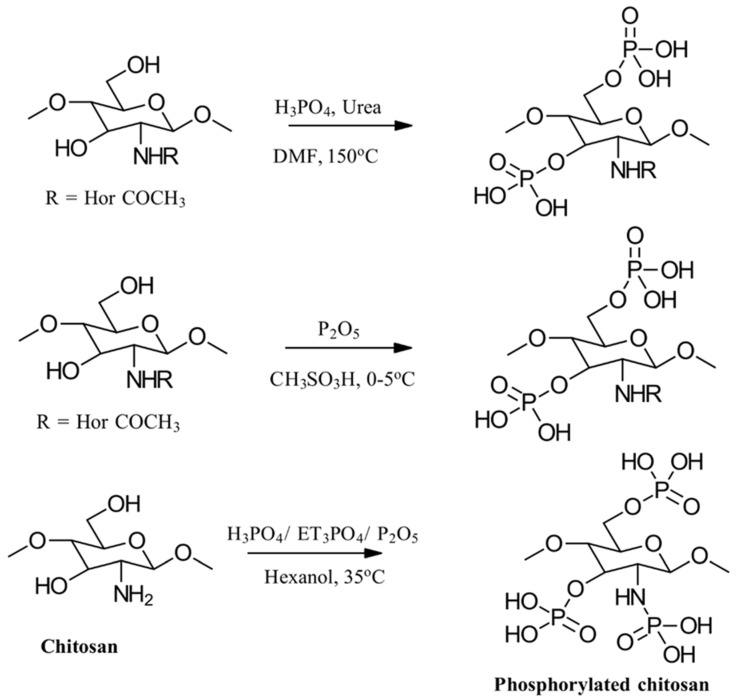
Examples of three possible phosphorylation reactions used to prepare phosphorylated CS [86].

To enhance chitosan’s properties, researchers have also focused on incorporating phosphate groups through a process known as phosphorylation. This involves chemically modifying chitosan’s hydroxyl groups to create phosphonate derivatives. Several methods have been employed, with three primary approaches: using H_3_PO_4_/urea, H_3_PO_4_/Et_3_PO_4_/P_2_O_5_, or P_2_O_5_/methanesulfonic acid as reaction environments. These methods have shown promise in creating modified chitosan materials with potential for various applications [86,87,88,89]. Examples of three possible phosphorylation reactions used to prepare phosphorylated CS are shown in Figure 22.

**Figure 23:**
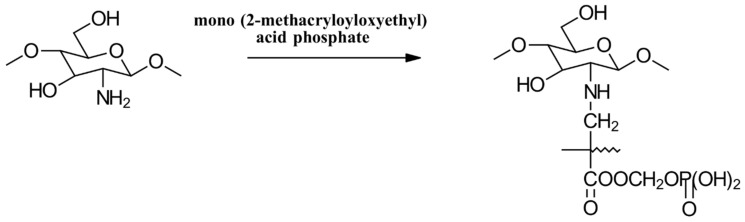
Synthesis of phosphorylated chitosan by grafting method [86].

In addition to the phosphorylation methods mentioned, PCS derivatives can also be synthesized through alkylation reactions (Figure 23). For instance, mono-(2-methacryloyloxyethyl) phosphoric acid has been used in a grafting copolymerization reaction to produce a PCS product with zwitterionic properties and enhanced antimicrobial activity [86].

Additional CS derivatives include *N*-methylene phosphonic CS, synthesized using phosphorous acid and formaldehyde, and chitosan-*O*-ethyl phosphonate, prepared using KOH/methanol and 2-chloro ethyl phosphonic acid [86].

The properties, biological activity, and methods of preparation of phosphorylated CS derivatives are illustrated in Table 12.

#### 3.3.3. Methacrylation

**Figure 24:**
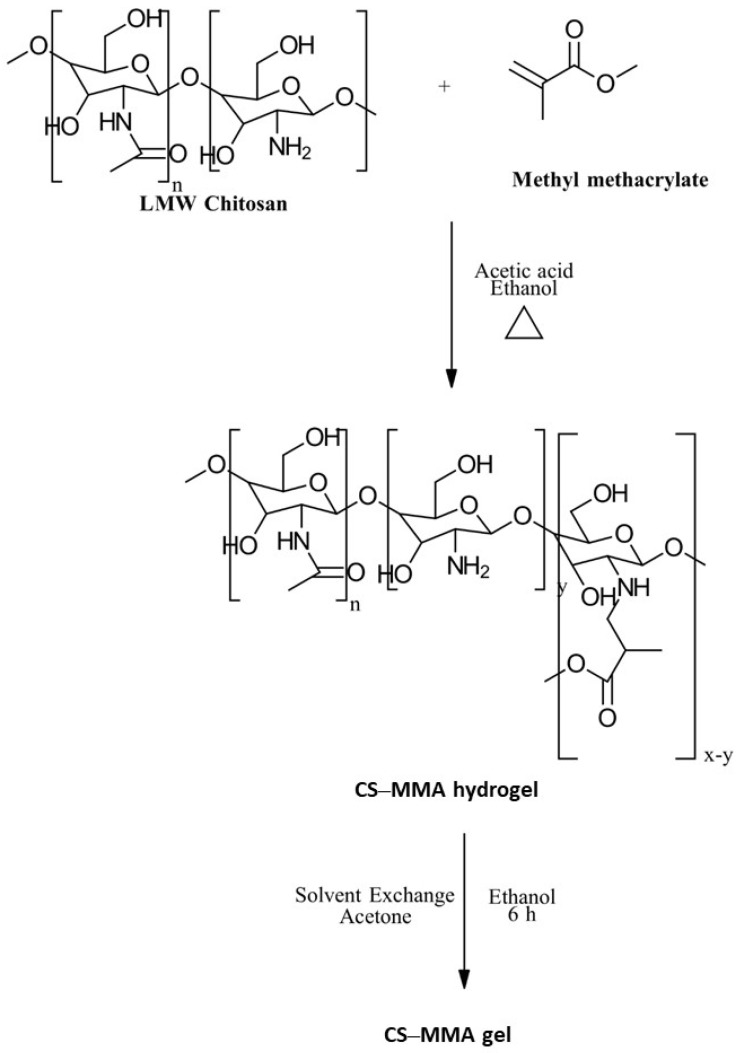
Preparation of CS–MMA derivative [97].

To broaden the functionality of CS, researchers have explored its conjugation with methyl methacrylate (MMA). This combination aims to impart desirable properties to the resulting biopolymer, opening up potential applications in various fields. A common approach involves the chemical modification of CS through a reaction with methacrylic anhydride, as demonstrated by Kolawole et al. [94], to enhance properties such as mucoadhesion. To create CS–MMA copolymers, the graft polymerization of the vinyl monomer onto the CS backbone is a frequently employed method. This process typically utilizes free radical initiation [95]. Kumar et al. [96] successfully synthesized CS–MMA by reacting CS with MMA in an aqueous ethanol solution under specific conditions. However, a more environmentally friendly and economically viable approach was introduced by Jaiswal et al. [97]. Their method involved a Michael addition reaction in ethanol, eliminating the need for harmful free radical initiators (Figure 24).

The properties, biological activity, and methods of preparation of methacrylated CS derivatives are illustrated in Table 13.

### 3.4. Crosslinking Methods for Preparation of CS Derivatives

#### 3.4.1. Chemical Crosslinking

**Figure 25:**
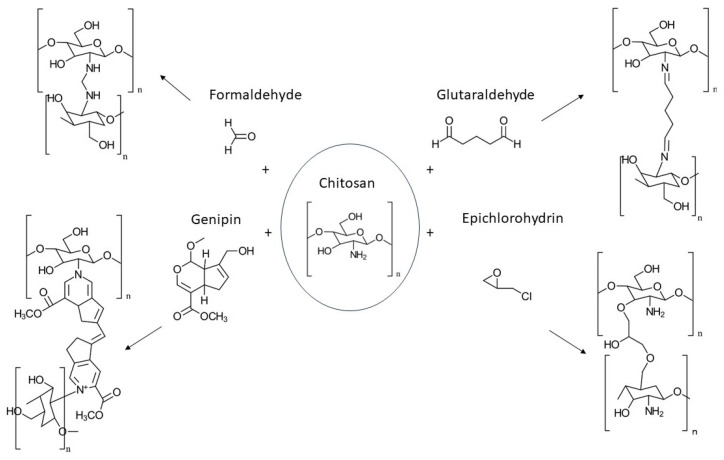
To enhance CS’s stiffness and strength, covalent crosslinking using agents such as formaldehyde, glutaraldehyde, epichlorohydrin, and natural alternatives like genipin are commonly employed.

To enhance CS’s stiffness (Young’s modulus) and strength (tensile strength), covalent crosslinking is often employed. A variety of crosslinking agents can be used, including both natural options like vanillin and genipin, as well as synthetic alternatives such as formaldehyde, glutaraldehyde (GA), epichlorohydrin, oxidized dextran, oxidized starch, oxidized sugars, and poly(ethylene glycol) diglycidyl ether (PEGDGE). Formaldehyde, glyoxal, and GA are the most commonly used among these. The chemical crosslinking process involving various agents is illustrated in Figure 25 [98,99,100,101,102,103].

GA has been widely used to crosslink CS and enhance its mechanical properties. GA can be added directly to CS solutions or applied to preformed CS gels. The crosslinking process occurs under acidic or alkaline conditions through the formation of imine bonds between CS’s amino groups and GA’s carbonyl groups [98,101,104].

Genipin, a naturally derived crosslinking agent obtained from Gardenia fruit, has gained significant attention for its ability to modify CS. As a water-soluble compound, genipin readily reacts with CS to form stable, blue-colored fluorescent hydrogels [105]. The crosslinking mechanism is pH-dependent. Under acidic or neutral conditions, CS’s amino groups initiate a nucleophilic attack on genipin’s olefinic carbon, leading to subsequent reactions and crosslink formation. Conversely, under alkaline conditions, hydroxyl ions induce the ring opening of genipin, followed by aldol condensation and Schiff base formation with CS’s amino groups [102,105].

#### 3.4.2. Physical Crosslinking

Physical CS gels offer advantages such as lower toxicity and the ability to control properties like swelling and degradation. However, their mechanical strength is generally lower compared to chemically crosslinked gels. These gels form through a combination of electrostatic, hydrophobic, and hydrogen-bonding interactions, influenced by factors like the temperature, pH, and ionic strength. As a polyelectrolyte, CS interacts with ions, forming a gel structure within a pH range of 4–6 [106].

**Figure 26:**
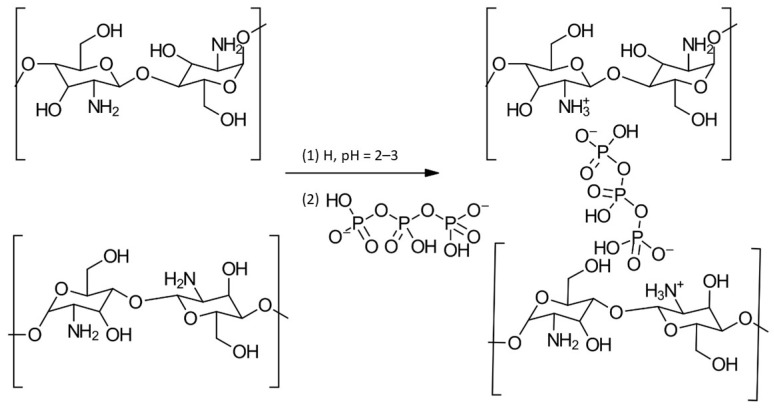
Scheme of CS–TPP ionic crosslinking [107].

Polycationic CS, soluble in acidic media (pKa 6.5), interacts with negatively charged tripolyphosphate (TPP). This interaction, driven by electrostatic attraction between CS’s amino groups and TPP, forms an intermolecular or intramolecular network structure, influenced by the pH [107]. The reaction is shown in Figure 26.

The properties, biological activity, and methods of preparation of CS–TPP derivatives are illustrated in Table 14.

## 4. Physicochemical Properties, Biological Activity, and Use of CS Derivatives

CS derivatives are classified and discussed here according to their structural characteristics and resulting application potential and practical implementations, including alkylated CS (Section 4.1), namely carboxyalkylated CS (Section 4.1.1) and quaternized CS (Section 4.1.2); acylated CS (Section 4.2), namely succinyl CS (Section 4.2.1), benzoyl CS (Section 4.2.2), phthaloyl CS (Section 4.2.3), thiolated CS (Section 4.2.4), PEGylated CS (Section 4.2.5), CS linked with steroids (Section 4.2.6), CS–EDTA (Section 4.2.7), and cyclodextrin-grafted CS (Section 4.2.8), crown-ether linked with CS (Section 4.2.9); other covalently bound CS (Section 4.3), namely sulfonated CS (Section 4.3.1), phosphorylated CS (Section 4.3.2), methacrylated CS (Section 4.3.3), and glutaraldehyde and genipin crosslinked CS (Section 4.3.4); and physically crosslinked CS, (Section 4.4), namely ionically-crosslinked CS (Section 4.4.1) and non-ionically crosslinked CS (Section 4.4.2). The physicochemical properties and biological activity are discussed within these particular groups of CS derivatives, emphasizing their practical use/application potential, as summarized in Section 4.5: patents are described in Section 4.5.1, current clinical studies in Section 4.5.2., and challenges and future perspective of CS derivatives are discussed in Section 4.5.3.

### 4.1. Alkylated CS

Alkyl group introduction reduces intra- and intermolecular hydrogen bonding in CS, enhancing its water solubility and potential biomedical applications. However, the hydrophobic nature of the alkyl chain can decrease the solubility if the chain is too long, indicating that the alkyl chain length can control the solubility of CS derivatives [112,113].

*N*-alkylated CS demonstrated good biocompatibility in hemolysis and toxicity tests. Moreover, it exhibited superior hemostatic activity compared to unmodified CS in in vitro blood coagulation assays [16].

Alkylated CS demonstrates potential as a material for medical gauze due to its clotting and antibacterial properties. Additionally, its positive charge allows it to effectively absorb anionic surfactants, making it suitable for water purification applications [6,14].

Alkylated CS has shown significant potential in DNA delivery, particularly with dodecyl CS [113]. The improved transfection efficiency is attributed to enhanced cell entry through hydrophobic interactions and easier DNA release due to the weakened electrostatic attraction between the DNA and the carrier. Increasing the alkyl chain length to up to eight carbons further enhances the transfection efficiency, after which it plateaus [14].

#### 4.1.1. Carboxyalkylated CS

Modifying CS by carboxyalkylation leads to the formation of conjugates and complexes that are suitable for drug or gene therapy and targeted or controlled drug release. Negm et al. [112] highlighted the great potential of carboxyalkylated CS in medical applications due to its non-toxic, biocompatible, and excellent water-soluble properties. Among the water-soluble CS derivatives, amphiprotic ether derivatives, which contain -COOH groups and -NH_2_ groups in the molecule, have many other outstanding physicochemical and biological properties, including a variable pH-dependent ionic nature (cationic, neutral, anionic), antibacterial effects, and antifungal bioactivity. Due to these advantages, amphiprotic carboxyalkylated CS derivatives have received considerable attention in biomedical applications [114].

Carboxylated CS derivatives have been applied in controlled drug delivery. The carboxyl groups enhance the water solubility and mucoadhesive properties of CS, allowing for the steady and sustained release of drugs. This means that the drugs can remain at the application site for longer, increasing their therapeutic efficacy by ensuring sustained drug delivery. This modification is beneficial for oral and topical drug delivery applications [114].

*N*-carboxymethyl CS has been used with timolol maleate (an anti-glaucoma drug) [115].

Carboxymethyl CS is applied in gene delivery systems. To overcome the biological barriers at all levels and enhance the delivery efficiency of siRNA, Zhang et al. [116] prepared a multifunctional siRNA delivery system (carboxymethyl CS/siRNA nanoparticles) through the self-assembly of carboxymethyl CS modified with histidine, cholesterol, and anti-EGFR antibodies. This system could effectively silence vascular endothelial growth factor A (VEGFA) to cause cell apoptosis and inhibit proliferation. In vivo, it could target tumor sites to knock down VEGFA and achieve a better antitumor effect.

Carboxymethyl CS derivatives exhibit antibacterial and antifungal properties [23]. Carboxymethyl chitosan was found to have better antifungal activity when applied in gauze coated with it [117]. The carboxyl groups also increase cell adhesion and proliferation, supporting the regeneration of damaged tissue. This is beneficial for wound dressings and tissue engineering scaffolds. For example, Chang et al. [118] invented a new tape consisting of a biodegradable, injectable carboxymethyl CS-based hydrogel for accelerated diabetic wound healing. The dual-drug-loaded hydrogel consisted of *N*,*O*-carboxymethyl CS modified with heparin and carboxymethyl cellulose aldehyde. Cytotoxicity studies and histological assays demonstrated the excellent biocompatibility of the hydrogel. In vivo studies showed that the hydrogel could accelerate the progress of wound healing at the early stage of trauma, and re-epithelialization and collagen deposition were better than those in the control group. This biocompatible gel dressing could act as a drug delivery carrier to improve drug availability, promote cell migration and proliferation, decrease DNA damage, shorten the inflammatory period, and promote the formation of blood vessels and collagen fiber deposition in a diabetic skin injury model.

In recent years, many studies have been published concerning the use of carboxymethyl CS derivatives to fabricate different tissue engineering biomaterials, including hydrogels, scaffolds, nanoparticles, nanofibers, composites, dendrimers, and membranes [119]. For instance, Kebria et al. [120] developed a rapidly in situ-forming injectable oxidized carboxymethyl CS/pullulan hydrogel through crosslinking loaded with parathyroid hormone to enhance osteogenesis. This hydrogel was implanted into mandibular bone defects in Sprague-Dawley rats. The in vivo study revealed accelerated bone regeneration and angiogenesis, attributed to the rapid gelation rate and sustained release of parathyroid hormone. This in situ-forming hydrogel shows potential as a universal bone filler in clinical applications, enhancing osteoregeneration.

In summary, carboxylated CS derivatives promote tissue regeneration and wound healing; they can be used in controlled drug delivery and gene delivery.

#### 4.1.2. Quaternized CS

The quaternization of CS, involving the addition of methyl or larger alkyl groups with or without spacers, or modifying CS before quaternization, introduces a permanent positive charge [119]. This modification enhances the solubility across a wide pH range and often improves the antimicrobial properties, biocompatibility, and biodegradability [3].

Trimethyl chitosan (TMC) exhibits superior mucoadhesive, permeation enhancement, drug delivery, and DNA delivery properties compared to unmodified CS. TMC can be further modified or grafted to adjust properties such as its solubility, cytotoxicity, or cell recognition ability [119].

**Figure 27:**
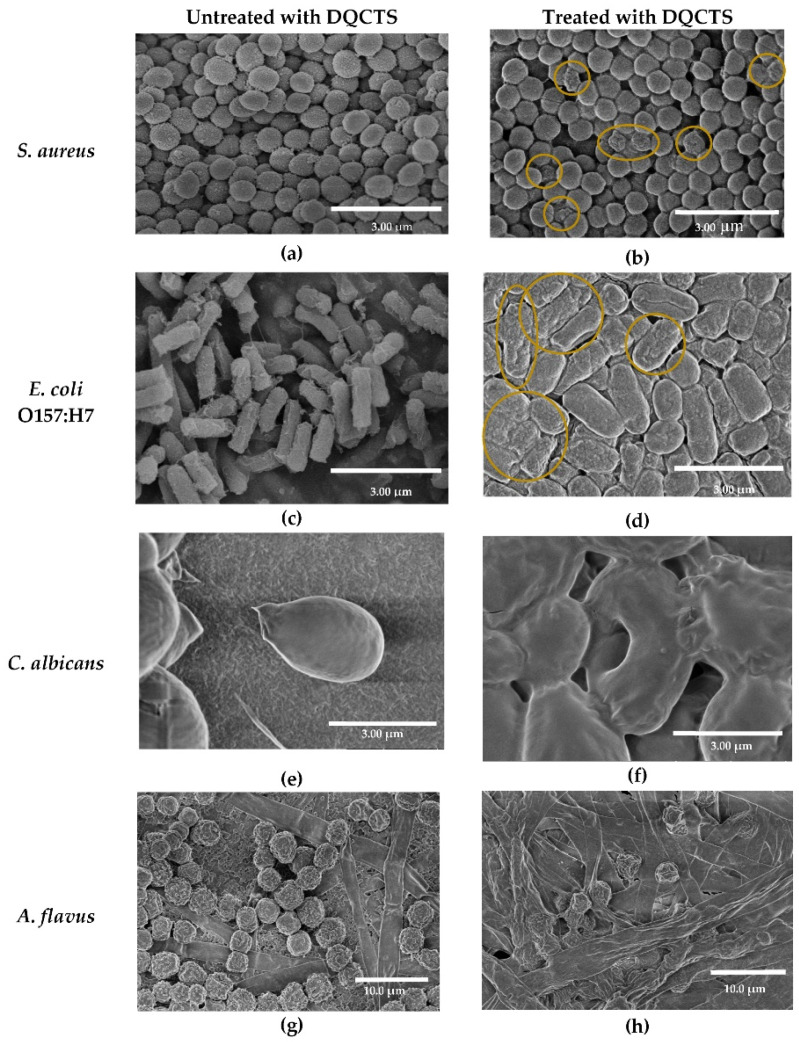
FE-SEM of microorganisms before (**a**,**c**,**e**,**g**) and after treatment (**b**,**d**,**f**,**h**) with DQCTS. Images reveal significant damage to the cell membranes of microorganisms treated with DQCTS (yellow circles), including cell shrinkage, surface disruption, and structural destruction, compared to intact control cells [121].

A notable approach to enhancing CS’s properties involves deacetylation and subsequent quaternization, as explored by Kim et al. [121]. This modification significantly improved the resulting CS derivative’s antimicrobial activity and water solubility. In particular, fully deacetylated and quaternized CS (DQCTS) exhibited superior antibacterial and antifungal efficacy against *Staphylococcus aureus*, *Escherichia coli*, *Candida albicans*, and *Aspergillus flavus* when compared to other CS derivatives, such as carboxymethyl chitosan and glycol chitosan [120]. This enhanced antimicrobial activity was attributed to the increased interaction of DQCTS with microbial cell membranes, disrupting their integrity. Furthermore, DQCTS demonstrated the ability to reduce the expression of virulence genes in bacteria and displayed low cytotoxicity against human skin cells. Scanning electron microscopy (SEM) analysis revealed pronounced damage to both bacteria and fungi cell membranes upon treatment with DQCTS, further emphasizing its potent antimicrobial properties (Figure 27).

Quaternized derivatives are being studied as components of new drug delivery systems [122,123]. Nezadi et al. [122] developed an injectable in situ-forming hydrogel, founded on quaternized CS, gelatin, and laponite, with the sustained release of the anti-inflammatory drug celecoxib for nucleus pulpous repair. Studies showed that the presence of Mg^2+^ ions and celecoxib in the hydrogel led to a suitable environment that encouraged glycosaminoglycan secretion. The formulated hydrogel is a promising candidate for minimally invasive degenerative disc repair.

CS can mediate the maturation of dendritic cells and drive the T helper type 1 (Th1)-biased immune response by the activation of cGAS and the STING signaling pathway, thereby promoting type I interferon production. Therefore, CS could be an excellent choice for combination with aluminum salts to improve the immunogenicity of vaccine antigens. Water-soluble quaternized CS is a better choice. To enable aluminum adjuvants to induce cellular immunity, composite nano-adjuvant *N*-2-hydroxypropyl trimethyl ammonium chloride chitosan nanoparticles (N-2-HACC-Al NPs) were synthesized by Liu et al. [124]. The N-2-HACC-Al NPs had good thermal stability, biodegradability, and lower cytotoxicity. To investigate the immunogenicity, a combined inactivated vaccine against Newcastle disease and H9N2 avian influenza was prepared with the N-2-HACC-Al NPs as a vaccine adjuvant. The immune effect of the vaccine was evaluated by chicken in vivo immunization. The vaccine induced higher levels of serum IgG, IL-4, and IFN-γ than the commercial combined inactivated vaccine against ND and H9N2 AI. The levels of IFN-γ were more than twice those of the commercial vaccine at 7 days post-immunization. These N-2-HACC-Al NPs could be used as an efficient nano-adjuvant to enhance the effectiveness of vaccines and have immense application potential.

Quaternized CS is also a suitable material for the design of novel wound dressings with favorable properties. Gao et al. [125] prepared a bilayered nanofibrous membrane (PQCQS) by electrospinning poly(ε-caprolactone) (PCL)/quaternized silicone (PQS, outer layer) and polyvinyl alcohol/collagen/quaternized chitosan (PCQC, inner layer) as wound dressings for damaged skin. The PQCQS membranes demonstrated a good balance between antibacterial activity and cell proliferation; in addition, they significantly expedited the wound-healing process and inhibited scar hyperplasia better than commercial ointments (MSSG and KELO-COTE) in a rabbit ear full-thickness wound defect model. They showed excellent antibacterial activity, hydrophilicity, hemostatic performance, scar inhibition, and wound-healing properties.

In tissue engineering, quaternized CS is used for the bone tissue engineering of scaffolds inspired by the natural bone structure. Shi et al. [126] designed a gradient-structural scaffold composed of functional biopolymers to provide an optimized 3D environment for the promotion of cell growth. To increase the interactions among the scaffolds, dopamine was used for alginate modification and a needle-like nano-hydroxyapatite was prepared with quaternized chitosan as a template. The scaffolds showed an appropriate degradation rate corresponding with the time of bone regeneration. Both human chondrocytes and fibroblasts could adhere to and grow well on the scaffolds in vitro. Furthermore, the excellent osteogenetic activity of the scaffold could effectively promote the regeneration of the bone tissue and accelerate the repair of the bone defects in vivo, compared with that of the scaffold with a homogenous structure.

In another study, Xu et al. [127] developed a tissue-specific extracellular matrix-based bioink and a 3D scaffold composed of a decellularized extracellular matrix, gelatin, and quaternized chitosan scaffold (dGQ) integrated with poly(ionic liquid)s (PILs) (dGQP) for skin tissue engineering. The scaffold was fabricated using extrusion-based 3D bioprinting and dynamic hydrogen-bonding techniques. The results indicated that the rapid release of the PILs from the dGQP scaffold within 2 h effectively eliminated both Gram-negative (E. coli) and Gram-positive (S. aureus) bacteria, achieving nearly 100% antibacterial activity and maintaining a sterile environment for up to 7 days, outperforming the dGQ scaffold. Hemostasis and hemolysis tests demonstrated that the dGQP scaffold exhibited excellent hemostatic properties and hemocompatibility. Overall, this advanced dGQP scaffold shows strong potential for applications in skin tissue engineering.

Quaternized CS derivatives have demonstrated considerable potential in a wide range of biomedical applications, including wound healing, tissue engineering, and delivery systems for drugs and vaccines.

### 4.2. Acylated CS

*N*-acylated CS derivatives exhibit enhanced biocompatibility, anticoagulability, and blood compatibility, often without triggering inflammatory responses. These properties make them suitable carriers or sustained-release agents for pharmaceutical applications [25,28,128]. The introduction of hydrophobic groups imparts new properties, enabling the formation of various structures, like gels, vesicles, and fibers. *N*-acylated CS with high solubility can serve as a carrier for hydrophobic drugs, while those with high crystallinity enhance fiber toughness and thermal stability, finding applications in materials like PVC films [6]. Additionally, *N*-acylated CS can be used as a template for bone tissue materials, such as hydroxyapatite, and as carriers or sustained-release agents in drug delivery and biological scaffolds [129,130,131].

The *O*-acylation of chitosan disrupts its hydrogen-bonding structure, leading to increased fat solubility and hydrophobicity [6,132]. Unlike *N*-acylated chitosan, which exhibits improved water solubility, *O*-acylated chitosan is soluble in non-polar solvents such as pyridine and chloroform [6]. These hydrophobic properties make *O*-acylated chitosan suitable for applications in films, fibers, and polymeric materials where enhancing the water resistance and stability is essential [6,133].

Acylated CS derivatives are utilized with hydrophobic drugs to modify their hydrophobicity and control their release. By providing controlled drug release and enhanced permeability, these derivatives help to maintain a sustained therapeutic effect, which is crucial for chronic treatments where consistent drug levels are necessary.

#### 4.2.1. Succinyl CS

*N*-succinyl chitosan (NSucCS), an amphiprotic derivative of chitosan with enhanced biocompatibility, aqueous solubility, and transfection efficiency, has demonstrated the potential for improved bioavailability [29]. Its pH sensitivity and prolonged circulation time in the human body contribute to its suitability as a drug delivery system. NSucCS exhibits additional advantageous properties, including mucoadhesion due to its negative charge, anticoagulant and antiaggregant effects without compromising the hemodynamics, and low cytotoxicity. Moreover, its ability to promote cell adhesion, proliferation, and differentiation, coupled with minimal foreign body reactions, highlights its potential for tissue engineering and bone regeneration [29].

**Figure 28:**
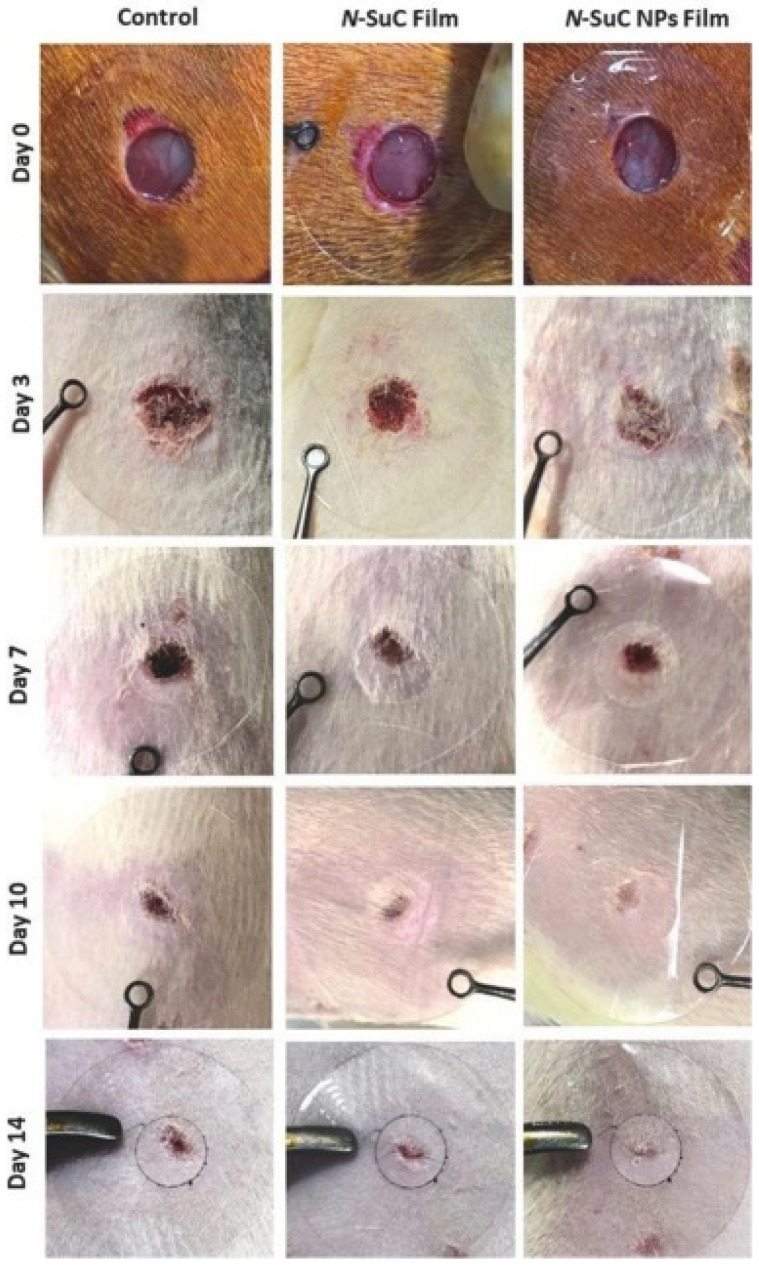
In vivo photographs reveal that NSuC and NSuC NP films significantly accelerated wound closure compared to the control group. The treated wounds exhibited enhanced re-epithelialization, reduced inflammation, and increased granulation tissue formation, as evidenced by the visual representation of the healing process over 14 days [34].

Recent research has focused on developing novel *N*-succinyl chitosan (NSucCS) nanoparticle (NSuC NP)-based films for wound healing. Thao et al. [34] successfully synthesized NSuC NPs through an ionic gelation method and then fabricated NSuC NP-based films using a solution casting technique. These films exhibited promising antimicrobial properties against both Gram-negative (*Escherichia coli*) and Gram-positive (*Staphylococcus aureus*) bacteria. To assess the biocompatibility and wound-healing potential of the NSuC NP films, in vitro studies were conducted using human dermal fibroblasts. The results indicated good cell viability and proliferation, suggesting the biocompatibility of the material. Furthermore, in vivo wound-healing experiments on Wistar rats demonstrated accelerated wound closure, characterized by increased blood vessel formation and tissue granulation compared to the control group (Figure 28). These findings highlight the potential of NSuC NP-based films as a promising candidate for wound dressing and tissue regeneration applications.

*N*-succinyl CS (NSC) has shown promise in various biomedical applications due to its improved solubility and biocompatibility compared to native CS [29]. Lee et al. [134] utilized NSC to create ionically crosslinked hydrogels with glucose-6-phosphate (G6P), resulting in materials with rapid degradation properties, suitable for bone graft delivery. In another study, Wang et al. [83] incorporated NSC into nanohybrids with mangiferin for diabetes treatment, demonstrating improved drug delivery and therapeutic efficacy.

NSC’s inherently weak mechanical strength, a consequence of its natural polymer properties, can limit its application in drug delivery systems. Combining NSC with synthetic polymers offers a potential solution to this challenge. Bashir et al. [135] demonstrated this approach by crosslinking NSC with poly(methacrylic acid), resulting in improved mechanical strength, controlled swelling, and a stable gel network. The combination leveraged the degradability of NSC, the mechanical strength of poly(methacrylic acid), pH sensitivity, and mucoadhesive properties, making this crosslinked hydrogel suitable for site-specific and controlled drug delivery.

Succinylated derivatives have been shown to facilitate the sustained release of drugs, reducing the initial burst effect and maintaining consistent drug levels over time. This results in increased bioavailability and a more predictable therapeutic profile [136,137]. Shripetthong et al. [136] developed nanomicelles using *N*-benzyl-*N*,*O*-succinyl chitosan (NBSCh) encapsulated with a curcumin analog, 2,6-bis((3-methoxy-4-hydroxyphenyl)methylene) cyclohexanone, also known as cyqualone (CL), for the targeting of colon cancer. The CL-NBSCh micelles exhibited significantly higher selective cytotoxicity towards human colon cancer (HT-29) cells, while showing reduced toxicity to mouse connective tissue fibroblasts (L929) compared to free CL. Additionally, the CL-NBSCh micelles were more effective than free CL in halting cell growth at the G2/M phase and induced earlier apoptosis in HT-29 cells. These findings highlight the promising potential of CL-loaded NBSCh micelles as an oral treatment for colon cancer.

Succinylated CS derivatives are designed for targeted drug delivery. The succinyl groups facilitate the attachment of targeting ligands, enabling the precise delivery of drugs to specific tissue types or cells. This targeted approach minimizes the side effects and maximizes the therapeutic efficacy. Jain et. al. [138] studied lactose–acyclovir–*N*-succinyl chitosan nanoparticles (Lac-*N*-Suc-CSNP), using lactose as an asialoglycoprotein receptor (ASGPR) ligand for hepatic parenchymatic cell targeting. The acyclovir concentration in the Lac-*N*-Suc-CSNP was 19.9 ± 1.62 μg/g after 24 h administration, revealing remarkable targeting potential toward the hepatocytes and maintaining a high level during the experiment. These results suggest that Lac-*N*-Suc-CSNP are a potential vector for hepatocyte targeting.

Succinylated CS derivatives possess promising application potential in drug delivery, wound dressings, and tissue engineering.

#### 4.2.2. Benzoyl CS

Benzoyl CS biopolymers have shown potential in various applications, including drug delivery, cosmetics, wound healing, and chromatographic separation technologies.

Benzoylated CS derivatives enhance the hydrophobic interactions between the drug and the polymer, which is beneficial for hydrophobic drugs. This improvement in encapsulation efficiency and sustained release translates to better bioavailability and a longer duration of action for the encapsulated drug [112].

The main enhanced property is the antimicrobial activity of these derivatives. Mohamed et al. [39] employed benzoylated CS as a hydrogel component to eradicate *Helicobacter pylori*. By crosslinking CS with varying amounts of 4,4′-(5,5′-carbonylbis(1,3-dioxoisoindoline-5,2-diyl))dibenzoyl isothiocyanate, they created four hydrogels (BBTU-Cs-1, BBTU-Cs-2, BBTU-Cs-3, and BBTU-Cs-4) with increasing crosslinking densities. These hydrogels demonstrated the selective inhibition of COX-2, with BBTU-Cs-4 exhibiting potent activity comparable to celecoxib.

#### 4.2.3. *N*-Phthaloylated CS

Phthaloyl CS has gained interest in oral drug delivery due to its pH-dependent solubility, being low in acidic environments and high in basic conditions. Aiedeh and Taha demonstrated that CS phthalate protects drug molecules in at an acidic pH 2.0, releasing them primarily at pH 7.4 [41]. *N*-phthaloylation effectively protects CS’s amine groups, but it also increases the crystallinity and solubility in aprotic polar solvents like DMSO, DMAc, DMF, and pyridine [139].

Phthaloyl CS has found application in drug delivery through the creation of nano- or microspheres [140,141]. Ubaidulla et al. [141] synthesized CS phthalate microspheres using the emulsion phase separation technique and loaded them with insulin via passive absorption. These microspheres demonstrated the protection of insulin from gastric enzyme degradation, enhancing its oral stability. The insulin release profile exhibited pH-dependent behavior, with rapid release in phosphate-buffered saline (pH 7.4) and minimal release in acidic conditions (pH 2.0). This pH sensitivity is attributed to the ionization state of the carboxylic groups within the microspheres. This study suggests the potential of CS phthalate microspheres as a carrier for oral insulin delivery.

Karuna et al. [142] developed a colon-specific drug delivery system for diclofenac sodium using CS phthalate-based multiparticulate spheres prepared through extrusion and spheronization. This formulation significantly increased diclofenac’s peak plasma concentration and oral bioavailability compared to pure diclofenac. Additionally, the system demonstrated enhanced anti-inflammatory activity and a reduced ulcer index.

*N*-phthaloylated CS derivatives are used with drugs that require improved stability and solubility. These derivatives enhance the stability and bioavailability of drugs, making them suitable for various pharmaceutical applications where maintaining drug integrity is crucial.

#### 4.2.4. Thiolated CS

The thiolation of chitosan leads to new properties gained by the covalent attachment of thiol groups. Thiolated chitosans exhibit great adhesion to biological surfaces such as mucins or keratins. The crosslinking of thiolated chitosans due to disulfide formation improves their in situ gelling properties and mechanical stability. They show adjustable swelling behavior when using different ligands and degrees of S-preactivation. They are capable of the controlled release of covalently bound drugs or prolonged drug release from crosslinked polymers. They offer a permeation enhancement due to the opened tight junctions caused by the interaction with cysteine-bearing membrane receptors and enzymes. They cause the inhibition of efflux pumps and enzymes due to the formation of disulfide bonds. They are capable of the complexation of metal ions via their sulfhydryl groups. They can inactivate reactive oxygen species through disulfide formation [45].

The enhanced mucoadhesive properties of thiolated CS make it a preferred choice for the development of novel drug delivery systems, as well as mucosal drug and gene delivery [44,143]. The introduction of thiol groups into CS, such as through the use of 2-iminothiolane, has led to significant improvements in mucoadhesion. Microparticles composed of chitosan–4-thio-butyl-amidine conjugates (CS–TBA) demonstrated enhanced potential for nasal insulin delivery compared to unmodified CS [144]. While CS–thioethylamidine did not exhibit a significant difference in its swelling properties compared to unmodified CS, it demonstrated improved mucoadhesion. The sustained release of fluorescein isothiocyanate-dextran over 3 h using CS–thioethylamidine was attributed to the presence of disulfide bonds within the CS structure, which hindered the diffusion of macromolecules [46].

CS–glutathione conjugates have emerged as promising materials due to glutathione’s permeation-enhancing properties, antioxidant activity, and favorable toxicological profile [46,144,145,146]. The thiol group within glutathione contributes to its strong reducing and electron-donating capabilities. This combination has been successfully applied in drug delivery systems, as demonstrated by Jin et al. [147], who utilized CS–glutathione-coated nanoparticles to enhance the oral delivery and efficacy of thymopentin in immunosuppressed rats.

**Figure 29:**
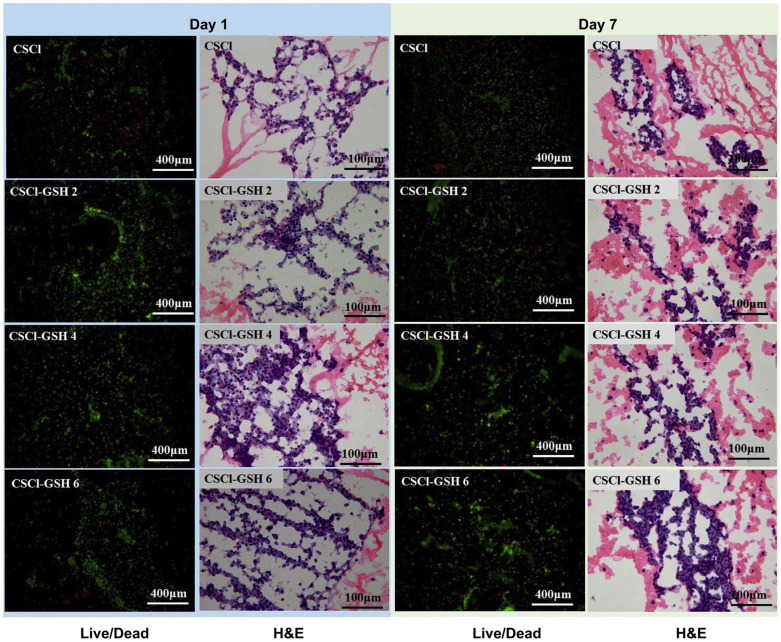
Live/dead and H&E staining images demonstrate increased cell viability and maintained cell morphology in CSCl–GSH hydrogels compared to CSCl hydrogels at days 1 and 7 [148].

A CS–glutathione hydrogel (CSCl–GSH) has demonstrated enhanced efficacy in reducing oxidative stress in neonatal rat cardiomyocytes compared to an unmodified CS hydrogel. This improved performance is attributed to the enhanced cellular adhesion potential of CSCl–GSH, facilitated by the presence of biocompatible glutathione, which promotes cell survival. Live/dead staining and hematoxylin and eosin (H&E) staining analyses revealed that the CSCl–GSH hydrogel supported cardiomyocyte adhesion and survival, with fewer dead cells observed compared to the unmodified CS hydrogel (Figure 29) [148].

Photodynamic and photothermal therapies are methods that utilize electromagnetic energy as a trigger. In photodynamic therapy, a photosensitizer is used to produce reactive oxygen species (ROS) upon activation. In contrast, photothermal therapy involves a metal that converts electromagnetic energy into heat through surface plasmon resonance. Both approaches employ ROS or heat to selectively destroy cells. Manivasagan et al. [149], in their study, developed new thiol chitosan-wrapped gold nanoshells (TC-AuNSs) as an antibacterial agent for the near-infrared (NIR) laser-triggered photothermal destruction of antibiotic-resistant pathogens, such as Gram-positive bacteria (*Staphylococcus aureus*) and Gram-negative bacteria (*Pseudomonas aeruginosa* and *Escherichia coli*). The TC-AuNSs were capable of destroying *S. aureus*, *P. aeruginosa*, and *E.coli* within 5 min of NIR laser irradiation, and no bacterial growth was detected on the tryptic soy agar (TSA) plate after 48 h of laser irradiation. This indicates that TC-AuNSs are highly efficient in killing bacteria quickly and preventing bacterial regrowth.

The introduction of thiol groups increases the viscosity, which is a desirable characteristic for tissue engineering and wound healing [44].

Le-Vinh et al. [150] designed S-protected thiolated CS derivatives with enhanced cell adhesion properties for tissue engineering applications. By covalently attaching 3-((2-acetamido-3-methoxy-3-oxopropyl)dithio)propanoic acid to CS, they created three derivatives (Ch-SS-1, Ch-SS-2, and Ch-SS-3) with varying degrees of modification. For comparison, thiolated CS derivatives (Ch-SH-1, Ch-SH-2, and Ch-SH-3) were also prepared. The study found that Ch-SS-1 exhibited superior cell adhesion compared to Ch-SS-2 and Ch-SS-3, attributed to its more lipophilic surface. While all thiolated CS derivatives supported cell adhesion, growth, and proliferation, the SSg scaffolds with interconnected microporous structures demonstrated even better cell migration, adhesion, and proliferation. These findings highlight the potential of Ch-SS derivatives for the construction of tunable cryogel scaffolds suitable for 3D cell culture and tissue engineering.

Thiolated CS derivatives are known for their enhanced mucoadhesion properties, which make them potentially beneficial in biomedical applications such as new drug delivery systems, wound healing, and tissue engineering.

#### 4.2.5. PEGylated CS

Polyethylene glycol (PEG) is a versatile polymer with widespread application due to its excellent properties as a solvent, plasticizer, surfactant, base, and lubricant [151]. In the pharmaceutical field, PEG offers several advantages, including increased drug circulation times, reduced immunogenicity, and enhanced drug solubility. By increasing the molecular weight of pharmaceutical products, PEG can prolong their circulation times and protect them from degradation. Additionally, the PEGylation of nanoparticles reduces protein adsorption, thereby extending their circulation times and improving their biodistribution [57,152].

PEG-grafted (g) CS has shown potential as a carrier to enhance the solubility and bioavailability of astaxanthin (ASTA), a carotenoid with various health benefits. While ASTA-PEG-g-CS nanoparticles exhibit improved bioavailability compared to free astaxanthin, the high cost and lengthy synthesis time of grafted materials present challenges. Zhu et al. [153] aimed to develop a more efficient method of producing and evaluating ASTA-PEG-g-CS nanoparticles. By addressing the solubility and bioavailability limitations of astaxanthin through nanoencapsulation, this approach holds promise in expanding the clinical and food industry applications of this valuable compound.

Sultan et al. [154] developed a novel anticancer drug delivery system through cisplatin-loaded PEGylated chitosan nanoparticles. The drug release kinetics of cisplatin showed zero-order kinetics with 48% of drug release in a linear manner. Studies on the MCF-7 ATCC human breast cancer cell line in vitro revealed that the IC50 value was 82.08 µg /mL. The injectable nanoparticles had good physicochemical and cytotoxic properties.

**Figure 30:**
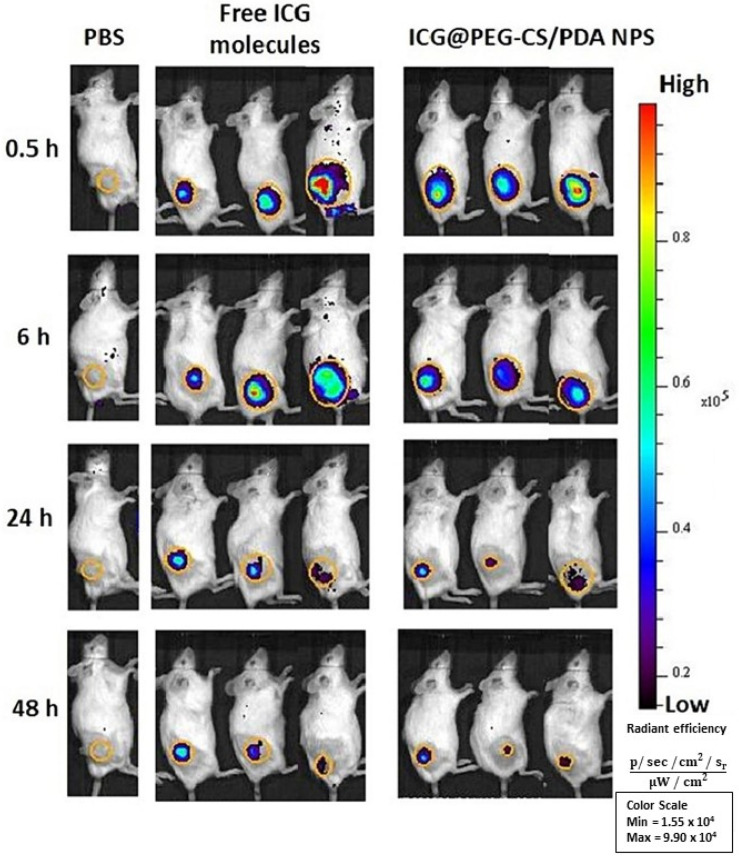
In vivo NIR fluorescence images of CT26 tumor-bearing mice receiving an intratumoral injection of free ICG molecules or ICG@PEG-CS/PDA NPS by IVIS. The tumor sites are labeled with yellow circles [155].

PEGylated CS can be used to form nanoparticles designed for combined photothermal and photodynamic therapy in cancer treatment. Chen et al. [155] developed a multifunctional nanophotosensitizer (NPS) system by integrating indocyanine green (ICG) into PEGylated chitosan (PEG-CS)-coated polydopamine (PDA) nanoparticles through multiple π–π stacking, hydrophobic, and electrostatic interactions (ICG@PEG-CS/PDA NPS). Once internalized by CT26 cells, these nanoparticles, when exposed to near-infrared laser light, generated significant amounts of singlet oxygen. This process was enhanced by the thermo-induced depletion of intracellular GSH, leading to mitochondrial damage and lipid peroxide formation, which in turn caused ferroptosis and apoptosis. Notably, the combination of photothermal therapy (PTT) and photodynamic therapy (PDT) provided by the NPS effectively inhibited CT26 tumor growth in vivo through intense hyperthermia activated by light and the disruption of redox homeostasis. Figure 30 shows the tumor sites injected with free ICG molecules and ICG@PEG-CS/PDA NPS, respectively. Through the intratumoral injection of free ICG molecules and ICG@PEG-CS/PDA NPS, an equal amount of ICG accumulation within the tumor was realized. On the other hand, for the free ICG and ICG@PEG-CS/PDA NPS groups, the tumor sites showed stronger ex vivo ICG fluorescence intensities than other organs, corresponding to the typical biodistribution of ICG molecules injected intratumorally.

PEGylated CS derivatives are employed with drugs that benefit from increased hydrophilicity and reduced immunogenicity. These modifications improve the drug’s solubility and stability, resulting in prolonged circulation times and enhanced bioavailability, enabling them to be applied in new drug delivery systems.

#### 4.2.6. CS Derivatives with Cholic and Deoxycholic Acid

Bile acids, specifically deoxycholic acid (DOCA), have attracted attention for their potential in polymer modification. These amphiphilic molecules exhibit enterohepatic organotropism and possess suitable hydrophobicity for polymer conjugation [156,157]. DOCA’s carboxylic and hydroxyl groups offer convenient conjugation sites, potentially aligning with the bile acid-binding site of the apical sodium–bile acid transporter [158]. Moreover, DOCA-modified polymers demonstrate reduced cytotoxicity and enhanced penetration compared to other amphiphilic bile acid-conjugated polymers [159].

CS derivatives with deoxycholic and cholic acid derivatives are used with hydrophobic drugs to enhance their solubilization and transport. By forming micelles or other self-assembled structures, CS derivatives with cholic and deoxycholic acid improve the absorption and bioavailability of poorly soluble drugs in oral drug delivery [160,161,162]. Cholic acid has been utilized as a drug delivery shuttle targeting the liver [157]. Park et al. [160] developed cholic acid-conjugated CS nanoparticles for paclitaxel delivery, demonstrating significantly enhanced anticancer activity against B16F10 cells compared to a Cremophor EL–ethanol formulation. Similarly, Ding et al. [60] prepared cholic acid and galactose-conjugated chitosan derivatives as potential anti-liver cancer drug carriers to enhance the aqueous solubility of sorafenib. Due to the inclusion complex with the chitosan conjugate, the solubility of sorafenib in water was increased from 1.7 mu g/mL to 1900 mu g/mL, which is equal to an 1117-fold increase in its solubility.

Rhein (RH), a potential treatment for kidney diseases, suffers from low aqueous solubility and oral bioavailability. To address these limitations, deoxycholic acid-conjugated nanoparticles (DNPs) were developed to enhance RH’s intestinal absorption by targeting the apical sodium-dependent bile acid transporter [159].

To enhance the oral bioavailability of azathioprine, Arshad et al. [163] designed a cholic acid-grafted thiolated chitosan (CA-CS-TGA) polymeric biomaterial to achieve improved permeation via attaching thiol groups and cholic acid moieties, and the prepared CA-CS-TGA graft was used to coat azathioprine-loaded nanoliposomes (NLs). Ex vivo permeation enhancement and relative oral bioavailability studies indicated the 2.84-fold enhanced permeation and six-fold enhanced oral bioavailability of the CA-CS-TGA-NLs compared to an azathioprine suspension.

CS–cholic acid derivatives are suitable for the solubilization and transport of drugs and even liver-targeted drug delivery. The cholic acid moiety improves the interaction with bile acids and enhances the absorption of drugs in the gastrointestinal tract, making them effective for oral drug delivery systems.

#### 4.2.7. CS–EDTA-Crosslinked Polymer

The introduction of EDTA to CS transforms the polymer from cationic to anionic, resulting in enhanced mucoadhesive properties due to hydrogen bond formation with mucus. This modified CS, known as chitosan–EDTA, offers potential as a drug delivery carrier due to its controlled release capabilities [62]. Furthermore, the anionic nature of chitosan–EDTA can protect DNA from nuclease degradation. EDTA, as a chelator, binds divalent cations that are essential for enzyme activity, thereby inhibiting their function and safeguarding DNA in extracellular fluids [164].

Loretz et al. [63] chemically attached EDTA to chitosan (CS) to combine the polymer’s bioadhesive properties with EDTA’s well-known ability to bind metal ions, which are crucial for protease enzymatic activity. The inhibitory effect of this CS–EDTA conjugate was assessed using leucine enkephalin (Leu enkephalin) as a model drug. HPLC analysis was employed to measure the extent of Leu enkephalin degradation by aminopeptidase N (EC 3.4.11.2) and porcine mucosa in the presence of the polymer conjugate. The study also highlighted the potential of CS–EDTA conjugates of different molecular weights as carrier matrices for nanoparticulate gene delivery systems. The covalent attachment of EDTA to multiple CS chains resulted in a crosslinked polymer, expected to produce stabilized particles. Among the tested variants, CS–EDTA derived from low-viscosity CS with 68% of its amino groups modified by EDTA showed the highest metal-complexing efficiency. Additionally, the cytotoxicity of the CS–EDTA particles was found to be below 1% over 4 h. These new nanoplexes demonstrated a 35% improvement in the in vitro transfection efficiency compared to unmodified CS nanoparticles, suggesting that the CS–EDTA conjugate holds promise as a polymer for gene transfer applications.

El-Sharif and Hussain [62] investigated the antimicrobial properties of CS derivatives, EDTA, and the novel CS–EDTA combination against various bacteria and *Candida albicans*. The combination demonstrated synergistic antimicrobial activity against *Staphylococcus aureus* and additive effects against other microorganisms. Notably, short-term exposure to chitosan–EDTA resulted in complete microbial eradication. These findings suggest the potential of CS–EDTA as an enhanced antibacterial and antifungal agent in pharmaceutical formulations.

CS–EDTA has the potential to be applied to dentin resin for direct bonding restoration. Zhou et al. [165] found that the application of CS–EDTA to dentin could extrafibrillarly demineralize collagen fibers. The microtensile strength test found that the bonding strength and durability after the application of CS–EDTA were equivalent to those of SE-Bond and better than the phosphoric acid wet bonding commonly used clinically (*p* < 0.05). The cytotoxicity of chitosan–EDTA was lower than that of phosphoric acid and SE-Bond in the CCK-8 assay and lower than that of phosphoric acid in the microfluidics experiment. EDTA, a potent chelating agent with FDA approval for heavy metal poisoning treatment since the 1950s, offers several advantages in drug delivery. Its ability to remove ions has been linked to the enhanced permeation of antiviral drugs across cellular barriers [63]. Additionally, EDTA can inhibit the pre-systemic metabolism of peptide-based drugs by chelating essential ions for brush border membrane-bound enzyme activity [166].

CS–EDTA-crosslinked polymers are used to enhance drugs’ stability and control their release. This crosslinking provides a more stable and controlled release system, improving the pharmacokinetic profile of the encapsulated drug.

#### 4.2.8. CS Derivatives with Sugar Parts: CS–Cyclodextrin Complex

Cyclodextrins (CDs) are cyclic oligosaccharides composed of six to nine glucose units linked by (1→4) glycosidic bonds. Their distinctive cone-shaped structure, featuring a hydrophobic cavity enclosed by a hydrophilic exterior, allows for the formation of inclusion complexes with various hydrophobic molecules. This property enables CDs to enhance the solubility and dissolution of poorly water-soluble drugs, particularly those containing aromatic or substituted aromatic groups [167,168].

Venter et al. [67] investigated the mucoadhesive properties of cyclodextrin–CS derivatives using a tensile separation test. While derivatization reduced the mucoadhesive properties of CS by 13.5% compared to pectin, the derivative demonstrated 12% greater strength. In contrast, Chaleawlert-Umpon et al. [169] synthesized citrated cyclodextrin-g-CS, using citric acid as a crosslinking agent. This approach enhanced mucoadhesive properties through electrostatic interactions between CS’s amino groups and mucin, as well as hydrogen bonding between the citrate spacers and mucus glycoproteins. The presence of citric acid also contributed to the biocompatibility and functionality of the resulting CS derivative, expanding its potential for drug delivery and other therapeutic applications.

Glycidyl trimethylammonium chloride has been used to quaternize CS, leading to enhanced mucoadhesion when combined with citrate modification [170].

Daimon et al. [171] investigated the interaction between CS-g-CDs and insulin, revealing the strong binding of insulin to β-CD residues through host–guest inclusion complex formation involving insulin’s amino acid side chains. Electrostatic interactions between chitosan-g-CDs and insulin further strengthened the binding across a wide pH range.

CS-g-CDs have emerged as promising carriers for the delivery of peptides and proteins, demonstrating their potential as key components in the development of advanced drug delivery systems, such as nanovehicles and stimuli-sensitive carriers [172,173].

Norouzi and Abdouss [174] developed nanofibers based on β-CD-g-CS macromolecules containing drugs using the blend electrospinning technique. Indomethacin was encapsulated within the β-CD-g-CS matrix as blend nanofibers through electrospinning in the presence of polyvinyl alcohol (PVA). The drug release profiles from the blend electrospun nanofibers, CS, and β-CD-g-CS showed initial burst release followed by sustained release, almost in a linear pattern. The growth rate of L929 cells on both CS and β-CD-g-CS nanofibers was not significantly hindered and they even promoted cell proliferation. These results suggest that β-CD-g-CS nanofibers could be an effective smart drug delivery system for the sustained release of indomethacin, making them promising candidates for anti-inflammatory treatments in wound healing and tissue engineering, particularly in orthopedic applications.

Hao et al. [175] synthesized β-cyclodextrin-grafted chitosan (CD-g-CS) with different degrees of substitution. Then, they prepared novel thermosensitive β-cyclodextrin-grafted chitosan/glycerophosphate (CD-g-CS/GP) hydrogels. In vitro release studies revealed that the hydrogels showed sustained release and enhanced the solubility and bioavailability of curcumin (CUR). The evaluation of the antibacterial activity indicated that the curcumin-loaded CD-g-CS/GP hydrogels were effective antibacterial materials for *Staphylococcus aureus* and *Escherichia coli*. This CD-g-CS/GP hydrogel might be potentially used in a biodegradable smart drug sustained release system for the controlled release of hydrophobic drugs.

CS–CD derivatives can be also suitable for tissue engineering applications. Lee et al. [176] fabricated CS-based electrospun nanofibers (Ens) by using an electrospinning (ELSP) system, followed by surface modification using succinyl-beta-cyclodextrin (β-CD) under mild conditions. The β-CD-modified CTS (βCTS) ENs had slightly increased hydrophobicity compared to pristine CTS ENs and decreased the residual amine content on the surface. The drug release was sustained for a hydrophobic drug (e.g., dexamethasone) loaded on the β-CD ENs. During in vitro biocompatibility assessments, the grafting of β-CD was shown not to reduce the cell viability compared to pristine CTS ENs. Additionally, cells proliferated well on the β-CD ENs, and this was confirmed by F-actin fluorescence staining.

CS derivatives with cyclic saccharide structures offer enhanced mucoadhesion, stability, and controlled release properties. These modifications improve the bioavailability and therapeutic efficacy by providing the more predictable and sustained release of drugs. CS derivatives with cyclodextrin form inclusion complexes with drugs, enhancing their solubility and stability. This leads to improved drug release profiles and increased bioavailability, making these derivatives suitable for various pharmaceutical applications.

#### 4.2.9. CS–Crown Ether Derivatives

Crown ethers have good complex selectivity for many metal ions. However, they are not easily recycled after being used; therefore, their applications are limited. When crown ethers were crosslinked with chitosan chains, these new crown-ether-crosslinked chitosan derivatives contained double structures and had stronger complexing properties with better selectivity for metal ions than the corresponding crown ethers and crosslinked chitosan. They possessed space net structures with crown ethers embedded within them, and each mesh had a certain space volume. Crown-ether-bound chitosan not only had good adsorption capacities for noble metal ions such as Pd^2+^, Au^3+^, and Ag^+^ but also had high selectivity for the adsorption of Pd^2+^ in the presence of Cu^2+^ and Hg^2+^ [27,112].

The complex of this derivative and silver ions has potential in the medical application of bacteriostasis. Yi et al. [177] synthesized crown-ether-crosslinked chitosan (CCTS-1) by reacting 4,4′-diformyldibenzo-18-c-6 crown ether with crosslinked chitosan (CS). They then prepared a new type of di-secondary amine crown-ether-crosslinked chitosan (CCTS-2) by reacting CCTS-1 with sodium borohydride. CCTS-2 demonstrated an impressive adsorption rate of 96% for Ag^+^ over 1 h at pH 6.0, with an initial concentration of 0.5 mmol/L. The diameters of the bacteriostasis zones for the CCTS-2–Ag+ complex, containing 0.00355 mmol Ag^+^, were 11 mm against *Staphylococcus aureus*, 10 mm against *Escherichia coli*, and 7.5 mm against *Pseudomonas aeruginosa*. In comparison, the CCTS–Ag^+^ complex under similar conditions showed diameters of 11 mm, 10 mm, and 6.0 mm, respectively. This provides valuable insights for the design of crosslinked chitosan-based adsorbents for the pre-concentration of Ag^+^ in medical bacteriostasis applications.

### 4.3. Other Covalently Bound CS Derivatives

#### 4.3.1. Sulfonated CS

Due to their polyampholytic properties, sulfonated CS derivatives are extensively utilized in biomedical applications. The sulfonation process increases the water solubility of CS derivatives by introducing sulfonic acid groups. For instance, Tsai et al. [78] demonstrated the enhanced water solubility of an *N*-sulfopropyl CS derivative.

Sulfonated CS also exhibits anticoagulant properties, although they are less potent than those of heparin and can delay clot formation. This effect is influenced by both the degree of substitution and the concentration of sulfonated CS. For instance, an *N*-sulfofurfuryl CS derivative demonstrated anticoagulant activity and maintained water solubility across a broad pH range (from pH 2 to pH 12), underscoring its amphoteric nature. Additionally, it showed non-thrombogenic properties by affecting platelet adhesion and activation, making it suitable for certain blood-contacting applications [76].

Sulfonated CS derivatives, when used with drugs, enhance their solubility and bioavailability by improving the drugs’ interactions with the polymer. This results in more efficient drug delivery and a better therapeutic effect. Murali et al. [178] prepared functionalized chitosan nanoparticles (AmpB-SCSNPs) for the proficient macrophage delivery of amphotericin B (AmpB) to manage *Candida glabrata* fungemia. The AmpB-SCNPs markedly improved the reduction of *C. glabrata* compared to both bare AmpB and AmpB-CNPs (55.2 and 42.7 vs. 11.12 cfu/mL). This suggests that AmpB-SCNPs could serve as a promising carrier for the targeted delivery of AmpB to macrophages, enhancing the effectiveness of treatment for *Candida glabrata* fungemia.

Sulfated CS has shown enhanced antiviral properties against HIV-1, outperforming other CS derivatives due to its structural similarity to heparin. Specifically, 3,6-*O*-sulfated chitosan demonstrated the effective inhibition of a broad range of HPV pseudoviruses (HPV PV) [179]. Moreover, sulfated CS retains the antimicrobial activity of pure CS while offering improved solubility. Sun et al. [80] reported that sulfonated CS exhibited superior antibacterial activity against *Escherichia coli* and *Staphylococcus aureus*, with minimum inhibitory concentrations (MIC) of 0.13 mg/mL and 2.00 mg/mL, respectively, compared to water-soluble chitosan (WCS), which had MIC values of 0.50 mg/mL and 4.00 mg/mL.

Sulfated CS is being explored for various drug delivery systems, including nanoparticles, prodrugs, membranes, and polymeric micelles [180]. Wang et al. [181] prepared four types of doxorubicin (DOX)-loaded polymeric micelles based on hydrophobically modified sulfated chitosan (SCTS). The hydrophobic group was formed by glycyrrhetinic acid (GA), cholic acid, stearic acid (SA), or lauric aldehyde. GA–SCTS micelles had the best capability to solubilize DOX, and, in addition, they could target HepG_2_ cells (the IC50 for DOX-loaded GA–SCTS micelles was 54.7 ng/mL, which was much lower than those of the other micelles). They were also stable in salt and protein solutions, in cell culture media, and during long-term storage (6 months). Therefore, these micelles may offer a promising DOX-encapsulated formulation, particularly GA–SCTS, as a potential vehicle for liver-targeted delivery.

**Figure 31:**
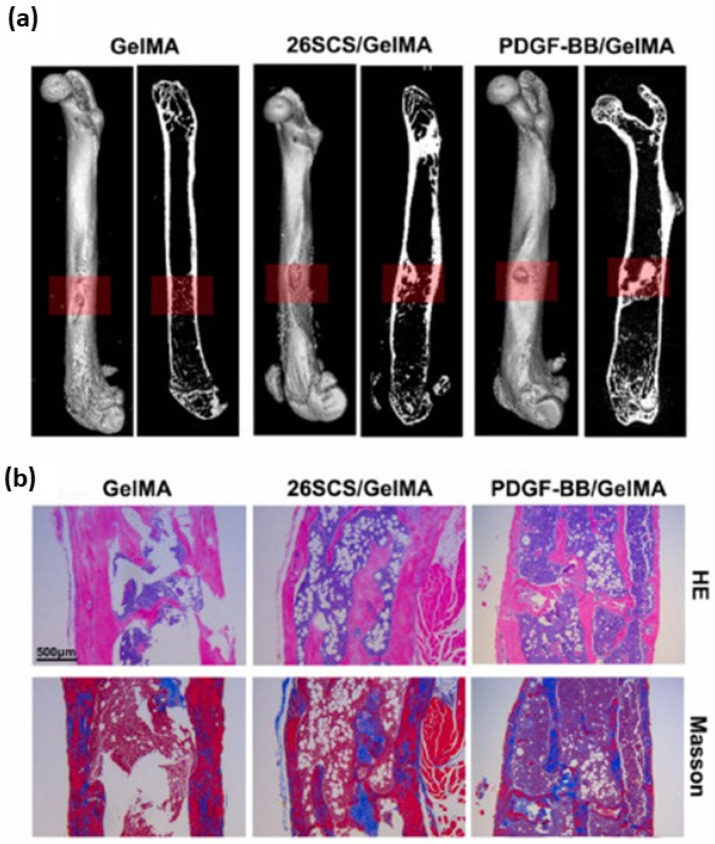
(**a**) Representative micro-CT images of femurs from OVX (ovariectomy) mice implanted with different scaffolds, gelatin methacryolyl scaffold (GelMA), 26SCS/GelMA scaffold, and PDGF-BB/GelMA scaffold, at 8 weeks post-surgery. Additionally, panel (**b**) includes representative images of H&E and Masson staining for the femurs from the GelMA, 26SCS/GelMA, and PDGF-BB/GelMA groups [182].

Sulfated CS is an excellent biomaterial for tissue engineering due to its hemocompatibility, anticoagulant properties, and additional benefits like neovascularization and immunostimulatory effects. This makes it particularly effective for bone reconstruction, creating vascular networks, and stimulating the immune response. Sulfated CS has also been shown to enhance the biological activity of bone morphogenetic proteins, which are crucial for bone formation and tissue repair. Recent research by Ji et al. [182] demonstrated that 2-N, 6-O sulfated CS (26SCS) enriched periosteal stem cells (PSCs) and significantly promoted osteogenesis in bone defect areas. This was particularly evident in an osteoporotic mouse model with reduced platelet-derived growth factor-BB (PDGF-BB) levels and compromised regenerative potential. At the eight-week mark, the micro-CT analysis of femurs from three groups revealed more bone formation in the 26SCS/GelMA and PDGF-BB/GelMA groups, as shown in Figure 31.

Sulfated CS derivatives are known for their enhanced water solubility and biological activity. The addition of sulfonic acid groups imparts anticoagulant, anti-inflammatory, and antiviral properties to chitosan. These derivatives are used in various biomedical applications, including drug delivery systems, where they can improve the solubility and bioavailability of drugs, and in tissue engineering, where they can enhance cell proliferation and differentiation.

#### 4.3.2. Phosphorylated CS

Phosphorylated CS (PCS) and its derivatives are notable for their high water solubility, metal chelation ability, bactericidal activity, biocompatibility, bioabsorbability, and osteoinductive properties. The phosphate groups enhance the osteoconductivity and cell differentiation, making them suitable for bone regeneration and other tissue engineering applications [86,183,184].

Anushree et al. [185] synthesized a PCS derivative and evaluated it for its diabetic wound-healing capabilities. The wound-healing potential of PCS was evaluated using a diabetic excisional wound rat model. PCS showed good water solubility and in vitro antioxidant capacity. On the 14th day post-wound creation, wound contraction was significantly greater in PCS-treated wounds (91.11%) compared to untreated wounds (67.26%). The histopathological analysis of PCS-treated wounds indicated an improved tissue morphology, with an increased number of fibroblasts, a thicker epithelial layer, and enhanced collagen deposits and angiogenesis compared to untreated wounds. There was an overall increase of 57% in the hydroxylamine content and 25% in the hexosamine content in treated wounds relative to untreated ones. These findings suggest that PC is an effective agent for the promotion of healing in diabetic wounds.

Due to its cation-exchange properties, phosphorylated CS (PCS) holds promise for orthopedic applications. The phosphate groups in PCS can chelate calcium ions, promoting the formation of an apatite-like layer that enhances the osteoconduction of polymer-based implants. Furthermore, the combination of negatively charged phosphate groups with the positively charged amine groups in CS endows PCS with an amphoteric nature. This dual charge property could facilitate the immobilization of signaling molecules, such as growth factors, offering potential advantages for combinatorial therapeutic strategies [186].

In a recent study, Wenxiu et al. [187] developed a composite scaffold combining bioactive glass with phosphorylated chitosan (PCS) for bone repair applications. The scaffold demonstrated excellent biocompatibility, as MC3T3-E1 cells adhered to, spread on, and proliferated within it. Additionally, the scaffold exhibited osteoinductive properties, evidenced by increased alkaline phosphatase (ALP) activity and calcium deposition.

Phosphorylated CS derivatives enhance the solubility and thus the bioavailability of drugs by improving their interactions with biological membranes. This results in better absorption and a more effective therapeutic profile. Phosphorylated CS derivatives are extensively used in controlled drug delivery systems. Gogoi et al. [188] utilized phosphorylated chitosan (PCS) to prepare nanoparticles for drug delivery applications, specifically for curcumin combined with (±) alpha-lipoic acid. The study confirmed the anticancer efficacy of these PCS-based nanoparticles.

The addition of phosphate groups increases the solubility and biocompatibility of chitosan, allowing for the prolonged and sustained release of therapeutic agents. This controlled release is essential in maintaining effective drug levels over extended periods, improving patient compliance and treatment outcomes. Wei et al. [189] prepared a water-soluble derivative of PCS and evaluated its potential as a novel immune adjuvant. PCS was found to be pH-sensitive: it was water-soluble at pH < 7.0 but began to gel at pH > 7.0. Neutral PCS aqueous solutions containing ovalbumin (OVA) antigens were intramuscularly injected into test mice, forming an OVA-containing gel network for immunization. The results indicated that using a 30 mg/mL PCS-based hydrogel as a vaccine delivery system led to significantly stronger antigen-specific immune responses, including elevated levels of antigen-specific IgG antibodies, as well as the increased secretion of IFN-γ and IL-4 cytokines by splenocytes, along with the presence of memory CD^4+^ and CD^8+^ T cells. In vivo imaging and immunohistochemistry assays suggested that the enhanced immunization efficacy may have been due to the controlled release of the antigen from the injection site by the PCS gel network, providing prolonged antigen stimulation to the immune system.

Phosphorylated CS derivatives are valuable in a range of applications, including tissue regeneration, wound healing, and drug delivery systems.

#### 4.3.3. Methacrylated CS

Conjugating methyl methacrylate (MMA) with CS is an effective strategy to impart desirable properties such as non-toxicity, biodegradability, and biocompatibility to the resulting biopolymer. This modification can create highly porous microspheres with potential applications in drug release within the pharmaceutical and medical fields. CS–MMA has been explored for various uses, including as a gene and drug delivery agent, medical implants, and dental devices [190,191,192].

MMA-grafted chitosan (CS–MMA) has demonstrated significant potential as a gene delivery agent, evidenced by its effective transfection efficiency in mammalian cancer cell lines such as A549, HeLa, and HepG2. Notably, CS–MMA exhibited excellent blood compatibility, as it did not induce hemolysis in blood samples. Comparative studies revealed no significant reduction in leukocyte, erythrocyte, or platelet counts, indicating that both CS and its MMA-grafted copolymers were highly compatible with blood. Wells without materials served as the reference in these tests, further affirming the favorable biocompatibility of the CS–MMA materials. In drug delivery studies, nanoparticles of the CS–MMA biopolymer were prepared using the ionic gelation method with sodium tripolyphosphate (TPP). An in vitro drug release study of curcumin-loaded CS–MMA nanoparticles revealed the maximal entrapment efficiency of up to 68%. Additionally, the release of the drug was more rapid at a lower pH (5.0) compared to the physiological pH [97].

Liu et al. [193] utilized modified methacrylated chitosan (CS–MMA) to enhance the properties of polyetheretherketone (PEEK), which is typically limited by its biologically inert surface and poor osteointegration. They developed a PEEK composite incorporating methacrylated CS/polyhedral oligomeric silsesquioxane (CS–MMA/POSS). The study demonstrated that the CS–MMA/POSS’s bioactive microporous surface facilitated protein adsorption and apatite formation. The 3D-printed macroporous PEEK scaffold with this modification provided an improved environment for cell adhesion and proliferation, supported the osteogenic differentiation of rat bone marrow stem cells (rBMSCs), and promoted osteogenesis in vivo more effectively than untreated PEEK scaffolds. This approach shows promise in enhancing the osteointegration of PEEK implants.

Methacrylated CS derivatives are used to improve the mechanical strength, which leads to enhanced stability and prolonged drug release, ensuring a sustained therapeutic effect. They are utilized in controlled drug delivery due to their ability to form hydrogels. These hydrogels provide a controlled release environment, ensuring that drugs are released consistently, which is beneficial for chronic disease management. Methacrylated CS derivatives are highly effective also in gene delivery. These derivatives facilitate the transfer of genetic material into cells due to their enhanced interactions with cell membranes. This makes them suitable for gene therapy applications, where the precise and efficient delivery of genetic material is crucial for treatment effectiveness. Methacrylated CS derivatives are also used in developing materials for implants and dental devices because of their improved mechanical properties and stability.

#### 4.3.4. CS–Glutaraldehyde and CS–Genipin Crosslinked Polymers

CS-based systems, such as nanoparticles, microspheres, self-sustained films, membranes, and hydrogels, can be stabilized through covalent crosslinking. This chemical modification connects the polymer chains, which enhances their stability, insolubility, and mechanical properties, leading to improved physicochemical characteristics and performance [191].

Glutaraldehyde, also known as 1,5-pentanedial (OHC-(CH_2_)_3_-COH), is commonly used for protein immobilization and crosslinking due to its affordability and effectiveness in binding through amino groups [194]. Studies by Monteiro et al. [195] and Ahmed et al. [196] have examined the behaviors of CS hydrogels, focusing on the crosslinking density and swelling under laboratory conditions (25 °C and pH 7). More recently, Banafati Zadeh and Zamanian have investigated the optimal conditions for CS crosslinking in the context of bone tissue engineering [197].

Nayak et al. [198] developed zidovudine-loaded CS microspheres using a suspension crosslinking method with glutaraldehyde (GA) as the crosslinking agent. These drug-loaded microspheres achieved up to 60% entrapment efficiency and provided extended drug release for 18–24 h. Specifically, microspheres with a 1:6 drug-to-CS ratio and 35% GA crosslinking exhibited 75% release at 12 h. Infrared spectra and thermograms indicated that zidovudine remained stable in the microspheres, with no significant drug–polymer interactions observed.

pH-sensitive hydrogels and scaffolds for wound dressings were developed by crosslinking CS with genipin (GP). Additionally, CS–GP microspheres and nanoparticles were created as innovative drug delivery systems.

**Figure 32:**
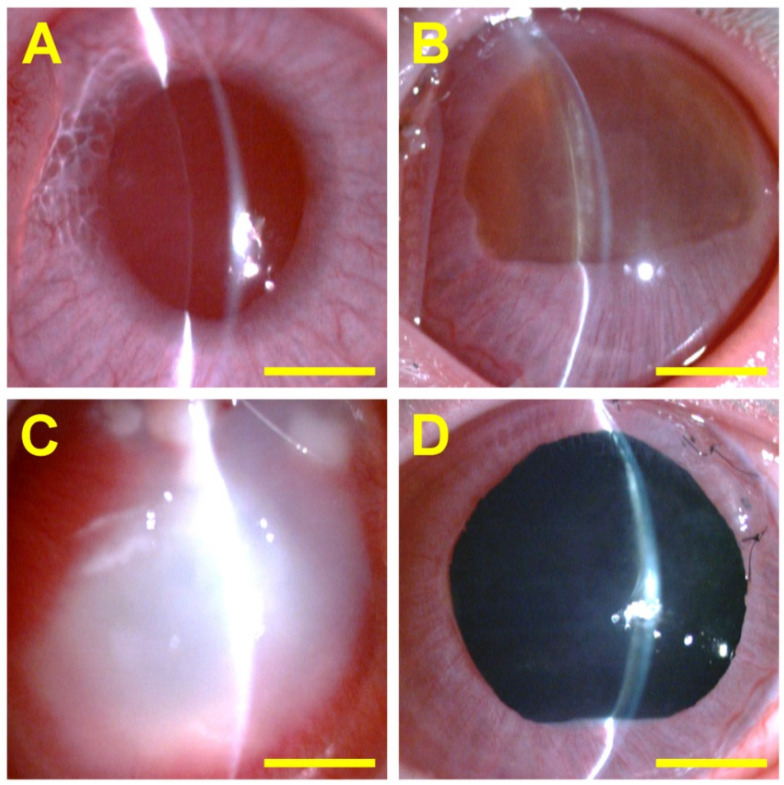
Slit-lamp biomicroscopic images of rabbit eyes, taken 24 weeks after the insertion of various CS implants into the ocular anterior chamber, are displayed. (**A**) Control (sham-operated); (**B**) CS; (**C**) CS–GTA; and (**D**) CS–GP groups. Scale bars: 3 mm [98].

Lai et al. [98] studied the in vivo biocompatibility of GP-treated chitosan (CS) using a rabbit eye model, specifically focusing on the anterior chamber. For comparison, GA-crosslinked samples were also tested. Membrane implants, 7 mm in diameter, produced from either non-crosslinked CS or chemically modified CS with approximately 80% crosslinking, were inserted into the ocular anterior chamber for 24 weeks. The implants were evaluated using slit-lamp and specular microscopy, intraocular pressure measurements, corneal thickness assessments, and interleukin-6 mRNA expression levels. Clinical observations (see Figure 32) revealed that the overall ocular scores in the CS–GT groups were relatively high. In contrast, rabbits with CS–GP implants showed no signs of ocular inflammation. Compared to non-crosslinked CS, CS–GP samples better preserved the corneal endothelial cell density and exhibited stronger anti-inflammatory effects, highlighting the advantages of the GP crosslinker. In summary, the intracameral tissue response to chemically modified CS materials was significantly influenced by the choice of crosslinking agent.

Hydrogels produced from glutaraldehyde-crosslinked CS exhibit significant swelling in water or biological fluids, making them suitable for applications such as bioactive macromolecule carriers, wound dressings, and controlled drug release.

### 4.4. Physically Crosslinked CS

Blending CS with ionic or non-ionic small or large molecules can create physically crosslinked CS. The key interactions in ionic crosslinking are Coulombic ones, occurring between the oppositely charged functional groups of interacting compounds (e.g., ammonium of CS and carboxylate, phosphate, etc., of a partner molecule), represented, for instance, by CS–tripolyphosphate or CS–alginate couples. The most significant interactions in non-ionic crosslinking are usually hydrogen bonds occurring between the -OH functional groups of interacting compounds, represented, for instance, by CS–polysaccharides (dextrin, Arabic gum, etc.), CS–polyalcohols (e.g., polyvinyl alcohol), or other couples. Of course, mixed ionic and non-ionic interactions usually occur when blending charged polysaccharide-based polymers with CS; in such cases, it is referred to as an ionic crosslink due to the stronger Coulombic interactions. In the following subsections, ionic and non-ionic crosslinked CS formulations are demonstrated for various medical applications, focusing especially on wound healing.

#### 4.4.1. Ionically Crosslinked CS

Tripolyphosphate (TPP) is a commonly used anionic crosslinker for chitosan (CS), widely investigated for the creation of microparticles, nanoparticles, membranes, hydrogels, scaffolds, and composite systems in biomedicine [109,111]. TPP is particularly valuable in bone regeneration applications due to its phosphate groups, crucial for bone mineralization. Consequently, TPP is extensively employed in developing biomimetic polymer systems for bone repair [111]. Additionally, CS–TPP micro- and nanogels have been thoroughly studied for drug, protein, and gene delivery systems [199].

Ionic gelation, a technique involving interactions between the positively charged amino groups of CS and the negatively charged groups of a polyanion-like tripolyphosphate (TPP), is commonly used to prepare CS nanoparticles. TPP is preferred as an ionic crosslinker due to its stable performance, ease of process control, and safety. CS–TPP nanoparticles are promising for biomedical applications, benefiting from their inherent biological properties, such as non-toxicity, antimicrobial activity, mucoadhesion, and hemocompatibility. Additionally, these nanoparticles can incorporate various bioactive species, including hydrophilic and hydrophobic molecules or macromolecules [200].

CS–TPP-crosslinked polymers are employed to provide the controlled release of drugs. The crosslinking with tripolyphosphate (TPP) enhances the stability and bioavailability of the encapsulated drug, leading to a more consistent therapeutic effect.

CS hydrogel-based wound dressings containing silymarin can be applied for effective burn wound treatment.

Valadi et al. [201] fabricated an alginate–chitosan hydrogel comprising silymarin and green-synthesized zinc oxide nanoparticles to enhance burn wound healing. The obtained hydrogel exhibited great water absorption, high porosity, sustainable degradation for several days, and enhanced antioxidant capabilities in the combined loaded component. The wound-healing efficacy was evaluated in a rat model of full-thickness dermal burn wounds, and in vivo studies revealed faster and superior wound healing, achieving nearly complete closure by day 21. Histopathology confirmed the improved cell growth, tissue regeneration, collagen deposition, and neovascularization.

#### 4.4.2. Non-Ionically Crosslinked CS

Chitosan-based electrospun scaffolds loaded with flaxseed can be effectively applied for the promotion of wound healing.

Doostan et al. [202] developed an electrospun polyvinyl alcohol and chitosan (PVA/CS) nanofibrous scaffold-loaded flaxseed extract that displayed remarkable antioxidant properties and could inhibit the growth of both Gram-positive and -negative bacteria compared to a free CS–PVA scaffold. In vitro studies showed that the flaxseed-loaded scaffold accelerated wound healing and achieved 100% closure of the scratched area within 48 h.

The entrapment of Thymus vulgaris essential oil in a chitosan blend hydrogel enhanced its antifungal properties, and this could be promising for the treatment of skin candidiasis or onychomycosis.

Dinu et al. [203] loaded Thymus vulgaris essential oil (EO) within physically crosslinked sponge-like cryogels by ice template-assisted freeze-drying. Their 3D cryogenically structured network was built through hydrogen bonding formed by blending CS and dextrin. The films showed both antioxidant and antifungal properties, with radical scavenging activity of 65% and a zone inhibition diameter of 40 mm for *Candida parapsilosis* fungi.

In addition, a combination of two extracts, thyme and ginger, in a CS electrospun mat was investigated to promote the healing of infected wounds and the tissue regeneration process.

Maleki et al. [204] developed a nanofibrous mat produced from polyvinyl alcohol and chitosan (CS–PVA) using the electrospinning method, incorporating thyme (*Thymus vulgaris*) and ginger (*Zingiber officinale*) extracts. The mat exhibited strong antioxidant activity and demonstrated continuous, sustained release for nearly 72 h. Additionally, it effectively inhibited the growth of both Gram-positive *S. aureus* and Gram-negative *E. coli*. Moreover, the mat significantly accelerated wound healing in bacteria-infected rats by preventing bacterial growth at the wound site. The histopathological analysis further confirmed the substantial skin regeneration, including the formation of collagen fibers and skin appendages.

Amalraj et al. [205] prepared biocomposite films based on polyvinyl alcohol, Arabic gum, and CS incorporated with black pepper and ginger essential oils via the solvent casting method. These films significantly inhibited the growth of *Bacillus cereus*, *Staphylococcus aureus*, *Escherichia coli*, and *Salmonella typhimurium*.

### 4.5. CS Derivatives in Practice and Future Challenges

Combining specific CS derivatives with various drugs can significantly alter their pharmacokinetics by improving their solubility, enhancing their bioavailability, and enabling controlled release. The main applications of CS derivatives in pharmacy and medicine are currently gene delivery, controlled drug delivery (via affected pharmacokinetics)—prolonged and sustained—targeted drug delivery, wound healing, and tissue engineering, as well as in materials for implants and dental devices. Thus, CS derivatives have diverse applications in pharmacy and medicine, each tailored using specific modifications. While there are few clinical studies, chitosan has shown minimal to no toxicity in animal models, and no significant adverse effects have been reported in healthy human volunteers. To this day, no chitosan-based drug formulation has been approved for widespread use, although chitosan is permitted in dietary products, wound dressings, and cartilage compositions [206].

Published patents and undergoing clinical studies, which are discussed in the following Section 4.5.1 and Section 4.5.2, respectively, underline the current extent of implementation of CS derivatives in pharmaceutical and medical practice and show the particular areas of their practical use. However, there remain challenges for the future application of CS and its derivatives, as discussed in Section 4.5.3.

#### 4.5.1. Patents

Recently, several patents concerning CS derivatives and their potential use in biomedical applications have been granted. For instance, US Patent No. 6,162,809 focused on the use of carboxymethylated chitosan derivatives, namely *N*,*O*-carboxymethyl CS, as drug carriers, demonstrating their value in controlled drug release systems, and US Patent No. 5,929,044 was granted for *O*-carboxymethyl chitosan, which is applied in wound healing due to its improved solubility and efficacy.

US Patent No. 6,333,041 dealt with *N*-succinyl chitosan as a carrier for the delivery of water-insoluble drugs, highlighting its ability to improve drug solubility and delivery. Another US Patent, No. 6,916,833, was granted for quaternized chitosan derivatives, emphasizing their antibacterial applications, particularly in medical device coatings and wound dressings. US Patent No. 8,304,467 explored PEGylated chitosan derivatives in enhanced drug delivery systems, especially in long-circulating drug carriers.

US Patent No. 7,744,917 was granted for *N*-trimethyl chitosan as a permeation enhancer in mucosal drug delivery systems, improving drug absorption across biological membranes. US Patent No. 8,263,098 was granted for CS–TPP nanoparticles used in oral drug delivery to enhance the bioavailability of difficult-to-absorb drugs.

These examples of patents demonstrate the real potential for the future implementation of patented CS derivatives in pharmaceutical and medical practice.

#### 4.5.2. Clinical Studies

**Figure 33:**
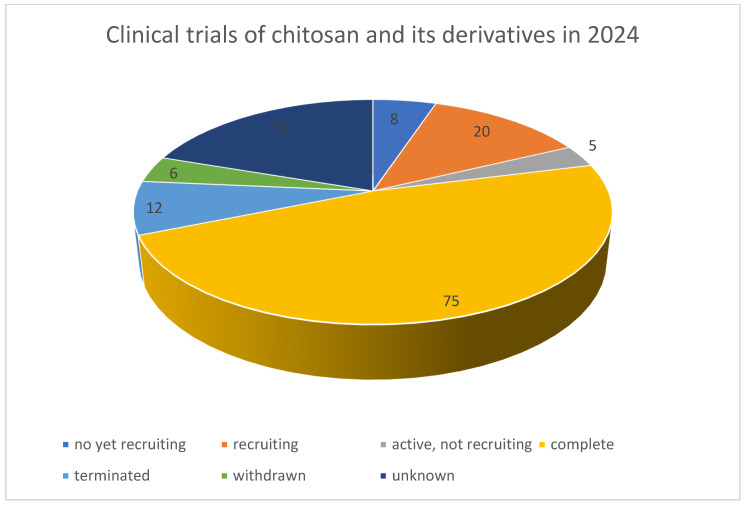
The recruitment statuses of clinical studies concerning chitosan and its derivatives in 2024. Data taken from [207].

There are currently 157 ongoing studies concerning chitosan [207]. The recruitment statuses of these studies are illustrated in Figure 33.

The purposes of the chitosan preparations and materials involved in the above-discussed clinical studies include periodontal diseases, stomatitis, tooth extraction, pulpitis, osteoarthritis, postoperative pain, vaginal bleeding and cervical pain, cesarean wounds, anticancer therapy, oral pharmaceuticals, glucose level control, hypercholesterolemia, obesity, allergies, COVID-19, coronary artery disease, dry eye syndrome, diabetic foot ulcers, pulpal necrosis, epistaxis, wounds, chronic kidney disease, atopic dermatitis, liver cirrhosis, psoriasis, bacterial infections, etc.

#### 4.5.3. Challenges and Future Perspectives

In the study of CS derivatives, there is a notable lack of clarity regarding the relationship between their structures and properties; in particular, we lack a comprehensive understanding of how structural modifications affect the material characteristics and performance of CS derivatives. Future research should prioritize systematic investigations to correlate the chemical structures, such as the degree of deacetylation, molecular weight, and functional group types, with the mechanical, biological, and physicochemical properties. This understanding will facilitate the rational design and optimization of CS derivatives for specific applications, improving their effectiveness and versatility.

Chitosan is approved for biomedical applications, but there are still significant concerns regarding its use in pharmaceuticals. The main regulatory issues arise from its sources and characterization. Key concerns include its purity, contamination with impurities, heavy metals, proteins, microbial loads, and bacterial endotoxins. The proper characterization of chitosan is crucial, as the degree of deacetylation is an important factor that influences its chemical behavior and bioadhesive properties [208].

Additionally, the scalability and standardization of chitosan production and its processing methods represent another critical gap. Since chitosan is derived from natural sources, like shrimp shells and fungal cell walls, its quality and properties vary between producers due to the lack of standardization. Regulatory approval by the European Union and US FDA for the use of chitosan and its derivatives in the biomedical field requires comprehensive material characterization, a thorough safety profile, and assurance of product consistency. While many commercial suppliers offer chitosan, the degree of deacetylation varies significantly, typically between 70% and 98%. However, most supplier specifications lack important details, such as the source of the chitosan, its microbial load, and data on its immunogenicity or toxicity. Another issue is the molecular weight. The labeling “high-, medium-, or low-molecular-weight chitosan” is not sufficient for study validation. Therefore, there is a clear need for standardized manufacturing guidelines for pharmaceutical-grade chitosan, including mandatory tests for hypersensitivity, endotoxin levels, immunogenicity, systemic toxicity, and purity, along with deacetylation.

In biomedical applications, another challenge is achieving precise control over the drug release kinetics, enhancing tissue regeneration, and promoting long-term biocompatibility. Hence, future research should focus on developing innovative strategies, such as nanotechnology-based delivery systems, biofunctionalized scaffolds, and combination therapies, to address these challenges and help to transition chitosan-based materials from the lab to clinical use.

## 5. Conclusions

Outcomes. This comprehensive study of CS and its derivatives explores and highlights the remarkable potential of these biopolymers across various fields, focusing especially on biomedicine, bioengineering, and pharmacy. CS, as a semi-synthetic polymer derived from the natural chitin of crustaceans or insects, serves as a versatile basis for various modifications in its structure. These modifications can lead to the formation of various derivatives with enhanced properties compared with the native CS. The key factor is the low solubility of the native CS in media with a physiological pH, which is enhanced to various degrees (due to the reduction in H-bonds in the CS structure) in the majority of the studied CS derivatives. Other basic or derived physicochemical properties are effectively controlled via CS derivatization too, such as an increase in complexing activity with EDTA derivatives, the tuning of the amphiphilic properties with cholic acid attached to CS, the tuning of the mechanical properties and viscosity with physically and/or chemically crosslinked CS, etc. In addition, their biocompatibility, non-toxicity, and bioactivity are usually preserved or even increased. These attributes highlight the importance of CS modification and support further studies of known CS derivatives, as well as the development of new CS derivatives.

In the development of preparation schemes for common or new CS derivatives, continuous progress and efforts toward their enhancement can be seen. Hence, besides applying pre-established synthetic procedures, innovations are apparent, utilizing new or alternative chemical agents, e.g., those that are less expensive or less toxic, those that allow mild reaction conditions, chemoselective agents, and catalyzers, as well as introducing physical effects for the enhancement of the reaction, e.g., an ultrasonic-supported method. The selection of a suitable reaction mechanism for the introduction of the required functionality into the CS structure is another option to conduct the preparation of CS derivatives more effectively. New preparation approaches seek less expensive, less toxic, and simpler methods, with green aspects, reducing the number of synthetic steps, providing reactions that are operated as a progressive one-pot process under mild conditions that do not require either an inert atmosphere or dry solvents. Such advanced chemistry and methodologies are beneficial not only due to their enhanced efficacy in the preparation of CS derivatives but also due to the possibility of providing the required selectivity concerning the chemical structure of the resulting CS derivative, such as selective *O*- and *N*-derivatization. Selective CS modification is of high interest concerning the study of the structure–activity relationships of new CS derivatives with a well-defined structure.

One of the key benefits lies in drug delivery systems, where CS derivatives demonstrate excellent biocompatibility and controlled release properties. Their ability to encapsulate a variety of drugs, protect them from degradation, and release them at targeted sites in a controlled manner makes them valuable in formulating pharmaceutical dosage forms with targeted drug release (specifically provided via TMC-coated gold NPs for cancer targeting, PEGylated CS NPs containing collagen for hepatic targeting, and TMC and bufalin-based NPs for lung-specific tumor targeting). CS derivatives can alter the pharmacokinetics of drugs and result in either prolonged drug delivery (carboxylated, quaternized, or PEGylated CS derivatives) or sustained drug release (acylated, succinylated, benzoylated, methacrylated, TPP-crosslinked, and CS–EDTA derivatives). They act by increasing the solubility of the drugs (alkylated, carboxylated, quaternized, succinylated, phosphorylated, phthaloylated, PEGylated, sulfonated, CS–CD derivatives, and cholic acid–CS derivatives) or their stability (*N*-phthaloylated, PEGylated, CS–EDTA, TPP-crosslinked, and cyclic structure–CS derivatives) and thus increase the bioavailability of drugs and their therapeutic efficacy. In addition, they can be used for the better absorption of hydrophobic drugs (benzoylated, acylated, PEGylated, and cholic acid–CS derivatives). Some specific CS derivatives create excellent carriers for gene delivery (methacrylated, alkylated, quaternized, and carboxyalkylated CS derivatives).

Furthermore, the mucoadhesive nature of CS derivatives is advantageous in drug delivery to mucosal surfaces, allowing for prolonged contact times and improved absorption. This is particularly relevant for mucosal drug delivery in areas such as the gastrointestinal tract and nasal passages. Thiolated CS derivatives, in addition to better mucoadhesion, show permeation retention, the inhibition of efflux pumps, and in situ gelling, which makes them advantageous for oral DDS. For gastroresistant DDS, *N*-phthaloylated derivatives are suitable because they can protect the drug against the acidic pH. The PEGylation of CS and succinylation of CS leads to prolonged circulation in the bloodstream. PEGylated derivatives even lower the immunogenicity of the incorporated drugs. Some of the CS derivatives, such as cholic acid derivatives and quaternized and thiolated derivatives, enhance the gastrointestinal absorption of drugs.

CS derivatives also play a role in wound healing and tissue engineering/regeneration. Their hemostatic, anticoagulation, antiaggregation, and antimicrobial properties and biocompatibility contribute to their use in wound dressings, drug-releasing scaffolds, or medical gauzes (acylated, alkylated, phosphorylated, carboxylated, sulfated, and succinyl CS derivatives), promoting healing and reducing the infection risks. CS derivatives represent also advanced materials for implants and dental devices (methacrylated CS derivatives).

Limitations. Although chitosan and its derivatives possess very low to zero toxicity, to this day, no chitosan-based drug formulation has been approved for widespread use. There are several patents and clinical studies concerning chitosan derivatives in pharmaceutical preparations; however, some limitations still prevent their widespread use in pharmaceutical practice. One of these issues is the purity of chitosan (heavy metal content, bacterial endotoxins, microbial purity, etc.); another problem is the lack of standardized chitosan (degree of deacetylation, molecular weight data, structural variability within a derivatization scheme, etc.). Addressing these issues is one of the major challenges for the acceptance of CS and its derivatives as components in drug formulations.

Conclusions. Indeed, CS derivatives have potential for diverse applications in pharmacy and medicine, each tailored using specific modifications. The adaptability of CS derivatives positions them as valuable assets in the pharmaceutical industry, offering innovative solutions for drug delivery and therapeutic interventions, and they are worthy of further investigation and implementation in practice. Currently, there are several areas in which CS derivatives have achieved a relatively high degree of practical implementation: CS is permitted in dietary products, wound dressings, and cartilage compositions. Despite these promising attributes and several successful applications of CS and its derivatives, challenges like the standardization/validation of the production processes (at all stages, including CS, CS derivatives, and CS nanoparticles) and ensuring their cost-effectiveness (traditional preparation schemes vs. novel synthetic approaches, instrumental support such as microfluidics, etc.) still need to be addressed for their widespread practical implementation. Moreover, future research should prioritize systematic investigations to correlate their chemical structures, such as the degree of deacetylation, molecular weight, and functional group types, with their mechanical, biological, and physicochemical properties, in order to facilitate the rational design and optimization of CS derivatives for a given purpose.

## Figures and Tables

**Table 1:** Alkylated CS derivatives: properties, biological activity, methods of preparation, and weak and strong points determining their practical use.

CS Derivative	Formula	Physical Properties	Biological Properties	Preparation Method	Advantages/Limitations/Potential Use	References
**Alkylated CS Derivatives**						
Alkylated derivatives (in general)	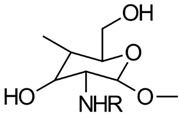	-solubility is lowered when the alkyl chain is too long -soluble at a broad range of pH values (2–12); strong mucoadhesion; decreased TEER; increased paracellular permeability of basic or neutral macromolecules	-biocompatibility -biodegradability	-alkylation reaction with alkyl halides under basic conditions with the introduction of an alkyl group on the N and O atoms of CS	-strong aggregation with anionic macromolecules such as heparin -coagulation and antibacterial -DNA delivery with dodecyl CS -increasing entry into cells facilitated by hydrophobic interactions and easier unpacking of DNA from alkylated CS carriers -antibacterial properties -increased cell entry	[6,14,15,16]
Carboxyalkylated CS	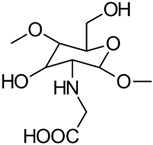	-amphoteric nature -water-soluble -film- and gel-forming abilities -solubility depends on pH -insoluble at pH 3–7 (depending on the degree of substitution) due to its polyampholytic nature	-biodegradability -biocompatibility -bioactivity -non-toxicity	*N*,*O*-CMC NaOH, chloroacetic acid, isopropanol, 50 °C *O*-CMC same but 55 °C and strongly alkaline *N*-CMC Glyoxylic acid, NaBH_4_, pH = 3, 2–4, CH_3_COOH/NaOH, 60 °C *N*,*N*-CMC Same as *N*-CMC but pH = 2–3 Amine–glyoxylic = 1:9	-antibacterial, anticancer, antitumor, antifungal, antioxidant -drug/gene therapy, targeted/controlled release of therapeutics -wound healing, tissue engineering, and bioimaging applications -protein drug delivery systems as super porous hydrogels, pH-sensitive hydrogels, crosslinked hydrogels	[17,18,19,20,23]
Quaternized derivatives	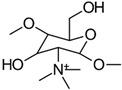	-cationic derivatives -water-soluble at neutral pH -*N*,*N*,*N*-trimethyl CS chloride (TMC) increases aqueous solubility than CS -soluble at a wide range of pH values	-biocompatibility -biodegradability -mucoadhesion	-direct quaternary ammonium substitution -epoxy derivative open loop -*N*-alkylation	-antifungal, antibacterial, antituberculosis -enzyme inhibition -permeation enhancers -gene transfection and delivery -good moisture retention and absorption -mucoadhesion decreases with increased degree of quaternization -with an increasing degree of quaternization, intrinsic viscosity decreases -pH 7.4 CS and salts failed to increase the permeability -absorption enhancer for intestinal lumen with pH close to its pKa -TMC: collecting and delivering negatively charged DNA/genes -better than plain CS -quaternized CS has an increased hydroxyl radical scavenging activity in comparison to other CS -pH-sensitive targeting -DNA delivery properties	[1,19]

**Table 2:** Acylated CS derivatives: properties, biological activity, methods of preparation, and weak and strong points determining their practical use.

Formula	Physical Properties	Biological Properties	Preparation Method	Advantages/Limitations/Potential Use	References
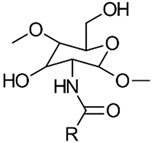	-*O*-acylated CS is lipid-soluble and dissolved in non-polar solvents—pyridine and chloroform—while *N*-acylated CS improves water solubility -The length of the side chain is proportional to the crystallinity, and a longer side chain results in higher crystallinity and lower relative solubility	-biocompatibility -non-toxicity -biodegradability	-CS dissolved in 1% acetic acid–methanol at 1:1. -neutralization before the dropwise addition of acid anhydride -solution left overnight and neutralized before precipitation with a large quantity of acetone -centrifugation at 5000 rpm for 2 min at 25 °C and the precipitate was washed with excess methanol and dried overnight under a vacuum	-anticoagulability and blood compatibility -can be used as a carrier or sustained-release agent in pharmaceutical applications -*O*-acylated CS is used in the films of fibers or polymeric materials to enhance the hydrophobicity and stability of the material; *N*-acylated CS can be used as a carrier or a sustained-release agent in the delivery of drugs and can also be used as a material additive in biological scaffolds	[6,25,26,28]

**Table 3:** Succinylated CS derivatives: properties, biological activity, methods of preparation, and weak and strong points determining their practical use.

Formula	Physical Properties	Biological Properties	Preparation Method	Advantages/Limitations/Potential Use	References
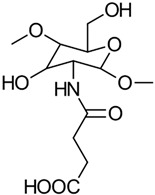	-water-soluble -pH-sensitive -insolubility at pH 4, 5–6, and 8 owing to the isoelectric point, which exists at equimolar numbers of -NH_3_^+^ and -COO^−^ groups in the molecule	-biocompatibility, low toxicity, and long-term retention in the body	-inclusion of succinyl groups on the *N*-termini of the glucosamine units of CS (succinic anhydride, L-lactic acid, methanol, pH = 6–7 for 24 h)	-anti-inflammatory, antibacterial, antimicrobial, anticoagulating, and aggregating properties -moisture retention ability -lactosaminated *N*-succinyl CS and its fluorescein thiocarbonyl derivative can be used as liver-specific drug carriers in mice through asialoglycoprotein receptors -wound-healing activity	[32,33,34]

**Table 4:** Benzoylated CS derivatives: properties, biological activity, methods of preparation, and weak and strong points determining their practical use.

Formula	Physical Properties	Biological Properties	Preparation Method	Advantages/Limitations/Potential Use	References
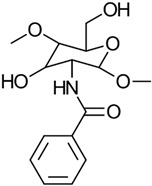	-better foam formation and foaming stability -soluble in DMSO, DMF, and acetone -insoluble in THF and ethanol	-biodegradability -biocompatibility	-benzoylation of CS -partial acylation of CS, benzoyl chloride, and acetic acid under high-intensity ultrasound	-antifungal and antibacterial activity stronger than CS -similar thermal stability to CS -BBTU-CS-4 (4,4-(5,5′ carbonylbis(1,3-dioxoisoindoline-5,2-diyl))dibenzoyl isothiocyanate) showed promising potential as an anti-H. pylori and selective anti-inflammatory agent -in comparison with the COX inhibitor celecoxib, BBTU-CS showed inhibition activity towards COX enzymes, with selective inhibition towards COX-2	[35,38,39]

**Table 5:** Phthaloylated CS derivatives: properties, biological activity, methods of preparation, and weak and strong points determining their practical use.

Formula	Physical Properties	Biological Properties	Preparation Method	Advantages/Limitations/Potential Use	References
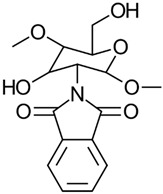	-soluble in organic solvents, film formability, flexibility -increased permeance and hydrophilicity	-biocompatibility -biodegradability	-CS with phthalic anhydride in *N*,*N*-dimethylformamide (DMF) at 130 °C and *O*-(3,6-hydroxyethyl) CS was produced using chlorohydrins as a grafting agent and hydrazine hydrate as a reductant	-*N*-phthaloyl CS derivatives have higher reactivity than *N*,*O*-phthaloylated CS derivatives -enhanced drug solubilization	[40,41,42,43]

**Table 6:** Thiolated CS derivatives: properties, biological activity, methods of preparation, and weak and strong points determining their practical use.

Formula	Physical Properties	Biological Properties	Preparation Method	Advantages/Limitations/Potential Use	References
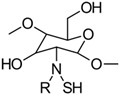	-cohesive properties for prolonged controlled release -hydrophilic macromolecules	-adhesion to biological surfaces -biocompatibility -biodegradability -enzyme inhibition -antioxidative properties	-immobilization of thiol-bearing moieties in the 2-position of glucosamine -using thiolating agents bearing thiol groups like cysteine, thioglycolic acid (TGA), 2-iminothiolane or 4-thiobutylamidine (TBA), *N*-acetyl cysteine, isopropyl-S-acetylthioacetimidate, and glutathione -oxidation of thiol groups during synthesis can be avoided by performing the reaction under inert conditions	-controlled release of covalently bound active pharmaceutical ingredients -enhanced API permeation due to opened tight junctions caused by the interaction of thiolated CS with cysteine-bearing membrane receptors and enzymes -permeation-enhancing effects, ability to inhibit efflux pumps, and in situ gelling properties -improved permeability -controllable drug release	[45,46,47,48,49,50,51,52]

**Table 7:** PEGylated CS derivatives: properties, biological activity, methods of preparation, and weak and strong points determining their practical use.

Formula	Physical Properties	Biological Properties	Preparation Method	Advantages/Limitations/Potential Use	References
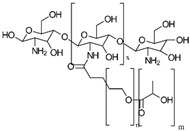	-prolonged body residence time -soluble in water and organic solvents (DMF, DMSO)	-biocompatible -no toxicity, antigenicity, and immunogenicity	1. deacetylation of CS 2. using DMF for preparation of phthaloyl CS 3. SOCl2 and pyridine-prepared chlorinated phthaloyl CS intermediate 4. NaH and tetrahydrofuran (THF) PEGylated phthaloyl CS 5. deprotection of PEGylated phthaloyl CS by hydrazine monohydrate and final product = PEGylated CS 6.-grafting hydrophilic PEG onto the backbone of CS	-antimicrobial activity -reduced renal clearance and limited toxicity -increases the stability of the drug against enzymatic degradation -low cytotoxicity, higher ductility, and body fluid stability make this derivative attractive for drug delivery, hydrogels, and nanotechnology	[53,56,57,58]

**Table 8:** CS–EDTA derivatives: properties, biological activity, methods of preparation, and weak and strong points determining their practical use.

Formula	Physical Properties	Biological Properties	Preparation Method	Advantages/Limitations/Potential Use	References
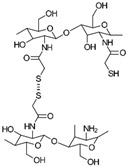	-low transfection efficacy in comparison to viral transfection, reducing the cationic charge of the polymer, thus reducing the strength of binding to poly-anionic plasmid DNA -crosslinked CS–EDTA polymer could produce stabilized particles with efficient release	-mucoadhesive properties -biocompatibility -biodegradability	-conjugates have been generated by carbodiimide condensation with acids or by interacting with anhydrides of complexing agent EDTA -covalent crosslinking can be achieved with a smaller amount of EDTA during the coupling reaction—one EDTA molecule is bound to more than only one amino group of CS	-better mucoadhesion than unmodified CS -inhibits Zn- and Co-dependent proteases including carboxypeptidase A and aminopeptidase N -no Ca-dependent serine protease inhibition -can be used as a carrier matrix where the release of drugs can be controlled -antimicrobial activity	[63,64,65]

**Table 9:** CS–CD derivatives: properties, biological activity, methods of preparation, and weak and strong points determining their practical use.

Formula	Physical Properties	Biological Properties	Preparation Method	Advantages/Limitations/Potential Use	References
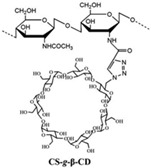	-hydrophilic external surface and hydrophobic internal cavity -inclusion of small organic molecules -cyclodextrins enhance solubility and dissolution of poorly water-soluble drugs -enhanced wettability	-non-toxicity -mucoadhesion	1. reductive amination -a solution of CS in acetic acid/methanol reacts with an aldehyde-containing CD derivative in the presence of NaCNBH_3_ 2. amidation of CDs modified with a carboxylic group with the amino groups of CS 3. nucleophilic substitution of halides or tosyl groups by CS amino groups 4. anchoring β-cyclodextrin onto CS by click chemistry using Huisgen cycloaddition reaction	-electrostatic interactions between chitosan-*g*-CDs and insulin allowed strong binding at a wide range of pH values -potential to be applied in the delivery of peptides and proteins as an efficient delivery carrier -improving cell proliferation -orthopedic applications	[68,69,70,71,72]

**Table 10:** Crown ether–CS derivatives: properties, biological activity, methods of preparation, and weak and strong points determining their practical use.

Formula	Physical Properties	Biological Properties	Preparation Method	Advantages/Limitations/Potential Use	References
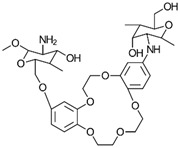	-do not dissolve in general organic solvents (such as dimethysulfoxide, formamide, and dimethylformamide) -can be powdered and are thus better adsorbents than simple CS	-biocompatibility -biodegradability -mucoadhesion	-reaction of 4,4′-diformyldibenzo-18-c-6 crown ether with crosslinked CS	-bacteriostatic potential -good complexing selectivity for metal ions because of the synergistic effect of a high molecular weight -crown-ether-bound CS has good absorption capacity for Pd^2+^, Au^3+^, and Ag^+^ ions and high selectivity for the adsorption of Pd^2+^ in the presence of Cu^2+^ and Hg^2+^ -enhanced wettability -orthopedic applications—smart drug delivery systems	[73,74,75]

**Table 11:** Sulfonated CS derivatives: properties, biological activity, methods of preparation, and weak and strong points determining their practical use.

Formula	Physical Properties	Biological Properties	Preparation Method	Advantages/Limitations/Potential Use	References
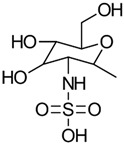	-enhanced water solubility -water-soluble amphoteric polymer -flocculant, separating agent -for immunogen carriers, water-soluble drug carriers	-biocompatibility -biodegradability -hemocompatibility	-hydrothermal grafting reaction using 4-formyl-1,3-benzene disulfonate and glutaraldehyde as a crosslinker reagent -CS with chlorosulfonic acid (ClHSO_3_) in pyridine or with sulfur trioxide (SO_3_) in dimethylformamide (DMF)	-selective antimicrobial activity, antifungal, antibacterial, anticoagulant -stronger antibacterial activity against Gram-negative bacteria -antiviral activity -a structure similar to heparin that is studied for inexpensive anticoagulant activity	[76,78,82,83,84,85]

**Table 12:** Phosphorylated CS derivatives: properties, biological activity, methods of preparation, and weak and strong points determining their practical use.

Formula	Physical Properties	Biological Properties	Preparation Method	Advantages/Limitations/Potential Use	References
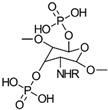	-good water solubility -good application prospects as an excellent rare earth complexant	-biocompatibility -biodegradability -osteoinductive properties	-H_3_PO_4_/P_2_O_5_/Et_3_PO_4_/hexanol method -Kabachnik–Fields reaction (CS with phosphorous acid and formaldehyde sequentially or simultaneously in aqueous acidic medium provides water-soluble *N*-mono- and di-phosphonicmethylene CS)	-bactericidal and osteoinductive properties -less thermal stability and crystallinity than CS -CS extended with phosphorous-containing groups like the -COOH group of carboxymethyl CS made to react with -NH_2_ of phosphatidylethanolamine, affording an amphiphilic polymer, which was investigated regarding its feasibility as a delivery carrier for the transfection of the hydrophobic drug ketoprofen	[90,91,92,93]

**Table 13:** Methacrylated CS derivatives: properties, biological activity, methods of preparation, and weak and strong points determining their practical use.

Formula	Physical Properties	Biological Properties	Preparation Method	Advantages/Limitations/Potential Use	**References**
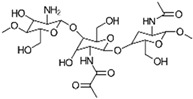	-water-soluble -amorphous network and porous, while CS has a non-porous and flat lamellar phase surface	-biodegradability -biocompatibility	-Reacting CS with methacrylic anhydride at various molar ratios -Michael’s addition reaction	-use of low-molecular-weight PEGDA results in weaker mucoadhesion -good transfection efficiency -simple and viable synthetic strategy to generate drug carriers with greater mucoadhesive properties -methacrylated CS dosage forms offer prolonged drug residence times in the bladder for bladder cancer treatment	[94,95,96,97]

**Table 14:** CS–TPP derivatives: properties, biological activity, methods of preparation, and weak and strong points determining their practical use.

Formula	Physical Properties	Biological Properties	Preparation Method	Advantages/Limitations/Potential Use	References
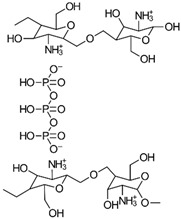	-higher TPP-to-CS ratios reduced the ζ potential and increased the compactness of the particles -the physical stability of CS nanoparticles crosslinked with TPP is affected by the ionic strength, the CS concentration, and the TPP-to-CS ratio	-biocompatible -non-toxic -biodegradable -cytocompatibility	-ionic gelation for preparation of nanoparticles	-showed superior stability upon storage -improved poor solubility of non-crosslinked CS in aqueous media -TPP nanoparticles prepared and stored in saline solvents were stable for one month -suitable for tissue engineering applications -improving mechanical properties, insolubility, and stability of polymers -gels for wound dressing -ocular implants	[98,99,100,101,102,103,104,105,106,107,108,109,110,111]

## Data Availability

No new data were created or analyzed in this study.

## References

[1] Freitas E.D., Moura C.F., Kerwald J., Beppu M.M. (2020). An Overview of Current Knowledge on the Properties, Synthesis and Applications of Quaternary Chitosan Derivatives. Polymers.

[2] Harugade A., Sherje A.P., Pethe A. (2023). Chitosan: A review on properties, biological activities and recent progress in biomedical applications. React. Funct. Polym..

[3] Wang J., Wang L., Yu H., Yu H., Zain-ul-Abdin, Chen Y., Chen Q., Zhou W., Zhang H., Chen X. (2016). Recent progress on synthesis, property and application of modified chitosan: An overview. Int. J. Biol. Macromol..

[4] Alqahtani N.F. (2024). Functionalized imidazolium ionic liquids-modified chitosan materials: From synthesis approaches to applications. React. Funct. Polym..

[5] Mohite P., Shah S.R., Singh S., Rajput T., Munde S., Ade N., Prajapati B.G., Paliwal H., Mori D., Dudhrejiya A.V. (2023). Chitosan and chito-oligosaccharide: A versatile biopolymer with endless grafting possibilities for multifarious applications. Front. Bioeng. Biotechnol..

[6] Wang W., Meng Q., Li Q., Liu J., Zhou M., Jin Z., Zhao K. (2020). Chitosan Derivatives and Their Application in Biomedicine. Int. J. Mol. Sci..

[7] Abourehab M.A.S., Pramanik S., Abdelgawad M.A., Abualsoud B.M., Kadi A., Ansari M.J., Deepak A. (2022). Recent Advances of Chitosan Formulations in Biomedical Applications. Int. J. Mol. Sci..

[8] Shrestha R., Thenissery A., Khupse R., Rajashekara G. (2023). Strategies for the Preparation of Chitosan Derivatives for Antimicrobial, Drug Delivery, and Agricultural Applications: A Review. Molecules.

[9] Petroni S., Tagliaro I., Antonini C., D’Arienzo M., Orsini S.F., Mano J.F., Brancato V., Borges J., Cipolla L. (2023). Chitosan-Based Biomaterials: Insights into Chemistry, Properties, Devices, and Their Biomedical Applications. Mar. Drugs.

[10] Pathak K., Misra S.K., Sehgal A., Singh S., Bungau S., Najda A., Gruszecki R., Behl T. (2021). Biomedical Applications of Quaternized Chitosan. Polymers.

[11] Rout S.R., Kar B., Pradhan D., Biswasroy P., Haldar J., Rajwar T.K., Sarangi M.K., Rai V.K., Ghosh G., Rath G. (2023). Chitosan as a potential biomaterial for the management of oral mucositis, a common complication of cancer treatment. Pharm. Dev. Technol..

[12] Xia Y., Wang D., Liu D., Su J., Jin Y., Wang D., Han B., Jiang Z., Liu B. (2022). Applications of Chitosan and its Derivatives in Skin and Soft Tissue Diseases. Front. Bioeng. Biotechnol..

[13] Confederat L.G., Tuchilus C.G., Dragan M., Sha’at M., Dragostin O.M. (2021). Preparation and Antimicrobial Activity of Chitosan and Its Derivatives: A Concise Review. Molecules.

[14] Jin H., Wang Z. (2022). Advances in Alkylated Chitosan and Its Applications for Hemostasis. Macromol.

[15] Burr S.J., Williams P.A., Ratcliffe I. (2018). Synthesis of cationic alkylated chitosans and an investigation of their rheological properties and interaction with anionic surfactant. Carbohydr. Polym..

[16] Chen Z., Yao X., Liu L., Guan J., Liu M., Li Z., Yang Y., Huang S., Wu J., Tian F. (2017). Blood coagulation evaluation of N-alkylated chitosan. Carbohydr. Polym..

[17] Setyawati A., Kartini I., Pranowo D., Muiz D.L.J., Hasyati S. (2017). Synthesis and Characterization of Biodegradable Film Chitosan and Carboxymethyl Chitosan to Substitute Silver Wound Healer Plaster. Orient J. Chem..

[18] Muzzarelli R.A.A. (1988). Carboxymethylated chitins and chitosans. Carbohydr. Polym..

[19] Jiao Z., Huo Q., Lin X., Chu X., Deng Z., Guo H., Peng Y., Lu S., Zhou X., Wang X. (2022). Drug-free contact lens based on quaternized chitosan and tannic acid for bacterial keratitis therapy and corneal repair. Carbohydr. Polym..

[20] Curti E., De Britto D., Campana-Filho S.P. (2003). Methylation of Chitosan with Iodomethane: Effect of Reaction Conditions on Chemoselectivity and Degree of Substitution. Macromol. Biosci..

[21] de Britto D., Assis O.B.G. (2007). A novel method for obtaining a quaternary salt of chitosan. Carbohydr. Polym..

[22] Rúnarsson Ö.V., Malainer C., Holappa J., Sigurdsson S.T., Másson M. (2008). tert-Butyldimethylsilyl O-protected chitosan and chitooligosaccharides: Useful precursors for N-modifications in common organic solvents. Carbohydr. Res..

[23] Shariatinia Z. (2018). Carboxymethyl chitosan: Properties and biomedical applications. Int. J. Biol. Macromol..

[24] Mourya V.K., Inamdar N.N. (2008). Chitosan-modifications and applications: Opportunities galore. React. Funct. Polym..

[25] Nanda B., Manjappa A.S., Chuttani K., Balasinor N.H., Mishra A.K., Murthy R.S.R. (2019). Acylated chitosan anchored paclitaxel loaded liposomes: Pharmacokinetic and biodistribution study in Ehrlich ascites tumor bearing mice. Int. J. Biol. Macromol..

[26] Zhang Z., Jin F., Wu Z., Jin J., Li F., Wang Y., Wang Z., Tang S., Wu C., Wang Y. (2017). O-acylation of chitosan nanofibers by short-chain and long-chain fatty acids. Carbohydr. Polym..

[27] Sashiwa H., Kawasaki N., Nakayama A., Muraki E., Yamamoto N., Arvanitoyannis I., Zhu H., Aiba S. (2002). Chemical Modification of Chitosan 121:Synthesis of Organo-soluble Chitosan Derivatives toward Palladium Absorbent for Chemical Plating. Chem. Lett..

[28] Tiew S.X., Misran M. (2017). Encapsulation of salicylic acid in acylated low molecular weight chitosan for sustained release topical application. J. Appl. Polym. Sci..

[29] Bashir S., Teo Y.Y., Ramesh S., Ramesh K., Khan A.A. (2015). N-succinyl chitosan preparation, characterization, properties and biomedical applications: A state of the art review. Rev. Chem. Eng..

[30] Aiping Z., Tian C., Lanhua Y., Hao W., Ping L. (2006). Synthesis and characterization of N-succinyl-chitosan and its self-assembly of nanospheres. Carbohydr. Polym..

[31] Zhang C., Ping Q., Zhang H., Shen J. (2003). Synthesis and characterization of water-soluble O-succinyl-chitosan. Eur. Polym. J..

[32] Dubashynskaya N.V., Bokatyi A.N., Dobrodumov A.V., Kudryavtsev I.V., Trulioff A.S., Rubinstein A.A., Aquino A.D., Dubrovskii Y.A., Knyazeva E.S., Demyanova E.V. (2023). Succinyl Chitosan-Colistin Conjugates as Promising Drug Delivery Systems. Int. J. Mol. Sci..

[33] Caddeo C., Pons R., Carbone C., Fernàndez-Busquets X., Cardia M.C., Maccioni A.M., Fadda A.M., Manconi M. (2017). Physico-chemical characterization of succinyl chitosan-stabilized liposomes for the oral co-delivery of quercetin and resveratrol. Carbohydr. Polym..

[34] Thao N.T.T., Wijerathna H.M.S.M., Kumar R.S., Choi D., Dananjaya S.H.S., Attanayake A.P. (2021). Preparation and characterization of succinyl chitosan and succinyl chitosan nanoparticle film: In vitro and in vivo evaluation of wound healing activity. Int. J. Biol. Macromol..

[35] Cai J., Dang Q., Liu C., Fan B., Yan J., Xu Y., Li J. (2015). Preparation and characterization of N-benzoyl-O-acetyl-chitosan. Int. J. Biol. Macromol..

[36] Atrees M.S., Metwally E., Demerdash M., Salem H. (2016). Sorption behavior of Pr and Nd upon chitosan benzoyl thiourea derivatives. J. Radiat. Res. Appl. Sci..

[37] Sabarudin A., Oshima M., Noguchi O., Motomizu S. (2007). Functionalization of chitosan with 3-nitro-4-amino benzoic acid moiety and its application to the collection/concentration of molybdenum in environmental water samples. Talanta.

[38] Lee D., Quan Z.S., Lu C., Jeong J.A., Song C., Song M.-S., Chai K.Y. (2012). Preparation and Physical Properties of Chitosan Benzoic Acid Derivatives Using a Phosphoryl Mixed Anhydride System. Molecules.

[39] Mohamed N.A., El-Ghany N.A.A., Abdel-Aziz M.M. (2021). Synthesis, characterization, anti-inflammatory and anti-Helicobacter pylori activities of novel benzophenone tetracarboxylimide benzoyl thiourea cross-linked chitosan hydrogels. Int. J. Biol. Macromol..

[40] Kurita K., Ikeda H., Shimojoh M., Yang J. (2007). *N*-Phthaloylated Chitosan as an Essential Precursor for Controlled Chemical Modifications of Chitosan: Synthesis and Evaluation. Polym. J..

[41] Aiedeh K., Taha M.O. (1999). Synthesis of Chitosan Succinate and Chitosan Phthalate and Their Evaluation as Suggested Matrices in Orally Administered, Colon-Specific Drug Delivery Systems. Arch. Pharm..

[42] Permadi R., Rizal V., Suk E.H., Misran M. (2020). Synthesis and Characterization of Acylated Low Molecular Weight Chitosan and Acylated Low Molecular Weight Phthaloyl Chitosan. Sains Malays..

[43] Kurita K., Ikeda H., Yoshida Y., Shimojoh M., Harata M. (2002). Chemoselective protection of the amino groups of chitosan by controlled phthaloylation: Facile preparation of a precursor useful for chemical modifications. Biomacromolecules.

[44] Alkabli J. (2022). Progress in preparation of thiolated, crosslinked, and imino-chitosan derivatives targeting specific applications. Eur. Polym. J..

[45] Federer C., Kurpiers M., Berkop-Schnurch A. (2021). Thiolated Chitosans: A Multi-talented Class of Polymers for Various Applications. Biomacromolecules.

[46] Ways T.M.M., Lau W.M., Khutoryanskiy V.V. (2018). Chitosan and Its Derivatives for Application in Mucoadhesive Drug Delivery Systems. Polymers.

[47] Bernkop-Schnürch A., Schwarz V., Steininger S. (1999). Polymers with thiol groups: A new generation of mucoadhesive polymers?. Pharm. Res..

[48] Bernkop-Schnürch A., Hornof M., Guggi D. (2004). Thiolated chitosans. Eur. J. Pharm. Biopharm..

[49] Kafedjiiski K., Hoffer M., Werle M., Bernkop-Schnürch A. (2006). Improved synthesis and in vitro characterization of chitosan− thioethylamidine conjugate. Biomaterials.

[50] Lal S., Arora S., Kumar V., Rani S., Sharma C., Kumar P. (2018). Thermal and biological studies of Schiff bases of chitosan derived from heteroaryl aldehydes. J. Therm. Anal. Calorim..

[51] Liu X., Li X., Zhang R., Wang L., Feng Q. (2021). A novel dual microsphere based on water-soluble thiolated chitosan/mesoporous calcium carbonate for controlled dual drug delivery. Mater. Lett..

[52] Wibel R., Braun D.E., Hämmerle L., Jörgensen A.M., Knoll P., Salvenmoser W., Steinbring C., Bernkop-Schnurch A. (2021). In Vitro Investigation of Thiolated Chitosan Derivatives as Mucoadhesive Coating Materials for Solid Lipid Nanoparticles. Biomacromolecules.

[53] Peng H., Xiong H., Li J., Chen L., Zhao Q. (2010). Methoxy poly(ethylene glycol)-grafted-chitosan based microcapsules: Synthesis, characterization and properties as a potential hydrophilic wall material for stabilization and controlled release of algal oil. J. Food Eng..

[54] Wang H., Zhao P., Liang X., Gong X., Song T., Niu R., Chang J. (2010). Folate-PEG coated cationic modified chitosan—Cholesterol liposomes for tumor-targeted drug delivery. Biomaterials.

[55] Hu F.Q., Meng P., Dai Y.Q., Du Y.Z., You J., Wei X.H., Yuan H. (2008). PEGylated chitosan-based polymer micelle as an intracellular delivery carrier for anti-tumor targeting therapy. Eur. J. Pharm. Biopharm..

[56] Casettari L., Vllasaliu D., Castagnino E., Stolnik S., Howdle S., Illium L. (2012). PEGylated chitosan derivatives: Synthesis, characterizations and pharmaceutical applications. Prog. Polym. Sci..

[57] Luo Q., Gao H., Peng L., Liu G., Zhang Z. (2016). Synthesis of PEGylated chitosan copolymers as efficiently antimicrobial coatings for leather. J. Appl. Polym. Sci..

[58] Malhotra M., Lane C., Tomaro-Duchesneau C., Saha S., Prakash S. (2011). A novel method for synthesizing PEGylated chitosan nanoparticles: Strategy, preparation, and in vitro analysis. Int. J. Nanomed..

[59] Chae S.Y., Son S., Lee M., Jang M.K., Nah J.W. (2005). Deoxycholic acid-conjugated chitosan oligosaccharide nanoparticles for efficient gene carrier. J. Control. Release.

[60] Ding Y., Cui W., Vara Prasad C.V.N.S., Wang B. (2021). Design and Synthesis of Lactose, Galactose and Cholic Acid Related Dual Conjugated Chitosan Derivatives as Potential Anti Liver Cancer Drug Carriers. Polymers.

[61] Jain A., Gubalke A. (2013). A New Horizon in Modifications of Chitosan: Synthesis and Applications. Crit. Rev. Ther. Drug Carr. Syst..

[62] El-Sharif A.A., Hussain M.H. (2011). Chitosan-EDTA new combination is a promising candidate for treatment of bacterial and fungal infections. Curr. Microbiol..

[63] Loretz B., Bernkop-Schnürch A. (2006). In vitro evaluation of chitosan-EDTA conjugate polyplexes as a nanoparticulate gene delivery system. AAPS J..

[64] Bernkop-Schnürch A., Paikl C., Valenta C. (1997). Novel bioadhesive chitosan-EDTA conjugate protects leucine enkephalin from degradation by aminopeptidase N. Pharm. Res..

[65] Mikušová V., Mikuš P. (2021). Advances in Chitosan-Based Nanoparticles for Drug Delivery. Int. J. Mol. Sci..

[66] Auzély-Velty R., Rinaudo M. (2001). Chitosan Derivatives Bearing Pendant Cyclodextrin Cavities: Synthesis and Inclusion Performance. Macromolecules.

[67] Venter J.P., Kotzé A.F., Auzély-Velty R., Rinaudo M. (2006). Synthesis and evaluation of the mucoadhesivity of a CD-chitosan derivative. Int. J. Pharm..

[68] Mahmoud A.A., El-Feky G.S., Kamel R., Awad G.E.A. (2011). Chitosan/sulfobutylether-β-cyclodextrin nanoparticles as a potential approach for ocular drug delivery. Int. J. Pharm..

[69] Vega E., Egea M.A., Calpena Campmany A.C., Espina Garcia M., Garcia M.L. (2012). Role of hydroxypropyl-β-cyclodextrin on freeze-dried and gamma-irradiated PLGA and PLGA-PEG diblock copolymer nanospheres for ophthalmic flurbiprofen delivery. Int. J. Nanomed..

[70] Lu L., Shao X., Jiao Y., Zhou C. (2014). Synthesis of chitosan-graft-β-cyclodextrin for improving the loading and release of doxorubicin in the nanopaticles. J. Appl. Polym. Sci..

[71] Chen S., Wang Y. (2001). Study on β-cyclodextrin grafting with chitosan and slow release of its inclusion complex with radioactive iodine. J. Appl. Polym. Sci..

[72] Song M., Li L., Zhang Y., Chen K., Wang H., Gong R. (2017). Carboxymethyl-β-cyclodextrin grafted chitosan nanoparticles as oral delivery carrier of protein drugs. React. Funct. Polym..

[73] Toeri J., Osorio-Madrazo A., Laborie M.-P. (2017). Preparation and Chemical/Microstructural Characterization of Azacrown Ether-Crosslinked Chitosan Films. Materials.

[74] Wan L., Wang Y., Qian S. (2002). Study on the adsorption properties of novel crown ether crosslinked chitosan for metal ions. J. Appl. Polym. Sci..

[75] Peng C.H., Chen Y.F., Tang M.T. (2003). Synthesis and adsorption properties of chitosan-crown ether resins. J. Cent. South Univ. Technol..

[76] Dimassi S., Tabary N., Chai F., Blachemain N., Martel B. (2018). Sulfonated and sulfated chitosan derivatives for biomedical applications: A review. Carbohydr. Polym..

[77] Rwei S.-P., Chen Y.-M., Lin W.-Y., Chiang W.-Y. (2014). Synthesis and Rheological Characterization of Water-Soluble Glycidyltrimethylammonium-Chitosan. Mar. Drugs.

[78] Tsai H.S., Wang Y.Z., Lin J.J., Lien W.F. (2010). Preparation and Properties of Sulfopropyl Chitosan Derivatives with Various Sulfonation Degree. J. Appl. Polym. Sci..

[79] Zhang X., Sun J. (2020). Synthesis, Characterization, and Properties of Sulfonated Chitosan for Protein Adsorption. Int. J. Polym. Sci..

[80] Sun Z., Shi C., Wang X., Fang Q., Huang J. (2017). Synthesis, characterization, and antimicrobial activities of sulfonated chitosan. Carbohydr. Polym..

[81] Han Z., Zeng Y., Zhang M., Zhang Y., Zhang L. (2016). Monosaccharide compositions of sulfated chitosans obtained by analysis of nitrous acid degraded and pyrazolone-labeled products. Carbohydr. Polym..

[82] Yang J., Luo K., Li D., Yu S., Cai J., Chen L., Du Y. (2013). Preparation, characterization and in vitro anticoagulant activity of highly sulfated chitosan. Int. J. Biol. Macromol..

[83] Wang T., Zhou Y., Xie W., Chen L., Zheng H., Fan L. (2012). Preparation and anticoagulant activity of N-succinyl chitosan sulfates. Int. J. Biol. Macromol..

[84] Sabar S., Aziz H.A., Yusof N.H., Subramaniam S., Foo K.Y., Wilson L.D., Lee H.K. (2020). Preparation of sulfonated chitosan for enhanced adsorption of methylene blue from aqueous solution. React. Funct. Polym..

[85] Martínez-Campos E., Civantos A., Redondo J.A., Guzman R., Perez-Perrino M., Gallardo A., Ramos V., Aranaz I. (2017). Cell Adhesion and Proliferation on Sulfonated and Non-Modified Chitosan Films. AAPS Pharm. Sci. Tech..

[86] Jayakumar R., Selvamurugan N., Nair S.V., Tokura S., Tamura H. (2008). Preparative methods of phosphorylated chitin and chitosan—An overview. Int. J. Biol. Macromol..

[87] Sakaguchi T., Horikoshi T., Nakajima A. (1981). Adsorption of uranium by chitin phosphate and chitosan phosphate. Agric. Biol. Chem..

[88] Amaral I.F., Granja P.L., Barbosa M.A. (2005). Chemical modification of chitosan by phosphorylation: An XPS, FT-IR and SEM study. J. Biomater. Sci..

[89] Tsutsumi A., Sasajima S., Hideshima T., Nishi N., Nishimura S.L., Tokura S. (1986). ESR Studies of Mn(II) Binding to Carboxymethyl and Phosphorylated Chitins in Aqueous Solutions. Polym. J..

[90] Bombaldi de Souza R.F., Bombaldi de Souza F.C., Thorpe A., Mantovani D., Popat K.C., Moraes A.M. (2020). Phosphorylation of chitosan to improve osteoinduction of chitosan/xanthan-based scaffolds for periosteal tissue engineering. Int. J. Biol. Macromol..

[91] Wang X., Ma J., Wang Y., He B. (2002). Bone repair in radii and tibias of rabbits with phosphorylated chitosan reinforced calcium phosphate cements. Biomaterials.

[92] Yokogawa Y., Reyes J.P., Mucalo M.R., Toriyama M., Kawamoto Y., Suzuki T., Nishizawa K., Nagata F., Kamayama T. (1997). Growth of calcium phosphate on phosphorylated chitin fibres. J. Mater. Sci. Mater. Med..

[93] Wang K., Liu Q. (2014). Chemical structure analyses of phosphorylated chitosan. Carbohydr. Res..

[94] Kolawole O.M., Lau W.M., Khutoryanskiy V.V. (2018). Methacrylated chitosan as a polymer with enhanced mucoadhesive properties for transmucosal drug delivery. Int. J. Pharm..

[95] Radhakumary C., Nair P.D., Reghunadhan Nair C.P., Mathew S. (2009). Chitosan-comb-graft-polyethylene glycol monomethacrylate—Synthesis, characterization, and evaluation as a biomaterial for hemodialysis applications. J. Appl. Polym. Sci..

[96] Kumar S., Deepak V., Kumari M., Dutta P.K. (2016). Antibacterial activity of diisocyanate-modified chitosan for biomedical applications. Int. J. Biol. Macromol..

[97] Jaiswal S., Dutta P.K., Kumar S., Koh J., Pandey S. (2019). Methyl methacrylate modified chitosan: Synthesis, characterization and application in drug and gene delivery. Carbohydr. Polym..

[98] Lai J.Y. (2012). Biocompatibility of Genipin and Glutaraldehyde Cross-Linked Chitosan Materials in the Anterior Chamber of the Eye. Int. J. Mol. Sci..

[99] Harish Prashanth K.V., Tharanathan R.N. (2006). Crosslinked chitosan—Preparation and characterization. Carbohydr. Res..

[100] Wahba M.I. (2020). Enhancement of the mechanical properties of chitosan. J. Biomater. Sci..

[101] Pavoni J.M., dos Santos N.Z. (2021). Impact of acid type and glutaraldehyde crosslinking in the physicochemical and mechanical properties and biodegradability of chitosan films. Polym. Bull..

[102] Wang Z., Liu H., Luo W., Cai T., Li Z., Liu Y., Gao W., Wan Q., Wang X., Wang J. (2020). Regeneration of skeletal system with genipin crosslinked biomaterials. J. Tissue Eng..

[103] Pavinatto A., Fiamingo A., De Lacerda Bukzem A., De Souza e Silva D., Martins Dos Santos D., Domiciano Senra T.A., Marcondes Facchinatto W., Campana-Filho S.P., Dotto G.L., Campana-Filho S.P., de Almeida Pinto L.A. (2017). Chemically Modified Chitosan Derivatives. Frontiers in Biomaterials.

[104] Beppu M.M., Vieira R.S., Aimoli C.G., Santana C.C. (2007). Crosslinking of chitosan membranes using glutaraldehyde: Effect on ion permeability and water absorption. J. Membr. Sci..

[105] Muzzarelli R.A.A. (2009). Genipin-crosslinked chitosan hydrogels as biomedical and pharmaceutical aids. Carbohydr. Polym..

[106] Sacco P., Furlani F., De Marzo G., Marsich E., Paoletti S., Donati I. (2018). Concepts for Developing Physical Gels of Chitosan and of Chitosan Derivatives. Gels.

[107] Islam N., Dmour I., Taha M.O. (2019). Degradability of chitosan micro/nanoparticles for pulmonary drug delivery. Heliyon.

[108] Khoerunnisa F., Nurhayati M., Dara F., Rizki R., Nasir M., Aziz H.A., Hendrawan H., Poh N.E., Kaewsaneha C., Opaprakasit P. (2021). Physicochemical Properties of TPP-Crosslinked Chitosan Nanoparticles as Potential Antibacterial Agents. Fibers Polym..

[109] Jonassen H., Kjøniksen A.L., Hiorth M. (2012). Stability of chitosan nanoparticles cross-linked with tripolyphosphate. Biomacromolecules.

[110] Sarkar S.D., Farrugia B.L., Dargaville T.R., Dhara S. (2013). Physico-chemical/biological properties of tripolyphosphate cross-linked chitosan based nanofibers. Mater. Sci. Eng. C.

[111] Silvestro I., Francolini I., Lisio V.D., Martinelli A., Pietrelli L., d’Abusco A.S., Scoppio A., Piozzi A. (2020). Preparation and Characterization of TPP-Chitosan Crosslinked Scaffolds for Tissue Engineering. Materials.

[112] Negm N.A., Hefni H.H.H., Abd-Alaal A.A.A., Badr E.A., Abou Kana M.T.H. (2020). Advancement on modification of chitosan biopolymer and its potential applications. Int. J. Biolog. Macromol..

[113] Ercelen S., Zhang X., Duportail G., Grandfils C., Desbrieres J., Karaeva S., Tikhonov V., Mely Y., Babak V. (2006). Physicochemical properties of low molecular weight alkylated chitosans: A new class of potential nonviral vectors for gene delivery. Colloid Surf. B-Biointerfaces.

[114] Geng Y., Xue H., Zhang Z., Panayi A.C., Knoedler S., Zhou W., Mi B., Liu G. (2023). Recent Advances in Carboxymethyl Chitosan-Based Materials for Biomedical Applications. Carbohydr. Polym..

[115] Anirudhan T.S., Nair A.S., Parvathy J. (2016). Extended Wear Therapeutic Contact Lens Fabricated from Timolol Imprinted Carboxymethyl Chitosan-g-hydroxy Ethyl Methacrylate-g-polyacrylamide as a Onetime Medication for Glaucoma. Eur. J. Pharm. Biopharm..

[116] Zhang X., Qin B., Wang M., Feng J., Zhang C., Zhu C., He S., Liu H., Wang Y., Averick S.E. (2022). Dual pH-Responsive and Tumor-Targeted Nanoparticle-Mediated Anti-Angiogenesis siRNA Delivery for Tumor Treatment. Int. J. Nanomed..

[117] Kurniasih M., Purwati T., Cahyati T., Dewi R.S. (2018). Carboxymethyl chitosan as an antifungal agent on gauze. Int. J. Biol. Macromol..

[118] Chang G., Dang Q., Liu C., Wang X., Song H., Gao H., Sun H., Zhang B., Cha D. (2022). Carboxymethyl chitosan and carboxymethyl cellulose based self-healing hydrogel for accelerating diabetic wound healing. Carbohydr. Polym..

[119] Mourya V.K., Inamdar N.N. (2009). Trimethyl chitosan and its applications in drug delivery. J. Mater. Sci. Mater. Med..

[120] Kebria M.M., Karimi A., Peyravian N., Delattre C., Ghasemian M., Michaud P., Amini N., Roudmiane M.M.M., Milan P.B. (2024). Designing and synthesis of In-Situ hydrogel based on pullulan/carboxymethyl chitosan containing parathyroid hormone for bone tissue engineering. Materialia.

[121] Kim Y.H., Yoon K.S., Lee S.-J., Park E.-J., Rhim J.-W. (2022). Synthesis of Fully Deacetylated Quaternized Chitosan with Enhanced Antimicrobial Activity and Low Cytotoxicity. Antibiotics.

[122] Nezadi M., Keshvari H., Shokrolahi F., Shokrollahi P. (2024). Injectable, self-healing hydrogels based on gelatin, quaternized chitosan, and laponite as localized celecoxib delivery system for nucleus pulpous repair. Int. J. Biol. Macromol..

[123] Asghar B.H., Hassan R.K.A., Bakarat L.A.A., Alharbi A., El Behery M., Elshaarawy R.F.M., Hassan Y.A. (2023). Cross-linked quaternized chitosan nanoparticles for effective delivery and controllable release of O. europaea phenolic extract targeting cancer therapy. J. Drug Deliv. Sci. Technol..

[124] Liu J., Guo S., Jin Z., Zhao K. (2023). Adjuvanted quaternized chitosan composite aluminum nanoparticles-based vaccine formulation promotes immune responses in chickens. Vaccine.

[125] Gao Z., Su C., Wang C., Zhang Y., Wang C., Yan H., Hou G. (2021). Antibacterial and hemostatic bilayered electrospun nanofibrous wound dressings based on quaternized silicone and quaternized chitosan for wound healing. Eur. Polym. J..

[126] Shi D., Shen J., Zhang Z., Shi C., Gu Y., Liu Y. (2019). Preparation and properties of dopamine-modified alginate/chitosan–hydroxyapatite scaffolds with gradient structure for bone tissue engineering. J. Biomed. Mater. Res. Part A.

[127] Xu J., Fang H., Su Y., Kang Y., Xu D., Cheng D.X., Nie Y., Wang H., Liu T., Song K. (2022). 3D bioprinted decellularized extracellular matrix/gelatin/quaternized chitosan scaffold assembling with poly(ionic liquid)s for skin tissue engineering. Int. J. Biol. Macromol..

[128] Tiew S.X., Misran M. (2017). Physicochemical properties of acylated low molecular weight chitosans. Int. J. Polym. Mater. Polym. Biomater..

[129] Al-Remawi M. (2015). Application of N-hexoyl chitosan derivatives with high degree of substitution in the preparation of super-disintegrating pharmaceutical matrices. J. Drug Deliv. Sci. Technol..

[130] Chavanne P., Stevanovic S., Wuthrich A., Braissant O., Pieles U., Gruner P., Schumacher R. (2013). 3D printed chitosan / hydroxyapatite scaffolds for potential use in regenerative medicine. Biomed. Eng.-Biomed. Tech..

[131] Mansour A., Romani M., Acharya A.B., Rahman B., Verron E., Badran Z. (2023). Drug Delivery Systems in Regenerative Medicine: An Updated Review. Pharmaceutics.

[132] Suryani S., Chaerunisaa A.Y., Joni I.M., Ruslin R., Aspadiah V., Anton A., Sartinah A., Ramadhan L.O.A.N. (2024). The Chemical Modification to Improve Solubility of Chitosan and Its Derivatives Application, Preparation Method, Toxicity as a Nanoparticles. Nanotechnol. Sci. Appl..

[133] Chen Q., Qi Y., Jiang Y., Quan W., Luo H., Wu K., Li S., Ouyang Q. (2022). Progress in Research of Chitosan Chemical Modification Technologies and Their Applications. Mar. Drugs.

[134] Lee J.S., Kim H.-S., Nah H., Lee S.J., Moon H.-J., Bang J.B., Lee J.B., Do S.H., Kwon I.K., Heo D.N. (2021). The Effectiveness of Compartmentalized Bone Graft Sponges Made Using Complementary Bone Graft Materials and Succinylated Chitosan Hydrogels. Biomedicines.

[135] Bashir S., Teo Y.Y. (2019). Synthesis and characterization of pH-sensitive N-succinyl chitosan hydrogel and its properties for biomedical applications. J. Chil. Chem. Soc..

[136] Sripetthong S., Eze F.N., Sajomsang W., Ovatlarnporn C. (2023). Development of pH-Responsive *N*-benzyl-*N*-*O*-succinyl Chitosan Micelles Loaded with a Curcumin Analog (Cyqualone) for Treatment of Colon Cancer. Molecules.

[137] Lee J.S., Nah H., Moon H.J., Lee S.J., Heo D.N., Kwon I.K. (2020). Controllable delivery system: A temperature and pH-responsive injectable hydrogel from succinylated chitosan. Appl. Surf. Sci..

[138] Jain N., Rajoriya V., Jain P.K., Jain A.K. (2014). Lactosaminated-*N*-succinyl chitosan nanoparticles for hepatocyte-targeted delivery of acyclovir. J. Nanopart. Res..

[139] Argüelles-Monal W.M., Lizardi-Mendoza J., Fernández-Quiroz D., Recillas-Mota M.T., Montiel-Herrera M. (2018). Chitosan Derivatives: Introducing New Functionalities with a Controlled Molecular Architecture for Innovative Materials. Polymers.

[140] Yoksan R., Matsusaki M., Akashi M., Chirachanchai S. (2004). Controlled hydrophobic/hydrophilic chitosan: Colloidal phenomena and nanosphere formation. Colloid Polym. Sci..

[141] Ubaidulla U., Sultana Y., Ahmed F.J., Khar R.K., Panda A.K. (2007). Chitosan Phthalate Microspheres for Oral Delivery of Insulin: Preparation, Characterization, and In Vitro Evaluation. Drug Deliv..

[142] Karuna D.S., Rathnam G., Ubaidulla U., Ganesh M. (2018). Chitosan phthalate: A novel polymer for the multiparticulate drug delivery system for diclofenac sodium. Adv. Polym. Technol..

[143] Moghaddam F.A., Atyabi F., Dinarvand R. (2009). Preparation and in vitro evaluation of mucoadhesion and permeation enhancement of thiolated chitosan-pHEMA core-shell nanoparticles. Nanomed. Nanotechnol. Biol. Med..

[144] Bernkop-Schnürch A., Hornof M., Zoidl T. (2003). Thiolated polymers-thiomers: Synthesis and in vitro evaluation of chitosan-2-iminothiolane conjugates. Int. J. Pharm..

[145] Ciro Y., Rojas J., Yarce C.J., Salamanca C.H. (2019). Production and Characterization of Glutathione-Chitosan Conjugate Films as Systems for Localized Release of Methotrexate. Polymers.

[146] Qin Y., Li P. (2020). Antimicrobial Chitosan Conjugates: Current Synthetic Strategies and Potential Applications. Int. J. Mol. Sci..

[147] Jin X., Xu Y., Shen J., Ping Q., Su Z., You W. (2011). Chitosan–glutathione conjugate-coated poly (butyl cyanoacrylate) nanoparticles: Promising carriers for oral thymopentin delivery. Carbohydr. Polym..

[148] Li J., Shu Y., Hao T., Wang Y., Qian Y., Duan C., Sun H., Lin Q., Wang C. (2013). A chitosan-glutathione based injectable hydrogel for suppression of oxidative stress damage in cardiomyocytes. Biomaterials.

[149] Manivasagan P., Khan F., Hoang G., Mondal S., Kim H., Doan V.H.M., Kim Y.M., Oh J. (2019). Thiol chitosan-wrapped gold nanoshells for near-infrared laser-induced photothermal destruction of antibiotic-resistant bacteria. Carbohydr. Polym..

[150] Le-Vinh B., Steinbring C., Nguyen N.M., Matuszczak B., Bernkop-Schnurch A. (2023). S-Protected Thiolated Chitosan versus Thiolated Chitosan as Cell Adhesive Biomaterials for Tissue Engineering. ACS Appl. Mater. Interfaces.

[151] Padín-González E., Lancaster P., Bottini M., Gasco P., Tran L., Fadeel B., Wilkins T., Monopoli M.P. (2022). Understanding the Role and Impact of Poly (Ethylene Glycol) (PEG) on Nanoparticle Formulation: Implications for COVID-19 Vaccines. Front. Bioeng. Biotechnol..

[152] Hu Y., Jiang H., Xu C., Wang Y., Zhu K. (2005). Preparation and characterization of poly(ethylene glycol)-g-chitosan with water- and organosolubility. Carbohydr. Polym..

[153] Zhu Y., Gu Z., Liao Y., Li S., Xue Y., Firempong M.A., Xu Y., Yu J., Smyth H.D.C., Xu X. (2022). Improved intestinal absorption and oral bioavailability of astaxanthin using poly(ethylene glycol)-graft-chitosan nanoparticles: Preparation, in vitro evaluation, and pharmacokinetics in rats. J. Sci. Food Agric..

[154] Sultan M.H., Moni S.S., Alqahtani S.S., Bakkari M.A., Lshammari A.A., Almashari Y., Alshahrani S., Madkhali O.A., Mohan S. (2023). Design, physicochemical characterisation, and in vitro cytotoxicity of cisplatin-loaded PEGylated chitosan injectable nano/sub-micron crystals. Saudi Pharm. J..

[155] Chen Y.H., Liu I.J., Lin T.C., Tsai M.C., Hu S.H., Hsu T.C., Wu Y.T., Tzang B.S., Chiang W.C. (2024). PEGylated chitosan-coated nanophotosensitizers for effective cancer treatment by photothermal-photodynamic therapy combined with glutathione depletion. Int. J. Biol. Macromol..

[156] Dawson P.A., Fromm M., Kim R. (2011). Role of the Intestinal Bile Acid Transporters in Bile Acid and Drug Disposition. Drug Transporters. Handbook of Experimental Pharmacology.

[157] Virtanen E., Kolehmainen E. (2004). Use of Bile Acids in Pharmacological and Supramolecular Applications. Eur. J. Org. Chem..

[158] Kim K.S., Youn Y.S., Bae Y.H. (2019). Immune-triggered cancer treatment by intestinal lymphatic delivery of docetaxel-loaded nanoparticle. J. Control. Release.

[159] Yao W., Xu Z., Sun J., Luo J., Wei Y., Zou J. (2021). Deoxycholic acid-functionalised nanoparticles for oral delivery of rhein. Eur. J. Pharm. Sci..

[160] Kim K., Kwon S., Park J.H., Chung H., Jeong S.Y., Kwon I.C., Kim I.S. (2005). Physicochemical characterizations of self-assembled nanoparticles of glycol chitosan-deoxycholic acid conjugates. Biomacromolecules.

[161] Park J.K., Kim T.H., Nam J.P., Park S.C., Park Y.H., Jang M.K., Nah J.W. (2014). Bile Acid Conjugated Chitosan Oligosaccharide Nanoparticles for Paclitaxel Carrier. Macromol. Res..

[162] Pavlović N., Goločorbin-Kon S., Danić M., Stanimirov B., Al-Salami H., Stankov K., Mikov M. (2018). Bile Acids and Their Derivatives as Potential Modifiers of Drug Release and Pharmacokinetic Profiles. Front. Pharmacol..

[163] Arshad M., Sarwar H.S., Sarfaz M., Jalil A., Bin Jardan Y.A., Farooq U., Sohail M.F. (2024). Cholic Acid-Grafted Thiolated Chitosan-Enveloped Nanoliposomes for Enhanced Oral Bioavailability of Azathioprine: In Vitro and In Vivo Evaluation. ACS Omega.

[164] Singh S., Suri R., Tiway A.K., Rana V. (2012). Chitosan films: Crosslinking with EDTA modifies physicochemical and mechanical properties. J. Mater. Sci.-Mater. Med..

[165] Zhou Y., Zhao Y., Han J. (2022). EDTA-chitosan is a feasible conditioning agent for dentin bonding. Clin. Oral Investig..

[166] Netsomboon K., Suchaoin W., Laffleur F., Prufert F., Berkop-Schnurch A. (2017). Multifunctional adhesive polymers: Preactivated thiolated chitosan-EDTA conjugates. Eur. J. Pharm. Biopharm..

[167] Esteso M.A., Romero C.M. (2024). Cyclodextrins: Properties and Applications. Int. J. Mol Sci..

[168] Loftsson T., Brewster M.E. (2012). Cyclodextrins as Functional Excipients: Methods to Enhance Complexation Efficiency. J. Pharm. Sci..

[169] Chaleawlert-Umpon S., Nuchuchua O., Saesoo S., Gonil P., Ruktanonchai U.R., Sajomsang W., Pimpha N. (2011). Effect of citrate spacer on mucoadhesive properties of a novel water-soluble cationic β-cyclodextrin-conjugated chitosan. Carbohydr. Polym..

[170] Harding S.E. (2006). Trends in muco-adhesive analysis. Trends Food Sci. Technol..

[171] Daimon Y., Kamei N., Kawakami K., Takeda-Morishita M., Izawa H., Takechi-Haraya Y., Saito H., Abe M., Ariga K. (2016). Dependence of Intestinal Absorption Profile of Insulin on Carrier Morphology Composed of β-Cyclodextrin-Grafted Chitosan. Mol. Pharm..

[172] Yang Y., Liu Y., Chen S., Cheong K.K., Teng B. (2020). Carboxymethyl β-cyclodextrin grafted carboxymethyl chitosan hydrogel-based microparticles for oral insulin delivery. Carbohydr. Polym..

[173] Pandey A. (2021). Cyclodextrin-based nanoparticles for pharmaceutical applications: A review. Environ. Chem. Lett..

[174] Norouzi Z., Abdouss M. (2023). Electrospun nanofibers using β-cyclodextrin grafted chitosan macromolecules loaded with indomethacin as an innovative drug delivery system. Int. J. Biol. Macromol..

[175] Hao P.Y., Zhou H.Y., Ren L.J., Zheng H.J., Tong J.N., Chen Y.W., Park H.J. (2023). Preparation and antibacterial properties of curcumin-loaded cyclodextrin-grafted chitosan hydrogel. J. Sol.-Gel Sci. Technol..

[176] Lee S.J., Nah H., Ko W.K., Lee D., Moon H.J., Heo M., Hwang Y.S., Bang J.B., An S.H., Heo D.N. (2021). Preparation of β-Cyclodextrin-grafted Chitosan Electrospun Nanofibrous Scaffolds as a Hydrophobic Drug Delivery Vehicle for Tissue Engineering Applications. ACS Omega.

[177] Yi Y., Wang Y., Liu H. (2003). Preparation of new crosslinked chitosan with crown ether and their adsorption for silver ion for antibacterial activities. Carbohydr. Polym..

[178] Murali S., Aparna V., Suresh M.K., Biswas R., Jayakumar R., Sathianarayanan S. (2018). Amphotericin B loaded sulfonated chitosan nanoparticles for targeting macrophages to treat intracellular Candida glabrata infections. Int. J. Biol. Macromol..

[179] Gao Y., Liu W., Wang W., Zhang X., Zhao X. (2018). The inhibitory effects and mechanisms of 3,6-O-sulfated chitosan against human papillomavirus infection. Carbohydr. Polym..

[180] Gagliardi A., Giuliano E., Venkateswararao E., Fresta M., Bulotta S., Awasthi V., Cosco D. (2021). Biodegradable Polymeric Nanoparticles for Drug Delivery to Solid Tumors. Front. Pharmacol..

[181] Wang X.H., Tian Q., Wang W., Zhang C.N., Wang P., Yuan Z. (2012). In vitro evaluation of polymeric micelles based on hydrophobically-modified sulfated chitosan as a carrier of doxorubicin. J. Mate. R Sci.-Mater. Med..

[182] Ji L., Yu Y., Zhu F., Huang D., Wang X., Wang J., Liu C. (2024). 2-N, 6-O sulfated chitosan evokes periosteal stem cells for bone regeneration. Bioact. Mater..

[183] Jayakumar R., Nagahama H., Furuike T., Tamura H. (2008). Synthesis of phosphorylated chitosan by novel method and its characterization. Int. J. Biol. Macromol..

[184] Chen Y., Chen Y., Lu D., Qiu Y. (2022). Synthesis of a Novel Water-Soluble Polymer Complexant Phosphorylated Chitosan for Rare Earth Complexation. Polymers.

[185] Anushree U., Punj P., Vasumathi, Bharati S. (2023). Phosphorylated chitosan accelerates dermal wound healing in diabetic wistar rats. Glycoconj. J..

[186] Hamai R., Maeda H., Sawai h., Shirosaki Y., Kasuga T., Miyazaki T. (2018). Structural effects of phosphate groups on apatite formation in a copolymer modified with Ca^2+^ in a simulated body fluid. J. Mater. Chem. B.

[187] Wenxiu L., Guojiang H., Liing Q., Wenli D., Baoqin H., Liming J., Yan Y. (2024). Fabrication of bioactive glass/phosphorylated chitosan composite scaffold and its effects on MC3T3-E1 cells. Biomed. Mater..

[188] Gogoi P., Dutta A., Ramteke A., Maji T.K. (2020). Preparation, characterization and cytotoxic applications of curcumin-(±) α-lipoic acid coloaded phosphorylated chitosan nanoparticles in MDA MB 231 breast cancer cell line. Polym. Adv. Technol..

[189] Wei J., Xue W., Yu X., Qiu X., Liu Z. (2017). pH Sensitive phosphorylated chitosan hydrogel as vaccine delivery system for intramuscular immunization. J. Biomater. Appl..

[190] Bettencourt A., Almeida A.J. (2012). Poly(methyl methacrylate) particulate carriers in drug delivery. J. Microencapsul..

[191] Thang N.H., Chien T.B., Cuong D.X. (2023). Polymer-Based Hydrogels Applied in Drug Delivery: An Overview. Gels.

[192] Anraku M., Gebicki J.M., Iohara D., Tomida H., Uekama K., Maruyama T., Hirayama F., Otagiri M. (2018). Antioxidant activities of chitosans and its derivatives in in vitro and in vivo studies. Carbohydr. Polym..

[193] Liu Z., Zhang M., Wang Z., Wang Y., Dong W., Ma W., Zhao S., Sun D. (2022). 3D-printed porous PEEK scaffold combined with CSMA/POSS bioactive surface: A strategy for enhancing osseointegration of PEEK implants. Compos. Part B-Eng..

[194] Senthil K., Kalpana R., Kumar V. (2024). Effect of Dextrose Cross-Linked Glutaraldehyde Hydrogel on Wound Healing Activity. J. Pharm. Bioallied. Sci..

[195] Monteiro O.A., Airoldi C. (1999). Some studies of crosslinking chitosan–glutaraldehyde interaction in a homogeneous system. Int. J. Biol. Macromol..

[196] Ahmed B.H., Manel M., Mohamed D., Jalel D. (2017). Glutaraldehyde test for the rapid diagnosis of pulmonary and extra-pulmonary tuberculosis in an area with high tuberculosis incidence. Mem. Inst. Oswaldo Cruz..

[197] Banafati Zadeh F., Zamanian A. (2022). Glutaraldehyde: Introducing Optimum Condition for Cross-linking the Chitosan/Gelatin Scaffolds for Bone Tissue Engineering. Int. J. Eng..

[198] Nayak U.Y., Gopal S., Mutalik S., Ranjith A.K., Reddy M.S., Gupta P., Udupa N. (2009). Glutaraldehyde cross-linked chitosan microspheres for controlled delivery of zidovudine. J. Microencapsul..

[199] Cai Y., Lapitsky Y. (2017). Analysis of chitosan/tripolyphosphate micro- and nanogel yields is key to understanding their protein uptake performance. J. Colloid Interface Sci..

[200] Hoang N.H., Le Thanh T., Sangpueak R., Treekoon J., Saengchan C., Thepbandit W., Papathoti N.K., Kamkaew A., Buensanteai N. (2022). Chitosan Nanoparticles-Based Ionic Gelation Method: A Promising Candidate for Plant Disease Management. Polymers.

[201] Valadi M., Doostan M., Khoshnevisan K., Doostan M., Maleki H. (2024). Enhanced healing of burn wounds by multifunctional alginate-chitosan hydrogel enclosing silymarin and zinc oxide nanoparticles. Burns.

[202] Doostan M., Doostan M., Mohammadi P., Khoshnevisan K., Maleki H. (2023). Wound healing promotion by flaxseed extract-loaded polyvinyl alcohol/chitosan nanofibrous scaffolds. Int. J. Biol. Macromol..

[203] Dinu M.V., Gradinaru A.C., Lazar M.M., Dinu I.A., Raschip I.E., Ciocarlan N., Aprotosoaie A.C. (2021). Physically cross-linked chitosan/dextrin cryogels entrapping Thymus vulgaris essential oil with enhanced mechanical, antioxidant and antifungal properties. Int. J. Biol. Macromol..

[204] Maleki H., Doostan M., Khoshnevisan K., Baharifar H., Maleki S.A., Fatahi M.A. (2024). Zingiber officinale and thymus vulgaris extracts co-loaded polyvinyl alcohol and chitosan electrospun nanofibers for tackling infection and wound healing promotion. Heliyon.

[205] Amalraj A., Haponiuk J.T., Thomas S., Gopi S. (2020). Preparation, characterization and antimicrobial activity of polyvinyl alcohol/gum arabic/chitosan composite films incorporated with black pepper essential oil and ginger essential oil. Int. J. Biol. Macromol..

[206] Edo G.I., Yousif E., Al-Mashhadani H. (2024). Chitosan: An overview of biological activities, derivatives, properties, and current advancements in biomedical applications. Carbohydr. Res..

[207] ClinicalTRials.gov. https://clinicaltrials.gov.

[208] Kantak M.N., Bharate S.S. (2022). Analysis of clinical trials on biomaterial and therapeutic applications of chitosan: A review. Carbohydr. Polym..

